# Platinum(II) Complexes of Nonsymmetrical *NCN*-Coordinating Ligands: Unimolecular and Excimeric Luminescence Properties and Comparison with Symmetrical Analogues

**DOI:** 10.1021/acs.inorgchem.3c01439

**Published:** 2023-07-27

**Authors:** Rebecca J. Salthouse, Amit Sil, Louise F. Gildea, Dmitry S. Yufit, J. A. Gareth Williams

**Affiliations:** Department of Chemistry, Durham University, South Road, Durham DH1 3LE, U.K.

## Abstract

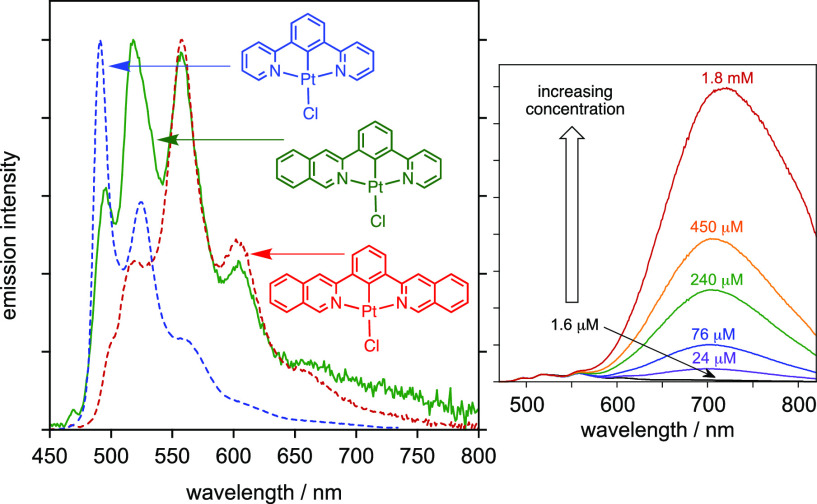

A series of seven new platinum(II) complexes PtL^*n*^Cl have been prepared, where L^*n*^ is an *NCN*-coordinating ligand comprising a benzene ring 1,3-disubstituted with two different azaheterocycles. In PtL^1–5^Cl, one heterocycle is a simple pyridine ring, while the other is an isoquinoline, a quinoline, a pyrimidine (L^1^, L^2^, L^3^), or a *p*-CF_3_- or *p*-OMe-substituted pyridine (L^4^ and L^5^). PtL^6^Cl incorporates both a *p*-CF_3_ and a *p*-OMe-substituted pyridine. The synthesis of the requisite proligands HL^*n*^ is achieved using Pd-catalyzed cross-coupling methodology. The molecular structures of six of the Pt(II) complexes have been determined by X-ray diffraction. All the complexes are brightly luminescent in deoxygenated solution at room temperature. The absorption and emission properties are compared with those of the corresponding symmetrical complexes featuring two identical heterocycles, PtL^*n*sym^Cl, and of the parent Pt(dpyb)Cl containing two unsubstituted pyridines [dpybH = 1,3-di(2-pyridyl)benzene]. While the absorption spectra of the nonsymmetrical complexes show features of both PtL^*n*sym^Cl and Pt(dpyb)Cl, the emission generally resembles that of whichever of the corresponding symmetrical complexes has the lower-energy emission. PtL^1^Cl differs in that—at room temperature but not at 77 K—it displays emission bands that can be attributed to excited states involving both the pyridine and the isoquinoline rings, despite the latter being unequivocally lower in energy. This unusual behavior is attributed to thermally activated repopulation of the former excited state from the latter, facilitated by the very long-lived nature of the isoquinoline-based excited state. At elevated concentrations, all the complexes show an additional red-shifted emission band attributable to excimers. For PtL^1^Cl, the excimer strikingly dominates the emission spectra at all but the lowest concentrations (<10^–5^ M). Trends in the energies of the excimers and their propensity to form are compared with those of the symmetrical analogues.

## Abstract 2

2,6-Dipyridylbenzene derivatives are established ligands for brightly luminescent Pt(II) complexes. We present a study of nonsymmetric systems of this type, where the two heterocycles are different from one another. The emission generally emanates from an excited state involving the heterocycle with the lower-energy π* orbitals, but an isoquinoline complex shows dual emission at room temperature. Isoquinoline also favors the efficient formation of very bright excimers that dominate the emission spectrum at all but the lowest concentrations.

## 1 Introduction

The luminescence properties of cyclometalated platinum(II) complexes based on *ortho*-aryl-heterocycles are of interest in a wide range of applications that include light-emitting diodes,^1^ chemosensing,^2^ and bioimaging.^3^ The phosphorescent nature of the emission in such systems leads to utility in harvesting triplet states in organic light-emitting devices (OLEDs),^4^ while the longer lifetimes compared to fluorescence renders such systems amenable to time-gated detection procedures in which background emission is effectively eliminated.^5^ While closely related iridium(III) complexes may be superior in many respects, Pt(II) systems do offer some rather unique features. These d^8^ complexes are typically square-planar, and face-to-face interactions between them may lead to the formation of aggregates or excimers whose excited states are stabilized relative to those of the isolated molecules.^6^ The resulting red-shifted emission offers an interesting potential strategy for achieving more efficient red or near-infrared emitters.^7^ Meanwhile, the combination of such red-emitting bimolecular states with the green-blue emission of the monomeric excited states offers a means of generating white light emission using a single dopant in an OLED.^8^ The match between the “double-humped” emission spectra of excimer-forming Pt(II) complexes and the photosynthetic action spectrum has also been noted: the efficiency of OLEDs for plant growth can accordingly be optimized.^9^

**Figure 1:**
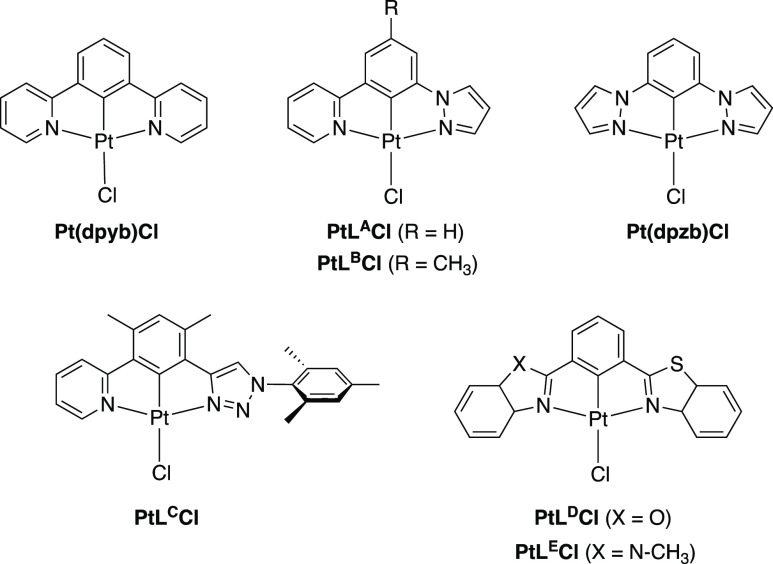
Reported examples of Pt(II) complexes PtL^A–E^Cl with nonsymmetric *NCN*-coordinating ligands, together with the structure of the parent, symmetric complex Pt(dpyb)Cl and of Pt(dpzb)Cl.

By no means all emissive Pt(II) complexes form emissive bimolecular states. In some cases, emission is simply quenched with an increasing concentration. Efficient excimer emission is well-established in the class of Pt(II) complex based on tridentate, *NCN*-coordinating ligands, of which Pt(dpyb)Cl may be considered the parent [Figure 1; dpybH = 1,3-di(2-pyridyl)benzene].^10^ This complex displays very bright phosphorescence in dilute solution, with λ(0,0) = 493 nm in CH_2_Cl_2_ at room temperature, but at higher concentrations, a broad band centered at approximately 700 nm appears in the spectrum, attributed to an excimer. The intensity and lifetime of the monomer emission are correspondingly reduced as the excimer contribution increases. Time-resolved studies show that the excimer emission grows in over a period of a few hundred nanoseconds before decaying with a lifetime similar to the unimolecular emission.^6c,11^ In OLEDs employing Pt(dpyb)Cl or its derivatives as emitters, doped into materials like PVK at around 20% by mass, the devices show efficient generation of white light due to the combination of blue-green unimolecular and red excimer-like emission in the spectrum. In neat (100%) films, uniquely excimer-like electroluminescence is observed from the devices, with a single emission band around 700 nm.^12^

The singlet and triplet excited-state energies of cyclometalated complexes can now often be rationalized well using time-dependent density functional theory (DFT).^13^ On the other hand, the factors that influence the energy of excimers formed by such systems, or their propensity to form, are generally not yet well accounted for. In the case of Pt(dpyb)Cl derivatives, we have shown—empirically—that electron-donating substituents such as OMe or NMe_2_*para* to the nitrogen atoms in the pyridine rings lead to a blue shift in the excimer relative to the parent, while electron-withdrawing CF_3_ substituents red-shift the excimer, albeit with a concomitant large decrease in intensity.^14,15^ In contrast, substituents in the central aryl ring *para* to the Pt(II) ion have little effect on the excimer energy, yet they reduce the propensity for excimers to form.^10b^ Evidently, the formation of excimers and aggregates—as well as the energy of their excited states—is influenced by both electronic and steric factors.

Almost all of the reported *NCN*-coordinated complexes of Pt(II) reported to date feature symmetric tridentate ligands, that is, ones where the two lateral heterocycles are the same.^10,14–17^ We previously reported on a derivative of Pt(dpyb)Cl in which one of the pyridyl rings was replaced by a pyrazole ring, PtL^A^Cl (Figure 1).^18^ In dilute solution, the emission properties were essentially identical to those of the parent. For example, λ(0,0) = 491 nm in CH_2_Cl_2_, compared to 493 nm for the parent, which contrasts with a substantial blue shift to 451 nm for the bis-pyrazolyl analogue Pt(dpzb)Cl. This result is readily rationalized in that the HOMO in Pt(NCN)Cl complexes is located on the central aryl ring, the metal, and the chloride whereas the LUMO is largely localized on the heterocycles. Thus, when there are two different heterocycles present, the LUMO is expected to localize to that with the lowest-energy π* orbitals, namely—in the case of PtL^A^Cl—the pyridine ring as opposed to the more electron-rich pyrazole. The excited-state energy is accordingly similar to that of the symmetric complex with two pyridine rings, Pt(dpyb)Cl, and not like Pt(dpzb)Cl, which features two more electron-rich pyrazoles only. On the other hand, the excimer displayed by PtL^A^Cl is significantly blue-shifted relative to that of Pt(dpyb)Cl (550 vs 700 nm in CH_2_Cl_2_). This result indicates that the presence of the electron-rich pyrazole ring destabilizes the excited state of the bimolecular excimer, even though the unimolecular excited state is essentially unaffected. Meanwhile, the presence of a methyl substituent in the central ring (PtL^B^Cl) blocks excimer formation altogether at accessible concentrations. Aside from this result, there is very little information on nonsymmetric systems. Two other studies did reveal a similar trend in the unimolecular emission, namely the emission energy being determined by the heterocycle with the lower-energy π* orbital—the pyridine as opposed to the triazole in PtL^C^Cl and the benzothiazole rather than the benzoxazole or benzimidazole in PtL^D^Cl and PtL^E^Cl, respectively.^19,20^ However, in neither case was excimer emission reported.

The main objectives of the present work were: (1) to establish a general synthetic strategy to nonsymmetric *NCN*-coordinating ligands using Pd-catalyzed cross-couplings; (2) to explore analogues of the PtL^A^Cl structure that feature heterocycles (in place of the pyrazole) known to have a profound influence on excimer formation in the complexes of the corresponding symmetric ligands; and (3) to investigate how a “donor–acceptor” arrangement in a bis-pyridyl complex might influence the excimer energy and/or its propensity to form (i.e., in a complex incorporating an electron-donating group in one pyridyl ring and an electron-accepting group in the other).

## 2 Results and Discussion

### 2.1 Selection of Complexes for Study

**Figure 2:**
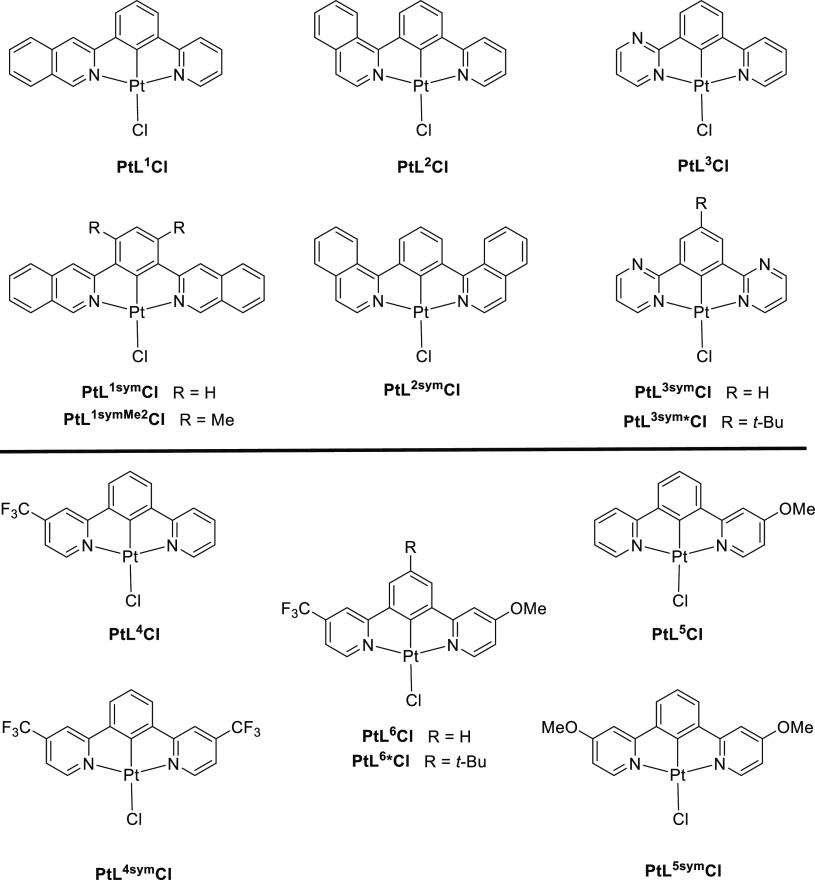
Complexes PtL^1–6^Cl investigated in this work featuring nonsymmetric *NCN*-coordinating ligands, together with those of corresponding symmetrically substituted ligands.

Complexes PtL^1–3^Cl (Figure 2) were selected to explore the influence of the heterocycle. The complex PtL^1^Cl, incorporating 3-substituted isoquinoline, was of interest as we had discovered that the corresponding symmetric complex PtL^1sym^Cl has an exceptionally high propensity to excimer formation (vide infra). The isomeric complex featuring 2-substituted isoquinolines, PtL^2sym^Cl, behaved differently, displaying red-shifted unimolecular emission and red-shifted (albeit very weak) excimer emission^6c^ and so its nonsymmetric analogue, PtL^2^Cl, was also targeted. The bis-pyrimidine complex PtL^3sym^*Cl has recently been found to generate highly red-shifted emission around 800 nm in OLEDs through the formation of aggregates and so the nonsymmetric complex PtL^3^Cl featuring one pyrimidine and one pyridine ring was deemed worthy of exploration.^7d,21^

Meanwhile, with a view to addressing the third objective listed at the end of the introduction, we targeted the complex PtL^6^Cl that incorporates an electron-donating methoxy substituent in one pyridine ring and an electron-withdrawing trifluoromethyl group in the other (Figure 2). Comparison with the corresponding symmetric complexes PtL^4sym^Cl and PtL^5sym^Cl may then reveal whether the presence of “complementary” electron-rich and electron-poor pyridines influences the excimer or whether the behavior would essentially be the same as one of the two symmetrically substituted systems. As part of this study, we also examined the corresponding complexes incorporating one unsubstituted pyridine ring, PtL^4^Cl (with one CF_3_-substituted pyridine) and PtL^5^Cl (one MeO-substituted pyridine), as well as a *t*-butyl-appended derivative of PtL^6^Cl which will be referred to as PtL^6^*Cl.

### 2.2 Synthesis of the Proligands and the Pt(II) Complexes

**Scheme 1:**
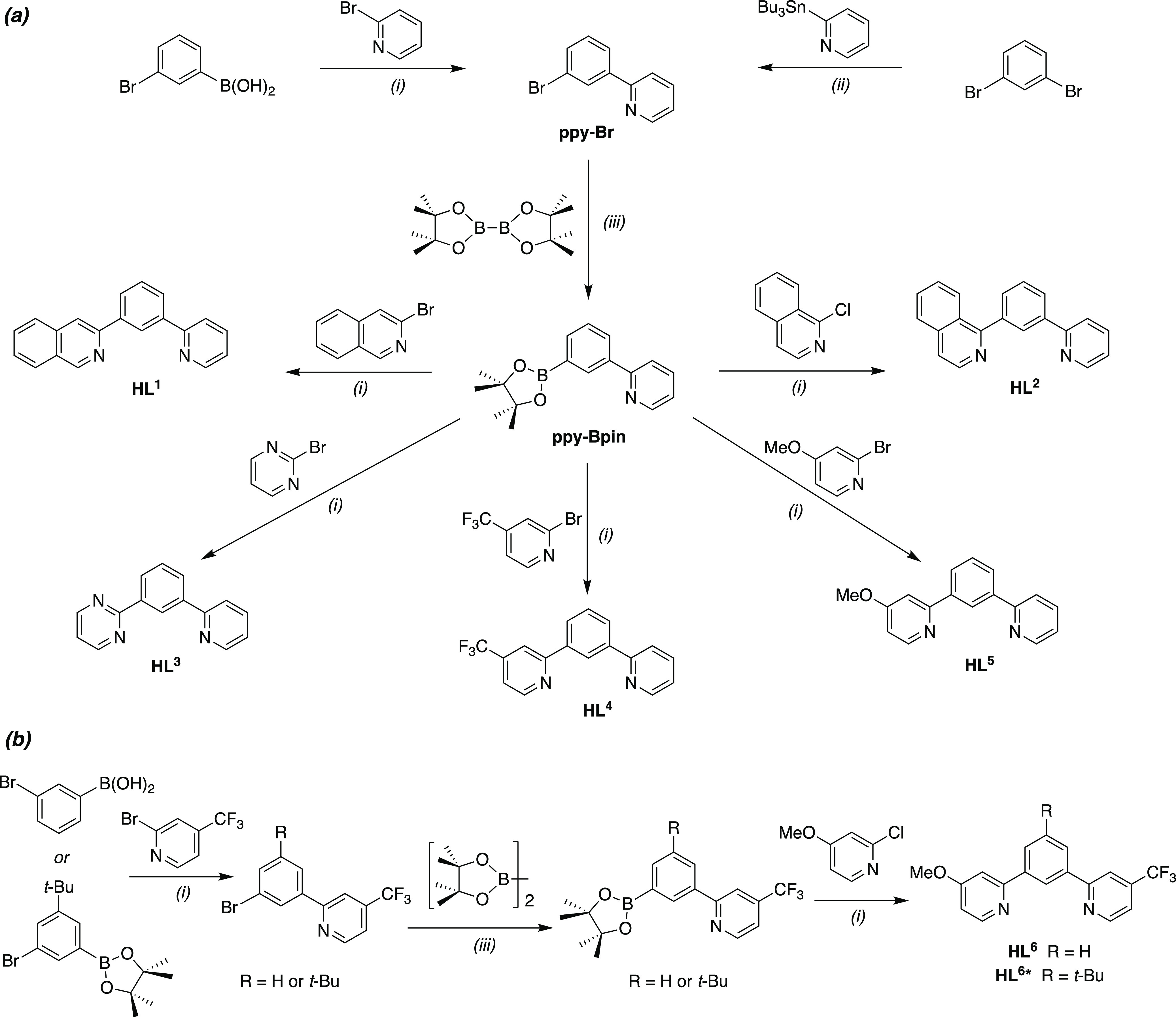
Synthesis of the Proligands (a) HL^1–5^ and (b) HL^6^ by Palladium-Catalyzed Cross-Coupling Reactions Catalysts and conditions: (i) Pd(PPh_3_)_4_, Na_2_CO_3_ (aq), dimethoxyethane, 85 °C; (ii) Pd(PPh_3_)_2_Cl_2_, LiCl, toluene, 110 °C; and (iii) Pd(dppf)Cl_2_, KOAc, dioxane, 80 °C.

The synthesis of symmetric *NC*(*H*)*N* proligands is typically carried out by Pd-catalyzed cross-coupling reactions.^22^ Early reports made use of the cross-coupling of organotin or organozinc reagents (e.g., py–SnR_3_ or py–ZnCl) with 1,3-dibromobenzene under Stille or Negishi conditions, respectively.^23,24^ Suzuki coupling of 1,3-benzene-diboronic acid with halogenated heterocycles offers an attractive alternative, owing to the lower sensitivity to oxygen and/or water, to the benign nature of the borate side products, and to the divergence offered by the wide range of heterocycles available without recourse to the introduction of tin, zinc, or protected boron functionality ortho to the heteroatom. In the present instance of targeting nonsymmetric derivatives incorporating one unsubstituted pyridine ring, the desirable intermediate is thus 3-(2-pyridyl)benzene boronic acid. This compound was prepared as its pinacolate ester, **ppy-Bpin** (Scheme 1), in good yield from 3-(2-pyridyl)bromobenzene (**ppy-Br**) upon Pd-catalyzed coupling with bispinacolato diboron. The intermediate **ppy-Br** was in turn readily synthesized by either Suzuki or Stille coupling^25^ (see the Supporting Information). The proligands HL^1–5^ were then obtained in one step from **ppy-Bpin** upon Suzuki coupling with the appropriate *ortho*-bromo or *ortho*-chloro heterocycle in the presence of sodium carbonate, using Pd(PPh_3_)_4_ as the catalyst. The CF_3_/OMe proligand HL^6^ was obtained in a similar way to HL^5^ but using 2-bromo-4-(trifluoromethyl)-pyridine in place of 2-bromopyridine in the first Suzuki coupling step with 3-bromobenzene boronic acid. The *t*-butyl derivative HL^6^* was prepared by an analogous route from the pinacolate ester of 3-bromo-5-*t*-butyl-benzene boronic acid.

The subsequent complexation of the ligands with Pt(II) was carried out by reaction of the proligands with K_2_PtCl_4_ in acetic acid for 60 h under an inert atmosphere, as described previously for Pt(dpyb)Cl and several of its derivatives.^17,26^ The complexes were precipitated from solution and were isolated by centrifugation and purified through a sequence of washings and recrystallizations.

### 2.3 Molecular and Crystal Structures

**Figure 3:**
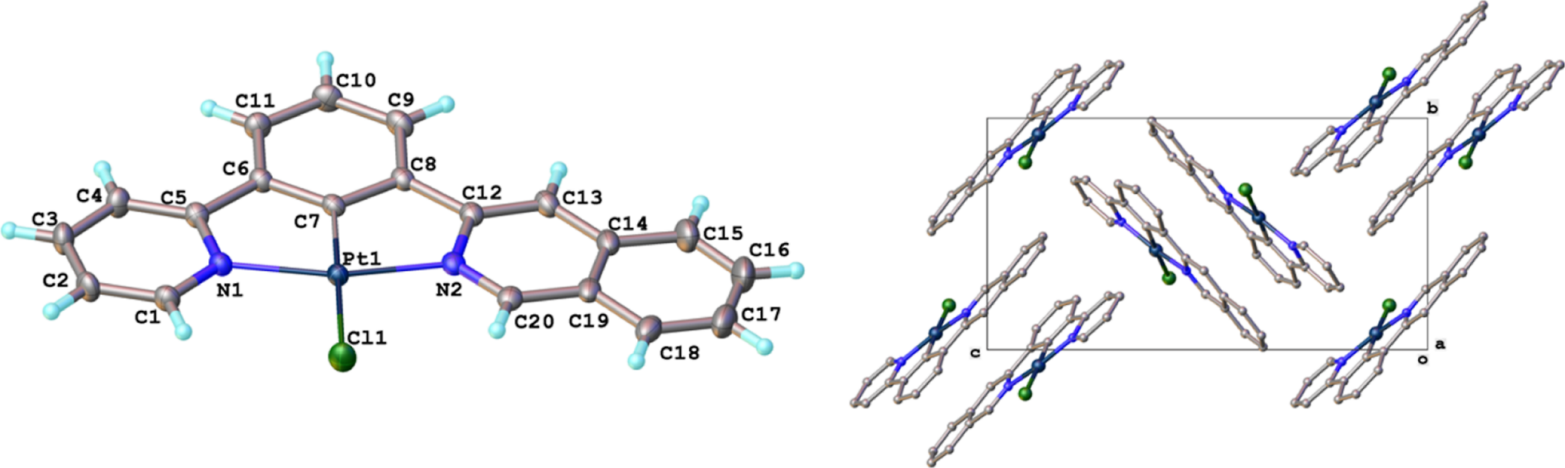
Crystal structure of PtL^1^Cl obtained by X-ray diffraction. Note the orientation of the molecules within the pairs: head-to-tail and with the isoquinoline of one molecule positioned above the pyridine ring of the other. See Figures S1 to S7 for the structures of the other complexes.

Crystals of all the nonsymmetrical complexes (apart from PtL^5^Cl) were obtained that were suitable for X-ray diffraction analysis. The representative example of PtL^1^Cl is shown in Figure 3, with other complexes in Figures S2 to S7 (Supporting Information). As expected, the molecular structures show roughly planar geometries around the Pt(II) centers and of the constituent aromatic rings relative to one another. All of the complexes pack in such a way that the planes of the molecules are parallel to those of their nearest neighbors, as typically observed for cyclometalated Pt(II) complexes. For PtL^1–4^Cl, containing one unsubstituted pyridine, the interplanar distances are in the range of 3.3–3.5 Å, indicative of π–π interactions (Table S1). The distance is a little longer for the doubly substituted pyridine complex PtL^6^Cl and longer still for the *t*-butyl analogue [3.756(2) and 4.147(1) Å, respectively], presumably due to the bulky nature of the *t*-butyl group disfavoring a closer approach. There is no evidence of metallophilic interactions in any of these structures: the Pt···Pt distances are all >5 Å, substantially longer than the interplanar distances. This trend of interactions between the aromatic rings—rather than between the platinum centers—is quite typical of previously crystallized symmetrical Pt(*NCN*)Cl complexes and contrasts with the metallophilic interactions found in many Pt(II) complexes of aromatic ligands. There are, nevertheless, some differences between the five structures, notably the molecules of PtL^1^Cl and PtL^4^Cl being arranged into dimers rather than the usual aromatic stacks of the others.

In Pt(*NCN*)Cl structures studied previously, examples of both head-to-tail (H–T) and head-to-head (H–H) arrangements of neighboring molecules have been observed (and occasionally orientations in between).^12^ We reported the structures of PtL^2sym^Cl and PtL^3sym^*Cl previously: they show head-to-tail arrangements.^6c^ Although no suitable crystals of PtL^1sym^Cl have hitherto been obtained, a structure of a derivative of this complex, featuring two methyl substituents in the central ring *meta* to the metal, has been obtained during the present work, which we shall refer to as PtL^1symMe2^Cl (Figure 2). It, too, shows the H–T disposition of neighboring complexes (Figure S1). Similarly, the nonsymmetric complexes PtL^1–3^Cl all show such an H–T arrangement. On the other hand, in the symmetric complex PtL^4sym^Cl, neighboring molecules are staggered relative to one another with an angle of around 60° between the Pt–Cl bonds (rather than the 180° of the H–T), such that the central phenyl ring of one complex lies above a pyridyl ring of its nearest neighbor. Unusual spiro centrosymmetric tetramers form in the crystal of this complex, and it is the only one among all the complexes in this study with rather short Pt···Pt distances [3.3884(5) and 3.9995(6) Å]. In contrast, its nonsymmetric analogue PtL^4^Cl adopts the H–T arrangement as do the CF_3_/OMe-substituted complexes PtL^6^Cl and PtL^6^*Cl.

Perhaps the most important point to note, though, is that in each of the nonsymmetric complexes, the molecules of adjacent H–T pairs are oriented trans relative to one another about the {Pt–Cl}_2_ plane; in other words, the unsubstituted pyridine ring of one molecule is positioned above the quinoline/pyrimidine/substituted pyridine ring of the other (for PtL^1–5^Cl). Similarly, for PtL^6^Cl and PtL^6^*Cl, the MeO-substituted ring of one molecule is positioned above the CF_3_-substituted ring of the other. This suggests some element of complementarity in the π–π interactions between the constituent rings of neighboring molecules and thus that face-to-face intermolecular interactions might indeed possibly be favored by the nonsymmetric structure relative to the symmetric analogues.

### 2.4 Absorption spectra

**Figure 4:**
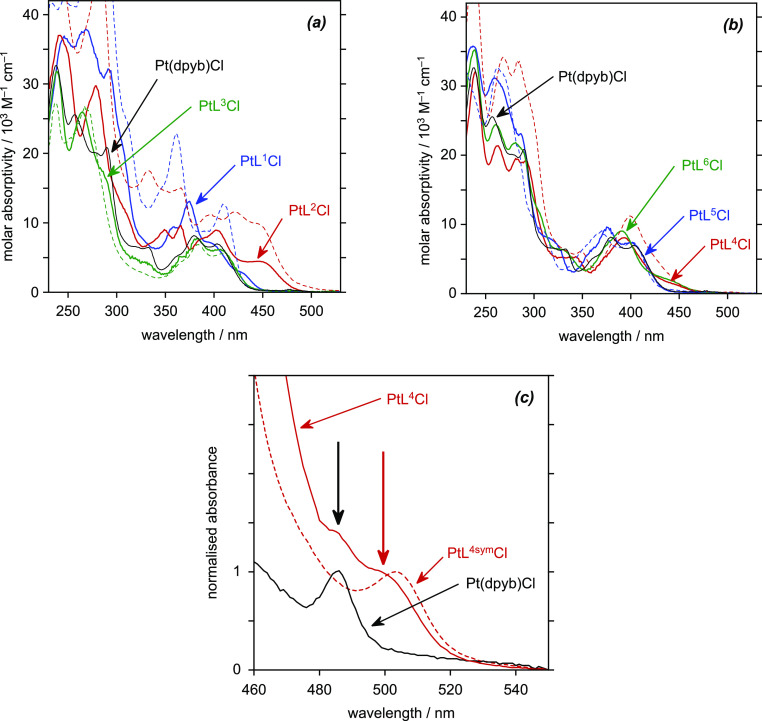
Absorption spectra in CH_2_Cl_2_ at 295 K: (a) 3-isoquinoline, 1-isoquinoline, and pyrimidine-containing complexes PtL^1^Cl, PtL^2^Cl, and PtL^3^Cl (blue, red, and green solid lines, respectively) and symmetric analogues PtL^1sym^Cl, PtL^2sym^Cl, and PtL^3sym^Cl (corresponding dashed lines); (b) para-substituted pyridine complexes PtL^4^Cl, PtL^5^Cl, and PtL^6^Cl (red, blue, and green solid lines, respectively) and symmetric analogues PtL^4sym^Cl and PtL^5sym^Cl (corresponding dashed lines); and (c) expansion of the low-energy region of PtL^4^Cl and PtL^4sym^Cl. The spectrum of Pt(dpyb)Cl is also shown in each plot (black solid line).

The absorption spectra of the nonsymmetric complexes in dichloromethane solution at room temperature are shown in Figure 4, together with the spectra of their symmetric analogues and of the parent Pt(dpyb)Cl under the same conditions for comparison. The spectra are typical of cyclometalated Pt(II) complexes (and indeed also of related Ir(III) complexes with cyclometallating ligands^27^), with sets of intense bands at λ < 330 nm, attributed to intraligand π → π* transitions, and somewhat weaker bands at longer wavelengths extending into the visible region, assigned to charge–transfer transitions of mixed ^1^[d_Pt_ |π_L_ → π_L_*] character.^13a^ In such transitions, the filled orbitals are typically based on the metal and metalated phenyl ring while the acceptor π* orbitals are localized to the heterocycle.^11^ Thus, the introduction of heterocycles that are more electron-deficient than pyridine (with lower-energy π* orbitals) typically leads to red shifts in lowest-energy absorption bands and vice versa. Considering first the complexes in which the heterocycles are both pyridine rings, PtL^4–6^Cl (Figure 4b), it can be seen that the two 4-(trifluoromethyl)-pyridine-containing complexes PtL^4^Cl and PtL^6^Cl show an absorption tail or shoulder extending further into the red than Pt(dpyb)Cl, a feature shared with the symmetric CF_3_ complex PtL^4sym^Cl and noted previously.^15^ Evidently, the lowest-energy excited state in PtL^4^Cl and PtL^6^Cl is associated with a charge-transfer transition to the more electron-deficient, CF_3_-substituted pyridine in each case, as might be expected. DFT calculations carried out on PtL^4^Cl as a representative example confirm this interpretation (see the Supporting Information). The HOMO is based on the metalated aryl ring, Pt, and the halide while the LUMO is seen to be localized largely on the trifluoromethyl-substituted pyridine and not on the unsubstituted pyridine (Figure S24). A similar calculation as part of previous work on the pyrazole–pyridine complex PtL^A^Cl (Figure 1) likewise revealed the LUMO to be based on the pyridine rather than the pyrazole ring.^18^

In contrast to PtL^4^Cl and PtL^6^Cl, the low-energy portion of the spectrum of the methoxy-substituted PtL^5^Cl is quite similar to that of Pt(dpyb)Cl. Using the same reasoning, it seems likely that the lowest-energy excited state in this case involves the unsubstituted pyridine. Indeed, to a reasonable approximation, the spectra of the three nonsymmetric complexes PtL^4–6^Cl are similar to spectra simulated from the weighted sum of the corresponding symmetric complexes (see Supporting Information, Figure S8). The nonconjugated meta relationship between the pyridine rings appended to the central aryl ring thus apparently ensures that excited states associated with both “halves” of the *NCN* unit are present at similar energies to those in the corresponding symmetric complexes. We can usefully formulate them as ^1^[d_Pt_ |π_N′CN″_ → π_N′CN″_*] and ^1^[d_Pt_ |π_N′CN″_ → π_N′CN″_*]. Finally, we note that the only differences between the spectra of PtL^6^Cl and its *t*-butyl derivative PtL^6^*Cl are a small red shift and slight increase in molar absorptivity in the latter (Figure S9a), in line with original observations on the methyl derivative of Pt(dpyb)Cl.^10a^ The same trend is also observed if we compare PtL^3sym^Cl and its *t*-butyl derivative PtL^3sym^*Cl (Figure S9b).

While the absorption spectra of cyclometalated Pt(II) complexes are dominated by spin-allowed transitions, the formally spin-forbidden transition to the lowest-energy triplet states(s) may sometimes be discerned. Such is the case for Pt(dpyb)Cl, where the direct S_0_ → T_1_ transition has been reported to appear as a sharp band at 485 nm in CH_2_Cl_2_.^10a^ Careful inspection of the spectra of concentrated solutions of all the new complexes PtL^1–7^Cl reveals a similar weak band or bands to low energy of the main bands, with ε around 100 M^–1^ cm^–1^ (Table 1). Given the initial conclusion above, that the nonsymmetric complexes feature singlet excited states associated with the two different heterocycles, one might then predict two such low-energy bands associated with transitions to spin-forbidden ^3^[d_Pt_ |π_N′CN″_ → π_N′CN″_*] and ^3^[d_Pt_ |π_N′CN″_ → π_N′CN″_*] states. Unfortunately, the proximity of the higher energy of the two to the tail of the lower-energy singlet transition means that the former is likely to be obscured by the latter. PtL^4^Cl provides perhaps the most convincing case. An expansion of the low-energy portion of the spectrum of this complex (Figure 4c) shows two shoulders—one at 500 nm and the other at 485 nm—which closely match those observed for the corresponding symmetric complexes PtL^4sym^Cl and Pt(dpyb)Cl and which may therefore be associated with the CF_3_-substituted pyridine and unsubstituted pyridine, respectively.

**Table 1:** Photophysical Data for the Pt(II) Complexes

complex	absorption at 298 K^a^	emission at 295 K^a^						emission at 77 K^b^	
	λ_max_/nm (ε/M^–^^1^ cm^–^^1^)	λ_max_/nm unimolecular {excimer}	Φ_lum_^c^	τ_0_/ns^d^	*k*_SQ_/10^9^ M^–^^1^ s^–^^1^^e^	*k*_r_/10^3^ s^–^^1^^e^	∑*k*_nr_/10^3^ s^–^^1^^e^	λ_max_/nm	τ/μs^f^
PtL^1^Cl	250 (36,600), 272 (37,400), 295 (30,800), 360 (9450), 375 (13,200), 405 (6180), 430 (2690), 490 (90)	495, 518, 558, 604 {700}	0.62	49,000 {870}	3.3	13	7.8	511, 526, 552, 598, 652	110
PtL^2^Cl	245 (36,400), 281 (28,800), 351 (8870), 366 (9680), 390 (7810), 405 (8650), 450 (4360), 538 (35), 582 (25)	594, 642, 696 {780}	0.11	3500 {580}	0.5	31	250	583, 633, 691, 763	5.8
PtL^3^Cl	240 (31,200), 268 (25,100), 290 (15,900), 335 (3850), 365 (4720), 384 (7720), 405 (6080), 484 (140)	487, 521, 556 {703}	0.59	7500 {480}	4.7	79	55	482, 498, 518, 552, 595	6.4
PtL^4^Cl	240 (31,900), 265 (21,200), 286 (19,600), 296 (18,100), 345 (5420), 395 (7980), 440sh (1560), 485 (150), 500 (120)	517, 548, 598 {738}	0.67	6100 [680]	3.7	110	54	508, 546, 585	6.0
PtL^5^Cl	242 (32,400), 266 (28,800), 290 (20,000), 320 (7680), 360 (6340), 379 (9060), 400 (7300), 483 (110)	487, 522, 556, 606sh {668}	0.66	5300 [410]	4.7	120	64	483, 500, 512sh, 522, 541, 553, 567, 599	6.7
PtL^6^Cl	240 (35,800), 263 (24,800), 287 (21,500), 337 (5600), 391 (9280), 430 (2450), 500 (100)	517, 550, 601 {733}	0.57	5400 [620]	3.4	110	80	508, 547, 585	5.9
PtL^6^*Cl	243 (55,300), 267 (25,900), 292 (23,700), 336 (6820), 395 (9810), 448 (2230), 511 (100)	538, 565sh {732}	0.77	7000 [610]	1.6	110	33	523, 563, 607	6.8
Pt(dpyb)Cl	239 (32,400), 257 (25,600), 290 (20,900), 332 (6510), 380 (8690), 401 (7010), 454 (270), 485 (240)	491, 524, 562 {696}	0.60	7200 [500]	5.3	83	56	484, 501, 512sh, 519, 556sh, 568	6.2
PtL^1sym^Cl	280 (69,900), 312sh (23,900), 362 (22,700), 409 (12,700), 490 (80)	519, 558, 603 {689}	0.40	83,000 [670]	2.1	4.8	7.3	512, 527, 554, 599, 653	130
PtL^2sym^Cl	285 (41,200), 333 (12,700), 363 (11,500), 397 (7800), 423 (11,500), 446sh (9800), 540 (100), 584 (80)	595, 642, 695 {801}	0.13	4300 [360]	1.9	30	200	586, 605, 634, 693, 761sh	5.8
PtL^3sym^Cl	237 (27,300), 268 (26,300), 365sh (4240), 383 (6850), 409 (5550), 479 (190)	483, 514, 552sh, 604sh {706}	0.83	6800 [560]	3.9	120	25	478, 493, 512, 550	5.5
PtL^4sym^Cl	238 (48,800), 268 (34,300), 284 (27,900), 348 (4690), 400 (11,400), 503 (200)	518, 548, 598 {756}	0.53	5000 [1000]	3.2	110	94	511, 549, 591	6.0
PtL^5sym^Cl	262 (32,400), 311sh (8900), 368 (8880), 403 (7380), 475 (150)	483, 516, 551 {642}	0.73	6800 [340]	3.2	110	40	475, 510, 544	6.0

a In CH_2_Cl_2_.

b In diethyl ether/isopentane/ethanol (2:2:1 v/v).

c Photoluminescence quantum yield in deoxygenated solution, measured using [Ru(bpy)_3_]Cl_2_ (aq) as the standard, for which Φ_lum_ = 0.04. The likely uncertainty on Φ_lum_ is around ±20%.

d In deoxygenated solution; corresponding values in parentheses refer to air-equilibrated solutions; λ_ex_ = 405 nm. Estimated uncertainty on τ is around ±10%.

e Radiative, *k*_r_, and nonradiative, ∑*k*_nr_, rate constants estimated assuming that the emitting state is formed with unit efficiency such that *k*_r_ = Φ_lum_/τ and ∑*k*_nr =_ (1 – Φ_lum_)/τ.

f λ_ex_ = 405 nm.

Similar overall conclusions are drawn for the complexes containing 1-isoquinoline or pyrimidine (Figure 4a). The 1-substituted isoquinoline complex PtL^2^Cl absorbs the longest wavelength—in line with its deeper red color compared to the yellow of the other complexes—with a band at around 450 nm. The corresponding symmetric PtL^2sym^Cl absorbs similarly in this region—where Pt(dpyb)Cl does not absorb—indicating that the lowest-energy band is associated with a transition to the isoquinoline unit. The molar absorptivity is, however, suppressed in this region in PtL^2^Cl compared to PtL^2sym^Cl by roughly 2-fold, consistent with there being only one isoquinoline in the molecule. Conversely, the absorption is enhanced around 400 nm, indicative of transitions involving the pyridyl ring at similar energies to those of Pt(dpyb)Cl. The spectrum of PtL^3^Cl is likewise very similar to PtL^3sym^Cl, although the latter is not very different from Pt(dpyb)Cl anyway. Indeed, in this case, the “mixed” composition of the nonsymmetric complex is clearer at higher energies: there is a distinct shoulder at 290 nm, absent from PtL^3sym^Cl, that matches a band at this wavelength in Pt(dpyb)Cl.

A possible exception to the conclusion that the two-halves of the *NCN* unit function essentially independently of one another is the case of PtL^1^Cl. Although its spectrum is similar in form to that of PtL^1sym^Cl, there is a definite shoulder at around 430 nm in the nonsymmetric complex that appears to have no counterpart in either PtL^1sym^Cl or Pt(dpyb)Cl. The intense band at 360 nm in PtL^1sym^Cl also appears to be significantly red-shifted in PtL^1^Cl by around 15 nm. These observations together suggest that the lowest spin-allowed excited states may involve more extended conjugation across all three aromatic units. Such a conclusion is reinforced by DFT calculations on PtL^1^Cl. The lowest-energy singlet transition has predominant HOMO → LUMO character. While the HOMO is based on the “central axis” of the molecule, just as for the other complexes (i.e., on the phenyl, metal, and halide), the LUMO is seen to be delocalized over all four rings (Figure S21). The calculated energy of 2.838 eV (equivalent to 437 nm) matches well with the lowest-energy band in the spectrum.

### 2.5 Emission Spectra at 77 K

**Figure 5:**
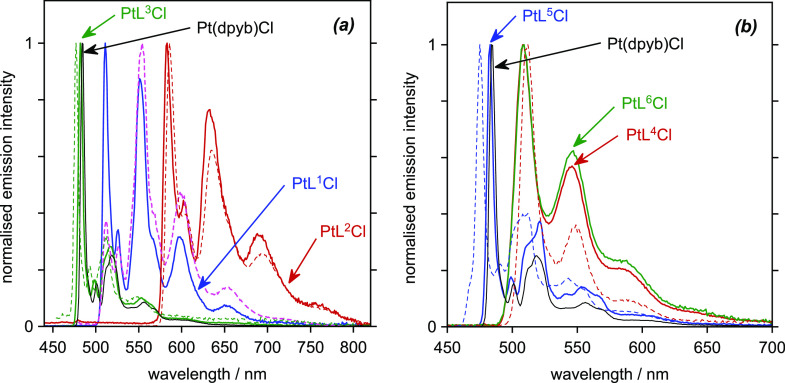
Emission spectra at 77 K in EPA: (a) 3-isoquinoline, 1-isoquinoline, and pyrimidine-containing complexes and their symmetric analogues and (b) para-substituted pyridine complexes and symmetric analogues; λ_ex_ = 420 nm in each case. Color coding as in Figure 4, except that PtL^1sym^Cl is shown in dotted pink to help distinguish it from the very similar PtL^1^Cl spectrum. Pt(dpyb)Cl is again shown as the black line in both plots (EPA = diethyl ether/isopentane/ethanol, 2:2:1 v/v).

We first consider the emission spectra recorded at 77 K in a dilute transparent glass of EPA (Figure 5; EPA = diethyl ether/isopentane/ethanol, 2:2:1 v/v). Under these conditions, the complexes display highly resolved vibrational structure in the phosphorescence spectra, typical of previously reported Pt(*NCN*)Cl complexes,^10,17^ which allow unequivocal comparisons to be made between their excited-state energies, as estimated from the 0,0 components. A previous work on symmetrical complexes showed that *para*-CF_3_ substituents in the pyridine rings red-shift the emission while *para*-MeO substituents lead to a blue shift, due to their electron-withdrawing/donating influence in lowering/increasing the energy of π* orbitals, respectively. Thus, the 0,0 band of PtL^4sym^Cl appears around 1500 cm^–1^ to lower energy of PtL^5sym^Cl (Figure 5b). It can be seen clearly that the two nonsymmetric complexes containing CF_3_ substituents in just one of the two pyridine rings—PtL^4^Cl and PtL^5^Cl—display emission spectra that are very similar to that of the bis-CF_3_ complex PtL^4sym^Cl. They are substantially red-shifted compared to Pt(dpy)Cl and PtL^5sym^Cl. Clearly, then, the emission arises from a state involving the CF_3_-substituted pyridine with its lower-energy π* orbitals. Similarly, the spectrum of the MeO-containing PtL^5^Cl more closely matches that of Pt(dpyb)Cl than PtL^5sym^Cl, suggesting that the pertinent excited state involves the unsubstituted pyridine ring as opposed to the more electron-rich methoxypyridine. The same trend is equally apparent from the spectra of PtL^1^Cl and PtL^2^Cl, which closely match those of PtL^1sym^Cl and PtL^2sym^Cl, respectively, rather than the spectrum of Pt(dpyb)Cl. Evidently, the emissive excited state involves the isoquinoline unit rather than the pyridine unit. The 0,0 energy of PtL^3sym^Cl is very similar to that of Pt(dpyb)Cl and not surprisingly therefore to that of PtL^3^Cl too.

These conclusions from the emission spectra thus match those about the lowest-energy singlet state from the absorption spectroscopy, namely that the lowest-energy triplet excited state involves essentially only one-half of the *NCN* unit, that of the heterocycle with lowest-energy π* orbitals. With one exception, the phosphorescence lifetimes are around 6 μs in all cases (Table 1), similar to that of Pt(dpyb)Cl and consistent with a triplet ^3^[d_M_|π_N′CN″_ → π_N′CN″_*] excited state with sufficient metal character to efficiently promote the formally forbidden T_1_ → S_0_ transition. The striking exception is the 3-substituted isoquinoline complex PtL^1^Cl, which has a much longer lifetime of 110 μs under these conditions. PtL^1sym^Cl was similarly found to have an anomalously long lifetime of 109 μs at 77 K.^16^ Presumably, the different conjugation pattern in this complex compared to the isomeric PtL^2^Cl/PtL^2sym^Cl leads to a smaller contribution of metal character to the excited state.

### 2.6 Emission Spectra at 295 K in Dilute Solutions

**Figure 6:**
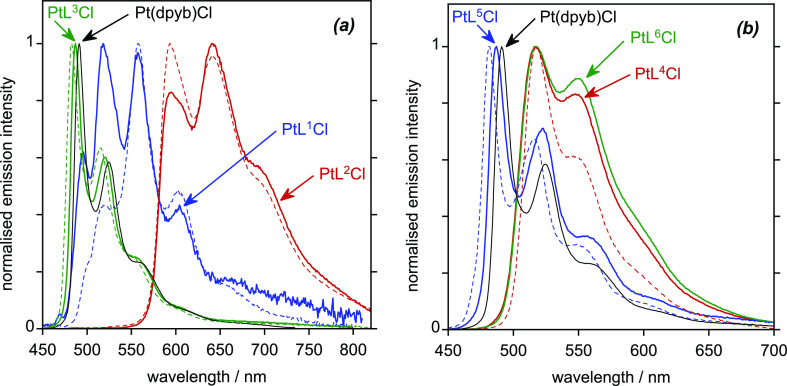
Emission spectra at 295 K in CH_2_Cl_2_ (a) 3-isoquinoline, 1-isoquinoline, and pyrimidine-containing complexes and their symmetric analogues and (b) para-substituted pyridine complexes and symmetric analogues PtL^4sym^Cl and PtL^5sym^Cl; λ_ex_ = 420 nm. Concentration = 2 × 10^–5^ M, except for PtL^1^Cl and PtL^1sym^Cl which are shown here at 2 × 10^–6^ M to avoid excimer formation, see Section 2.7. Color coding as in Figure 4, with Pt(dpyb)Cl shown for reference.

Comparable conclusions may be drawn—at least for PtL^2^Cl, PtL^4^Cl, and PtL^6^Cl—from the emission spectra at room temperature in dichloromethane solution, Figure 6. The spectra are similar to those at 77 K, again showing clear vibrational structure in each case, albeit with slightly broader bands. Again, the CF_3_-appended complexes PtL^4^Cl and PtL^6^Cl resemble PtL^4sym^Cl, while PtL^2^Cl resembles PtL^2sym^Cl. The only notable difference is an enhancement of the 0,1 and 0,2 vibrational components relative to the 0,0 band for the nonsymmetric complexes compared to the symmetric parents. This might suggest a slightly more distorted excited-state geometry, though the difference is small.

For the higher-energy pyrimidine-containing PtL^3^Cl and MeO-substituted complex PtL^5^Cl, the spectra and λ_max_ values fall between those of the two corresponding parent complexes [PtL^3sym^Cl/Pt(dypb)Cl and PtL^5sym^Cl/Pt(dpyb)Cl, respectively]. This could be indicative of thermal equilibration between the two sets of excited states associated with the two different heterocycles, at room temperature, in contrast to the spectra at 77 K where emission appeared to emanate only from the state associated with the heterocycle of lower π* energy (i.e., the unsubstituted pyridine). Based on the λ(0,0) values of the symmetric complexes, the energy difference between the triplet states associated with the two heterocycles of PtL^3^Cl is only about 300 cm^–1^ and for PtL^5^Cl around 400 cm^–1^ (cf. *k*_B_*T* at 295 K = 205 cm^–1^). For PtL^2^Cl, PtL^4^Cl, and PtL^6^Cl, the corresponding differences are much larger (3600, 1100, and 1400 cm^–1^) and such an equilibration between the states would thus not be expected at 295 K, in line with the observations.

**Figure 7:**
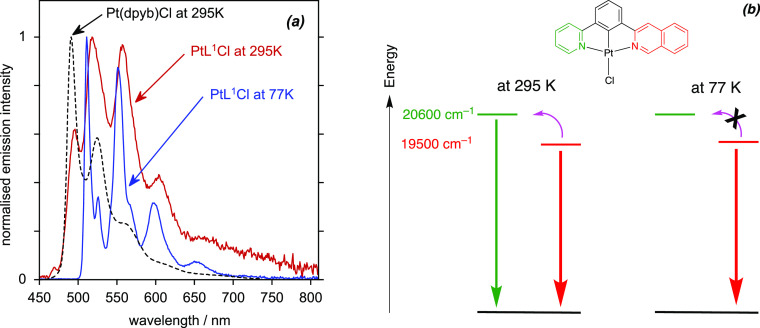
(a) Emission spectrum of PtL^1^Cl at 295 K compared to 77 K (red and blue lines of CH_2_Cl_2_ and EPA, respectively) together with that of Pt(dpyb)Cl at 295 K for comparison (black); λ_ex_ = 420 nm. Note how PtL^1^Cl shows bands similar to Pt(dpyb)Cl only at the higher temperature. (b) Schematic diagram (not to scale) showing the proposed repopulation (pink arrow) of the higher-energy pyridine-based state from the isoquinoline-based state, which is suppressed at 77 K.

The 3-isoquinoline complex PtL^1^Cl is particularly interesting in this regard. It can be seen that the spectrum of this complex at 295 K features bands that closely match those of PtL^1sym^Cl, but there is now an additional band at higher energy (around 495 nm) that is absent from PtL^1sym^Cl and which is not present at 77 K (Figure 7a), while the band at 518 nm is strongly enhanced compared to PtL^1sym^Cl. Indeed, the spectrum is similar to what might be anticipated if emission emanates simultaneously from two different excited states associated with the different heterocycles, i.e., ^3^[d_Pt_ |π_pyCquin_ → π_pyCquin_*] and ^3^[d_Pt_ |π_pyCquin_ → π_pyCquin_*]. A spectrum simulated simply from the average of the spectra of the two parent complexes PtL^1sym^Cl and Pt(dpyb)Cl shows a remarkable resemblance to the experimental room-temperature spectrum (Figure S10). If we use the λ(0,0) bands at 77 K to estimate the triplet energies of Pt(dpyb)Cl and PtL^1sym^Cl, then the energy gap between the two states can be estimated to be around 1000 cm^–1^. Although this is substantially larger than that in PtL^3^Cl and PtL^5^Cl above, there is an important difference to note, namely the very long luminescence lifetime associated with PtL^1sym^Cl, of 83 μs. Such a long lifetime associated with the ^3^[d_Pt_ |π_pyCquin_ → π_pyCquin_*] state evidently ensures sufficient time for thermal activation to a state around 1000 cm^–1^ higher to the ^3^[d_Pt_ |π_pyCquin_ → π_pyCquin_*]. The distinction in behavior between the two temperatures is summarized in Figure 7b.

DFT calculations for the triplet states at the optimized T_1_ geometry support the interpretation. They show that the first triplet excited state is mainly HOMO → LUMO in character, with LUMO being based largely on the isoquinoline ring, while the predominant transition contributing to T_2_ is HOMO → LUMO + 2, where LUMO + 2 is mostly based on the pyridine ring (Figures S22 and S23).

The observed dual emission at 295 K could alternatively be accounted for solely in terms of the energy of the absorbed light being channeled independently into either of the triplet excited states, which then emit, without invoking thermal activation from the lower-energy, longer-lived emissive state to the higher energy one. However, in that case, the profile of the emission spectrum (in particular, the ratio of the intensities at 495 vs 550 nm) would be expected to vary with the excitation wavelength. On the contrary, the spectral profile is independent of the excitation wavelength selected. Moreover, the excitation spectrum registered at the high-energy band (495 nm) is identical to the excitation spectra registered at longer wavelengths (Figure S11). Neither of these observations seems consistent with the notion of independent channels. Moreover, the temporal decay of the luminescence at 495 nm was found to be the same as that at longer wavelengths (within the uncertainty on the measurement). If the ^3^[d_Pt_ |π_pyCquin_ → π_pyCquin_*] state is populated by thermal activation from from the long-lived, lower-energy ^3^[d_Pt_ |π_pyCquin_ → π_pyCquin_*], then its observed lifetime will primarily reflect the lifetime of the latter. A lifetime of 49 μs was determined (at infinite dilution, vide Section 2.6), somewhat lower than that of Pt^1sym^Cl,^16^ consistent with an additional deactivation pathway at play through the pyridyl-based excited state.

The lifetimes of the other six nonsymmetric complexes were comparable with those of their symmetric analogues. It can be seen (Table 1) that for most complexes (both nonsymmetric and symmetric), the lifetimes at room temperature are similar to those at 77 K. In a few cases, the lifetimes appear to be slightly shorter than at room temperature, but the values are within the uncertainty on the measurements and caution should be applied in seeking any interpretation. Longer lifetimes are generally to be anticipated at 77 K, reflecting the expected suppression of the nonradiative decay process that involve distortion and significant motion. In these complexes, however, the quantum yields are very high even at room temperature, indicating that such decay pathways are already inefficient compared to radiative decay, so the effect of decreasing temperature on τ may be minimal. It is also possible that the relative energies of higher-lying singlet excited states (with which the emissive triplet states must mix through spin–orbit coupling^4^) may differ between the two sets of conditions, potentially leading to somewhat different values for the radiative rate constant *k*_r_.

The quantum yields Φ_lum_ in deoxygenated solution are all very high at around 0.6, similar to Pt(dpyb)Cl, except for PtL^2^Cl which is a weaker emitter, Φ_lum_ = 0.11. The emission of this complex is significantly red-shifted compared to the others, and one might therefore anticipate that its emission efficiency will be compromised by increased nonradiative decay. Indeed, inspection of the radiative *k*_r_ and nonradiative Σ*k*_nr_ rate constants in Table 1 (estimated from the lifetimes and quantum yields as indicated in the table footnote) confirms that Σ*k*_nr_ for this complex is significantly larger than for the others. The data also reveal that the emission efficiency of the 1/3-isoquinoline-based complexes PtL^1,2^Cl is also compromised by lower radiative rate constants *k*_r_, just as it is for their symmetric analogues. Such a trend is to be anticipated since the degree of d_Pt_ participation in the excited state will typically fall as the extent of conjugation in the ligand increases, as highlighted elsewhere.^28^ The very long lifetime of the 3-isoquinoline complexes PtL^1^Cl and Pt^1sym^Cl stems from the combination of especially low *k*_r_ and Σ*k*_nr_ values.

### 2.7 Emission at Elevated Concentrations in Solution: Excimer Formation

As noted in the introduction, Pt(dpyb)Cl and many of its derivatives efficiently form excimers in solution, as the concentration is increased, which may in turn emit in the red/NIR region of the spectrum. The depletion of the unimolecular excited state through the formation of excimers leads to a decrease in the lifetime of the former with increasing concentration. All the new complexes studied here show concentration-dependent self-quenching accompanied by excimer formation. Plots of the observed monoexponential emission decay constant *k*_obs_ as a function of concentrations are generally linear (Figure S18), consistent with a dynamic Stern–Volmer process, except at the very highest concentrations where some small deviations are encountered that may be due to ground-state aggregate formation, as we described previously for Pt(dpyb)Cl.^6c^ The Stern–Volmer quenching constants and lifetimes extrapolated to infinite dilution are compiled in Table 1.

For each complex, the excitation spectra recorded for the low-energy band that appears at higher concentrations are essentially identical to the excitation spectrum of the unimolecular emission (Figure S19). This observation, as for Pt(dpyb)Cl itself,^6c,10^ supports the assignment of the low-energy emission to excimers formed via the unimolecular excited state, as opposed to aggregates (e.g., dimers, trimers, or higher oligomers) pre-existing in the ground state. The latter species typically display additional absorption band(s) at low energy; e.g., corresponding to MMLCT transitions,^6c^ which would lead to differences in the excitation spectra compared to those of the isolated molecules. Further evidence in support of the excimer formulation comes from the time-resolved data (see Figure S20). The low-energy emission band shows an initial “grow-in” phase, during which the intensity increases, reflecting the time taken for the excimers to form. After this growth phase, the emission decays following monoexponential kinetics, with a time constant that is the same (within the uncertainty on the measurement) as that of the unimolecular band. Such a situation is expected according to Birks’ classic kinetic model of excimers^29^ when the natural rate constant of decay of the excimer substantially exceeds that of the monomer, such that the observed rate of decay of both species follows that of the monomer. The kinetic model as applied to Pt(dpyb)Cl and symmetric derivatives has been discussed recently elsewhere.^30,6c^

**Figure 8:**
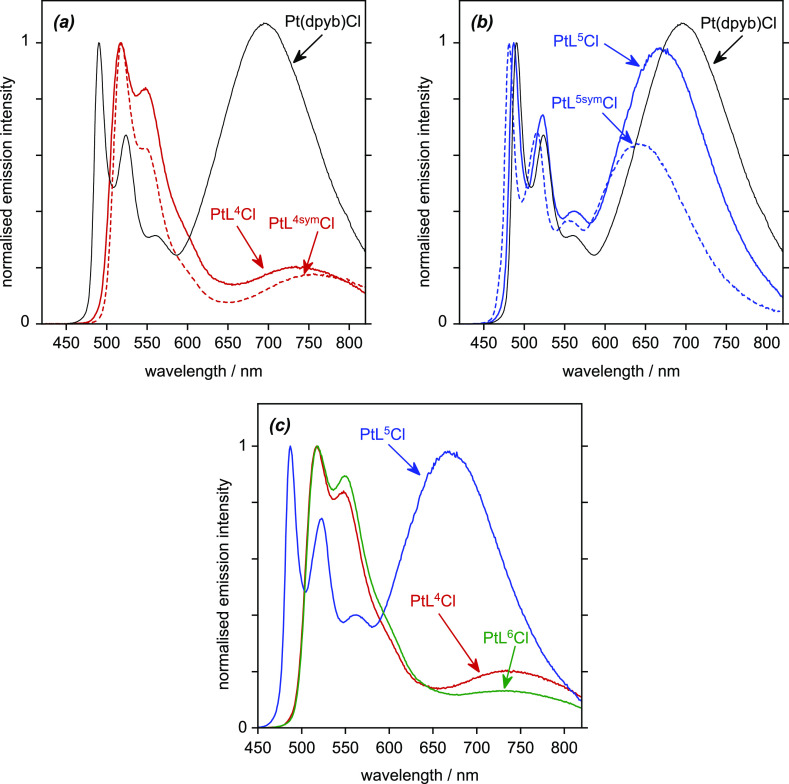
Emission spectra (λ_ex_ = 420 nm) of concentrated solutions (2 × 10^–4^ M) of the complexes in CH_2_Cl_2_ at 295 K to highlight trends in λ_max_ of the excimer: (a) CF_3_-substituted PtL^4^Cl and its symmetric analogues; (b) OMe-substituted PtL^5^Cl and its symmetric analogues; and (c) comparison of PtL^6^Cl, the “mixed” MeO/CF_3_ complex, with PtL^4^Cl and PtL^5^Cl.

We first consider the trends among the bis-pyridyl systems PtL^4–6^Cl and relative to their symmetric analogues (Figure 8). Previously, we reported that the symmetric CF_3_-substituted complex PtL^4sym^Cl displayed a significantly red-shifted excimer relative to the parent complex Pt(dpyb)Cl but with lower intensity.^15^ It can be seen that PtL^4^Cl shows this same trend, with only a weak excimer band (Figure 8a). The λ_max_ of the broad band is at about 738 nm compared to 756 and 696 nm for PtL^4sym^Cl and Pt(dpyb)Cl. This intermediate value suggests the presence of a mixture of homo- and heteropyridyl excimer species. The methoxy-substituted system PtL^5^Cl shows a blue shift in the excimer to λ_max_ = 669 nm, in line with the behavior of the bis-methoxy PtL^5sym^Cl (for which λ_max_ = 642 nm). Again, λ_max_ is an intermediate between those of the corresponding symmetric systems (Figure 8b). Finally, it can be seen that the “mixed” MeO/CF_3_ complex PtL^6^Cl behaves more like the CF_3_-substituted PtL^4^Cl than the MeO-substituted PtL^5^Cl, with a weak excimer band centered around 733 nm (Figure 8c). Emission evidently emanates from the lowest-energy excimer state involving the CF_3_-substituted pyridine. However, there is no evidence of a more stable excimeric excited state for the mixed complex compared to PtL^4^Cl, despite the donor–acceptor complementarity that might have been envisaged between the MeO-substituted pyridine of one constituent molecule of the excimer and the CF_3_-substituted pyridine of the other.

For each of these nonsymmetric complexes, the Stern–Volmer quenching constants and lifetimes at infinite dilution are similar to those of their symmetric counterparts, indicating that the propensity to bimolecular interactions and excimer formation is not significantly different, as is also evident from the relative contribution of the excimer to the spectra. The presence of the *t*-butyl group in the phenyl ring of PtL^6^*Cl does appear to attenuate excimer formation to some extent, as is evident from the smaller *k*_SQ_ of this complex (a factor of 1/2) compared to PtL^6^Cl. This is also manifest from the spectra at elevated concentrations, the contribution of the excimer to the spectrum being even smaller for PtL^6^*Cl than PtL^6^Cl (Figure S12). It is notable, however, that the emission energy of the excimer is essentially unchanged, with λ_max_ around 732 mm for both (Figures 8c, S12, and S13). Evidently, although the relatively bulky *t*-butyl group may hinder the approach of molecules to one another, it does not inhibit the attainment of the energy minimum in the excimeric state. Similar conclusions have previously been made for symmetric Pt(dpyb)Cl derivatives with various substituents in the same position.^10b^

The pyrimidine complex PtL^3^Cl gives an intense excimer band centered at around 703 nm, very similar to the symmetric analogue PtL^3sym^Cl (Figure S14). Indeed, the spectra for these two complexes are essentially identical, and like that of Pt(dpyb)Cl, just as was found in dilute solution. The Stern–Volmer quenching constants of all three complexes are also very similar to one another. The change from pyridine to pyrimidine does not significantly influence the emission properties in solution, though we note that in recent studies of neat films of PtL^3sym^*Cl, the pyrimidine rings were found to lead to ground-state aggregates that emit at even lower energy.^7d^

The 1-isoquinoline complex PtL^2^Cl shows only a hint of enhanced low-energy emission in saturated solution compared to dilute conditions. Attempted deconvolution of the excimer from the spectrum reveals a λ_max_ of around 780 nm (Figure S15). The Stern–Volmer quenching constant is smaller for this complex than the others, which may partly explain the low intensity of the excimer. Recall also that the unimolecular emission of this complex is already significantly lower in energy than the others, such that the excimer emission appears superimposed on the low-energy side of the unimolecular bands, as we found previously for PtL^2sym^Cl for which the excimer λ_max_ = 800 nm.^6c^ The small blue shift of the excimer in PtL^2^Cl relative to the symmetric complex could indicate some involvement of “Pt(dpyb)Cl-like” excimers (as in PtL^4^Cl above).

**Figure 9:**
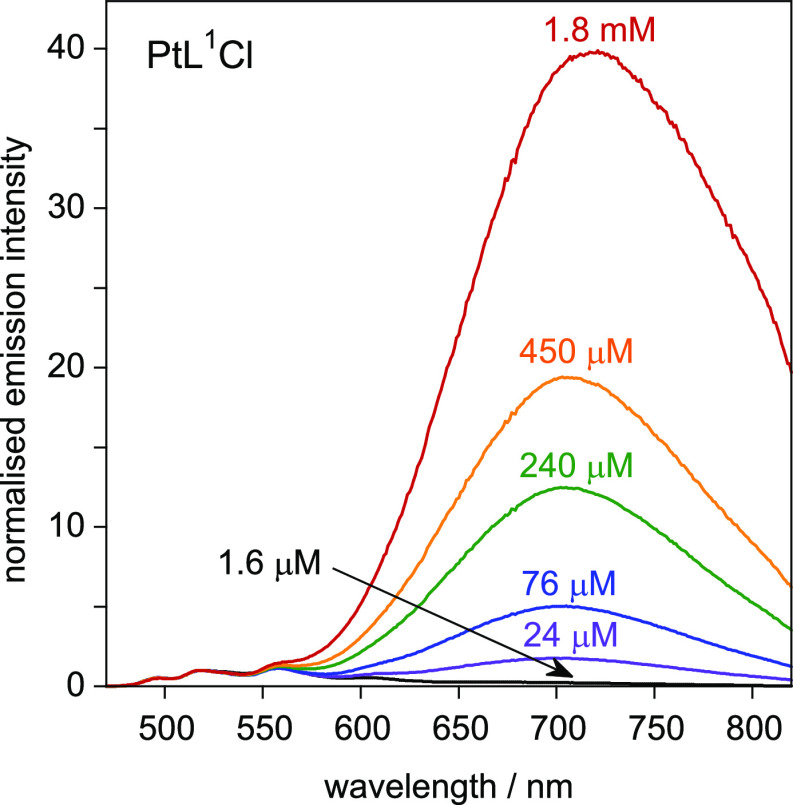
Emission spectra of PtL^1^Cl at 295 K in CH_2_Cl_2_ at the concentrations indicated, normalized to intensity = 1 at 518 nm; λ_ex_ = 420 nm. The bands between 495 and 605 nm are the unimolecular emission bands; cf. the spectra of very dilute solutions in Figure 6a.

The 3-isoquinoline complexes PtL^1^Cl and PtL^1sym^Cl are especially interesting. Excimer emission emerges at much lower concentrations than all the other complexes including Pt(dpyb)Cl, being apparent even at around 10^–6^ M. As the concentration increases, the excimer ends up completely dominating the spectrum, with residual unimolecular emission almost lost in the baseline (Figure 9). The *k*_SQ_ values are no larger for these two complexes than the others (Table 1). Recall, however, that they differ in the anomalously long lifetimes of their unimolecular excited states [Section 2.6 above].^31^ A long unimolecular lifetime favors excimer formation as it allows more time for an excited-state molecule to encounter a ground-state partner under diffusion control. Nevertheless, the remarkable intensity of the excimers of these complexes can probably not be accounted for solely on the grounds of the modestly longer unimolecular lifetimes: it is likely that the excimers of PtL^1^Cl and PtL^1^*Cl are intrinsically more emissive than the others. Given that the ∑*k*_nr_ values of their unimolecular excited states are around an order of magnitude lower than for the other complexes, it may be that the corresponding excimeric states are, likewise, less subject to nonradiative decay, leading to more intense emission. The excimer band of the nonsymmetric complex is a little red-shifted (about 10 nm) relative to its symmetric parent PtL^1sym^Cl, while the relative contribution of excimer appears somewhat lower for a given concentration (e.g., Figure S16, showing spectra at 24 μM). However, this probably just reflects the contribution of competing emission from the higher-energy unimolecular emissive state involving the pyridine as opposed to the isoquinoline ring in PtL^1^Cl (as discussed for the unimolecular emission in Section 2.6). The small but definitive shift of λ_max_ of the red band to longer wavelengths at the highest concentrations (more readily seen in the intensity-normalized spectra of Figure S16) is consistent with recent observations of related systems and is attributed to emission from higher-order species involving >2 molecular units.^6c,7d^

## 3 Concluding Remarks

Tridentate *NCN*-coordinating ligands based on 2,6-dipyridylbenzene continue to furnish platinum(II) complexes with outstanding luminescence properties, including high emission quantum yields and a propensity to form excimers and aggregates that emit efficiently, deep into the red and NIR regions of the spectrum. These attractive properties are found, in this study, to be retained or enhanced in complexes of nonsymmetric ligands that feature two different heterocycles. Emission is dominated by triplet excited states involving the heterocycle with the lowest-energy π* orbitals, although thermal activation to the corresponding state involving the other heterocycle may occur. Such behavior could prove to be of interest to ratiometric luminescence thermometry.^32^ There is no clear evidence of a complementary donor–acceptor-type structure leading to displacement of the bimolecular excited states to lower energies, although all the new complexes do emit into the NIR at elevated concentrations, and they may prove fertile ground for the development of emitters for NIR OLEDs.

## 4 Experimental Details

### 4.1 General

Reagents were obtained from commercial sources and used without further purification unless stated otherwise. All solvents used in preparative work were at least of Analar grade and water was purified using the PuriteSTILL plus system. Dry solvents were obtained from HPLC grade solvent that had been passed through a Pure Solv 400 solvent purification system and stored over activated 3 or 4 Å molecular sieves. For procedures involving dry solvent, glassware was oven-dried at 110 °C prior to use. Reactions requiring an inert atmosphere were carried out using Schlenk-line techniques under an atmosphere of nitrogen. Thin-layer chromatography was carried out using silica plates (MerckArt 5554) and visualized by UV radiation at 254 and/or 365 nm. NMR spectra were recorded on a Bruker Avance-400 spectrometer. Two-dimensional spectra used to aid assignments (COSY, NOESY, HSQC, and HMBC) were acquired on a Varian VNMRS-600 (600 MHz) or Varian VNMRS-700 (700 MHz) instrument. Chemical shifts (δ) are in ppm, referenced to residual protio solvent resonances, and coupling constants are given in hertz. Electrospray ionization mass spectral data (positive and negative modes) were obtained on an SQD mass spectrometer interfaced with an Acquity UPLC system with acetonitrile as the carrier solvent. Spectra acquired using an atmospheric solids atomization probe were recorded on a Waters Xevo QToF mass spectrometer.

### 4.2 Synthetic Procedures and Characterization

The syntheses of HL^1^ and HL^6^*, together with their Pt(II) complexes, are provided below as representative examples. The detailed synthesis and characterization of all the other proligands and complexes are provided in the Supporting Information.

#### 4.2.1 2-[3-(4,4,5,5-Tetramethyl-1,3,2-dioxaborolan-2-yl)phenyl]-pyridine: ppy-Bpin

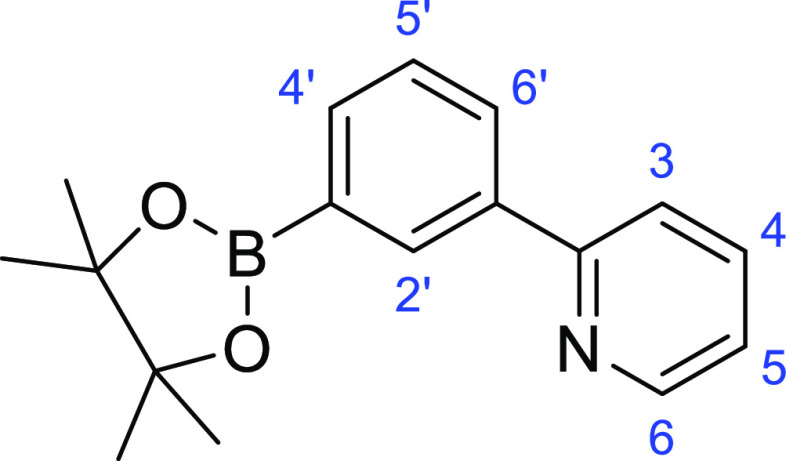



This key intermediate (see Scheme 1) was prepared by Pd-catalyzed cross-coupling of **ppy-Br** (prepared as described in the Supporting Information; 111 mg, 0.47 mmol) with bis-pinacolato diboron (145 mg, 0.57 mmol). A mixture of these two reagents and KOAc (140 mg, 1.42 mmol) in 1,4-dioxane (5 mL) in a Schlenk tube was degassed by three freeze–pump–thaw cycles and the vessel was backfilled with nitrogen gas. The catalyst, Pd(dppf)Cl_2_ (17 mg, 0.02 mmol), was added under a flow of nitrogen and the mixture was heated at 80 °C for 18 h. The solvent was then removed under reduced pressure and the residue extracted into CH_2_Cl_2_, with insoluble material being filtered off. After removal of solvent, the crude, darkly colored solid was purified by column chromatography on silica (gradient elution from CH_2_Cl_2_ to CH_2_Cl_2_: MeOH, 70:30) to yield the product as a brown oily solid (55 mg, 41%). ^1^H NMR (700 MHz, CDCl_3_): δ_H_ 8.65 (1H, ddd, *J* 4.9, 1.8, 0.9, H^6^), 8.33 (1H, s, H^2′^), 8.07 (1H, ddd, *J* 7.8, 2.0, 1.3, H^6′^), 7.83 (1H, dt, *J* 7.3, 1.2, H^4′^), 7.76 (1H, dt, *J* 8.0, 1.2, H^3^), 7.73 (1H, td, *J* 7.7, 1.8, H^4^), 7.48–7.45 (1H, m, H^5′^), 7.21 (1H, ddd, *J* 7.3, 4.9, 1.3, H^5^), 1.34 (12H, s, H^Bpin^). ^13^C (176 MHz, CDCl_3_) 157.5 (C^2^), 149.5 (C^6^), 138.6 (C^1′^ or C^3′^), 136.8 (C^4^), 135.3 (C^4′^), 133.2 (C^2′^), 131.1 (C^1′^ or C^3′^), 129.9 (C^6′^), 128.2 (C^5′^), 122.1 (C^5^), 120.9 (C^3^), 83.9 (C^Bpin^), 24.6 (C^Me–Bpin^). MS ESI (ES^+^) *m*/*z* 282.2 ([M + H]^+^, 100%); HRMS (ES^+^) *m*/*z* 281.1705 [M(^10^B) + H]^+^, calcd for [C_17_H_21_NO_2_^10^B]^+^, 281.1702.

#### 4.2.2 1-(3-Isoquinolyl)-3-(2-pyridyl)benzene: HL^1^

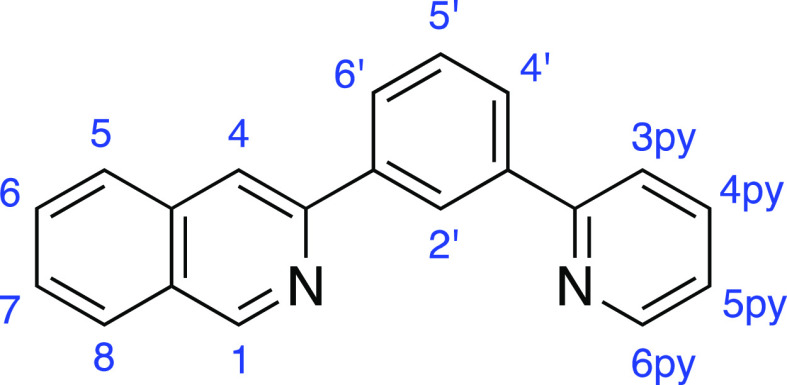



This proligand was prepared by Pd-catalyzed Suzuki cross-coupling of 3-isoquinolyl triflate (284 mg, 1.02 mmol) with **ppy-Bpin** (240 mg, 0.85 mmol). A mixture of these two reagents in dimethoxyethane (7 mL), with aqueous Na_2_CO_3_ (724 mg, 6.83 mmol, in 7 mL water), in a Schlenk was degassed by three freeze–pump–thaw cycles and backfilled with nitrogen gas. The catalyst Pd(PPh_3_)_4_ (49 mg, 0.04 mmol) was then added under a flow of nitrogen and the mixture was heated at 85 °C for 18 h. Water (10 mL) was added and the mixture extracted into CH_2_Cl_2_ (3 × 15 mL). The organic extracts were combined and dried over anhydrous MgSO_4_, and the solvent then removed under reduced pressure. The resulting residue was purified by column chromatography on silica (gradient elution from hexane to hexane/ethyl acetate, 80:20) to yield the desired compound as a clear oil (96 mg, 44%). ^1^H NMR (600 MHz, CDCl_3_): δ_H_ 9.42 (1H, s, H^1^), 8.78 (1H, ddd, *J* 4.9, 1.8, 1.0, H^6py^), 8.74 (1H, dt, *J* 1.9, 1.0, H^2′^), 8.26 (1H, s, H^4^), 8.20 (1H, ddd, *J* 7.8, 1.9, 1.1, H^4′^ or H^6′^), 8.08–8.01 (2H, m, H^4′^ or H^6′^, and H^5^), 7.92–7.96 (2H, m, H^8^ and H^3py^), 7.87 (1H, td, *J* 7.7, 1.8, H^4py^), 7.76 (1H, ddd, *J* 8.2, 6.8, 1.2, H^7^), 7.62–7.66 (2H, m, H^6^ and H^5′^), 7.33 (1H, ddd, *J* 7.4, 4.9, 1.2, H^5py^). ^13^C NMR (151 MHz, CDCl_3_): δ_C_ 210.8 (C^q^), 156.8 (C^q^), 151.8 (C^1^), 149.9 (C^q^), 148.9 (C^6py^), 139.1 (C^q^), 139.0 (C^q^), 137.8 (C^4py^), 137.0 (C^q^), 131.4 (C^7^), 129.6 (C^6^ or C^5′^), 128.1 (C^4′^ or C^6′^), 127.9 (C^4′^, and C^6′^ or C^5^), 127.7 (C^6^ or C^5′^), 127.5 (C^4′^ and C^6′^ or C^5^), 127.1 (C^8^ or C^3py^), 125.9 (C^2′^), 122.6 (C^5py^), 121.4 (C^8^ or C^3py^), 117.7 (C^4^). MS ESI (ES^+^) *m*/*z* 283.2 ([M + H]^+^, 100%); HRMS (ES^+^) *m*/*z* 283.1240 [M + H]^+^, calcd for [C_20_H_15_N_2_], 283.1235.

#### 4.2.3 PtL^1^Cl

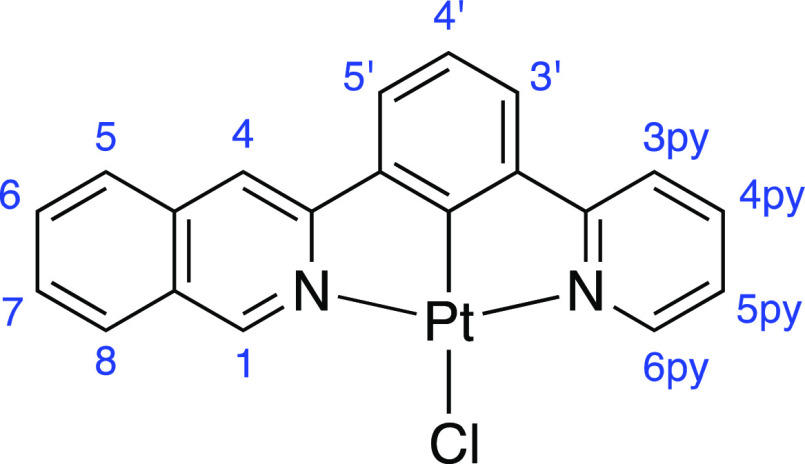



Potassium tetrachloroplatinate(II) (84 mg, 0.20 mmol) was added to a solution of HL^1^ (50 mg, 0.18 mmol) in acetic acid (5 mL) in a Schlenk tube and the solution was degassed by three freeze–pump–thaw cycles. The reaction mixture was then heated at reflux under a nitrogen atmosphere for 60 h. Upon cooling to room temperature, water was added (5 mL). The resulting precipitate was separated by centrifugation and washed successively with water, methanol, and diethyl ether. The solid was extracted into CH_2_Cl_2_ (3 × 5 mL) and the solvent was then removed under reduced pressure to give the desired complex as a yellow solid (52 mg, 57%). ^1^H NMR (700 MHz, CDCl_3_): δ_H_ 10.06 (1H, s, H^1^), 9.39 (1H, d, *J* 5.5, H^6py^), 8.09 (1H, d, *J* 7.6, H^5^), 7.99 (1H, s, H^4^), 7.96–7.91 (1H, m, H^4py^), 7.89 (1H, d, *J* 8.2, H^8^), 7.85–7.80 (1H, m, H^7^), 7.69 (1H, d, *J* 7.6, H^3py^), 7.65 (1H, t, *J* 7.6, H^6^), 7.54 (1H, d, *J* 7.6, H^3′^ or H^5′^), 7.43 (1H, d, *J* 7.6, H^3′^ or H^5′^), 7.30–7.28 (1H, m, H^5py^), 7.26 (1H, d, *J* 7.6, H^4′^). MS ASAP^+^*m*/*z* 517.1 ([M-Cl + MeCN]^+^, 100%). HRMS (ASAP^+^) *m*/*z* 516.0972 [M-Cl + MeCN]^+^, calcd for [C_22_H_16_N_3_^194^Pt] 516.0971. Anal. Calcd for C_20_H_13_ClN_2_Pt·0.1CH_2_Cl_2_: C, 46.39; H, 2.56; N, 5.38%. Found: C, 46.18; H, 2.61; N, 5.32%.

#### 4.2.4 2-(3-Bromo-5-*tert*-butyl-phenyl)-4-trifluoromethylpyridine

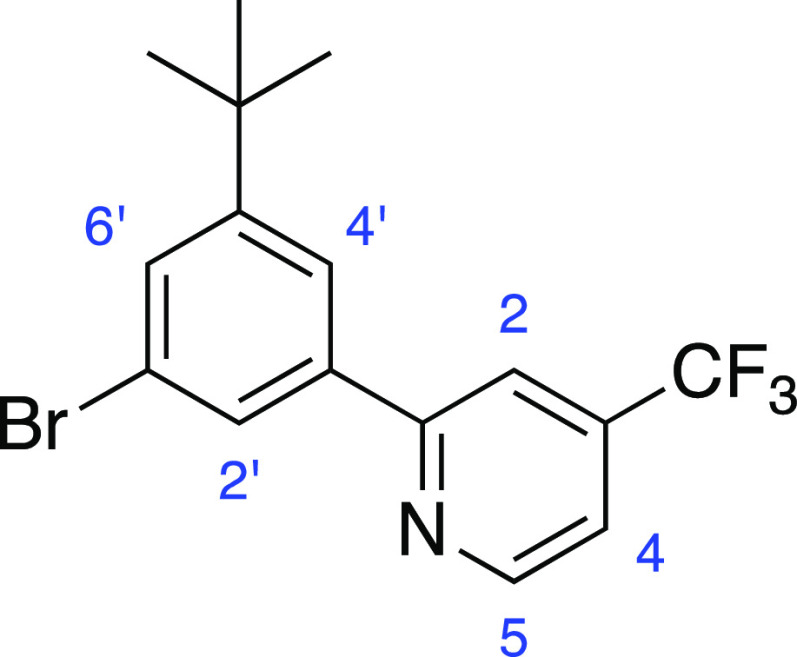



This compound was prepared by Pd-catalyzed Suzuki cross-coupling of 3-(4,4,5,5-tetramethyl-1,3,2-dioxaborolan-2-yl)-5-*tert*-butyl-bromobenzene (1.16 g, 3.43 mmol) with 2-bromo-4-(trifluoromethyl)pyridine (0.968 g, 4.28 mmol), aqueous Na_2_CO_3_ (2.90 g, 27.4 mmol), Pd(PPh_3_)_4_ (0.198 g, 0.171 mmol), and DME (20 mL). The procedure was otherwise as described for HL^1^ above. The crude product was purified by column chromatography on silica (hexane: ethyl acetate gradient, *R*_f_ = 0.5 in 90:10) to yield the pure product as a white solid (429 mg, 35%). ^1^H NMR (400 MHz, CDCl_3_): δ_H_ 8.93–8.87 (1H, m, H^5^), 8.00 (2H, dt, *J* 9.2, 1.7, H^4′^ and H^6′^), 7.90 (1H, dt, *J* 1.6, 0.8, H^2^), 7.65 (1H, t, *J* 1.7, H^2′^), 7.53–7.47 (1H, m. H^4^). MS ESI (ES^+^): *m*/*z* 358.1 ([M + H]^+^, 93%); HRMS (ES^+^): *m*/*z* 358.0434 [M + H]^+^, calcd for 358.0418 [C_16_H_16_NF_3_Br].

#### 4.2.5 2-[3-(4,4,5,5-Tetramethyl-1,3,2-dioxaborolan-2-yl)-5-*tert*-butyl-phenyl]-4-trifluoromethyl-pyridine

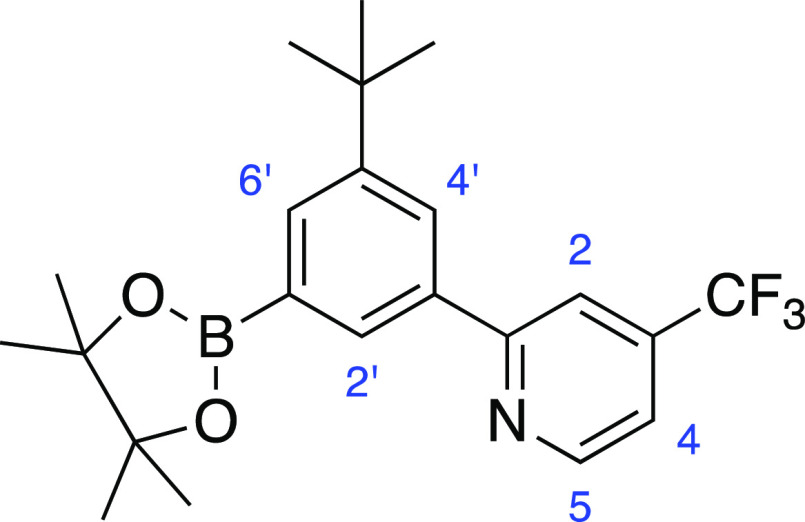



This compound was prepared by Pd-catalyzed borylation of 2-(3-bromo-5-*tert*-butyl-phenyl)-4-trifluoromethylpyridine (372 mg, 1.04 mmol), using the procedure described for **ppy-Bpin** above, with bis-pinacolatodiboron (318 mg, 1.25 mmol), KOAc (612 mg, 6.23 mmol), and PdCl_2_(dppf) (76 mg, 0.104 mmol) in 1,4-dioxane (10 mL). The crude product was purified by column chromatography on silica (hexane: ethyl acetate gradient, *R*_f_ = 0.4 in 90:10) to give the title compound as a white solid (283 mg, 67%). ^1^H NMR (600 MHz, CDCl_3_): δ_H_ 8.86 (1H, d, *J* 5.0, H^5^), 8.22 (1H, t, *J* 2.0, H^2′^), 8.17–8.14 (1H, m, H^4′^ or H^6′^), 7.96 (1H, s, H^2^), 7.93 (1H, dd, *J* 2.1, 0.9, H^4′^ or H^6′^), 7.43 (1H, dd, *J* 5.1, 1.5, H^4^), 1.41 (9H, s, H^*t*-Bu^), 1.37 (12H, s, H^Bpin^). ^13^C NMR (151 MHz, CDCl_3_): δ_C_ 159.3 (C^q^), 151.3 (C^q^), 150.3 (C^5^), 133.2 (C^4′^ or C^6′^), 130.5 (C^4′^ or C^6′^), 127.2 (C^2′^), 117.3 (C^4^), 116.4 (C^2^), 83.9 (C^q^), 34.9 (C^Bpin-q^), 31.4 (C^*t*-Bu^), 25.0 (C^Bpin^). ^19^F NMR (376 MHz, CDCl_3_): δ_F_ −64.63 (CF_3_). MS ESI (ES^+^) *m*/*z* 406.4 [M + H]^+^.

#### 4.2.6 1-(3-Methoxypyridin-2-yl)-3-[3-(trifluoromethyl)pyridin-2-yl]-5-*tert*-butyl Benzene: HL^6^*

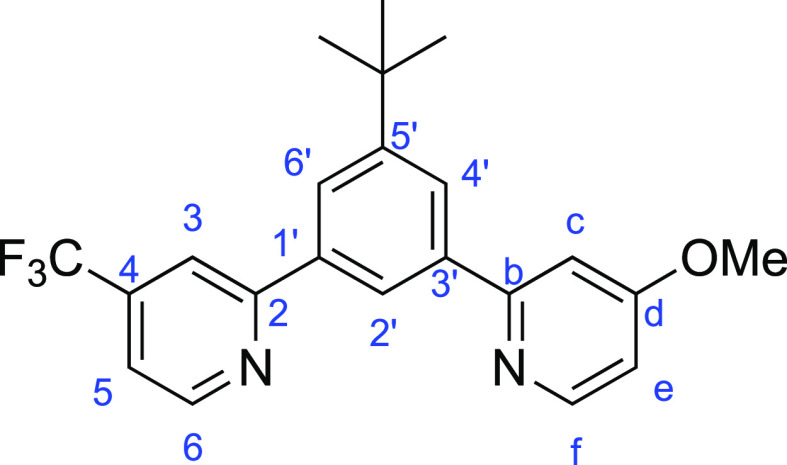



This proligand was prepared by Pd-catalyzed Suzuki cross-coupling of the product of the preceding reaction (486 mg, 1.20 mmol) with 2-chloro-4-methoxypyridine (172 mg, 1.20 mmol), aqueous Na_2_CO_3_ (1020 mg, 9.59 mmol), Pd(PPh_3_)_4_ (69 mg, 0.060 mmol), and DME (10 mL). The procedure was otherwise as described for HL^1^. The crude product was purified by column chromatography on silica (hexane/ethyl acetate gradient, *R*_f_ = 0.2 in 80:20) to yield the title compound as a yellow oil (151 mg, 33%). ^1^H NMR (700 MHz, CDCl_3_): δ_H_ 8.89 (1H, d, *J* 5.0, H^6^), 8.61 (1H, d, *J* 5.9, H^f^), 8.37 (1H, t, *J* 1.6, H^2′^), 8.17 (1H, s, H^6′^), 8.13 (1H, s, H^4′^), 8.05 (1H, s, H^3^), 7.48–7.43 (1H, m, H^5^), 7.34 (1H, d, *J* 2.4, H^c^), 6.88 (1H, s, H^e^), 3.98 (3H, s, H^OMe^), 1.47 (9H, s, H^*t*Bu^). ^13^C NMR (176 MHz, CDCl_3_): δ_C_ 150.5 (C^6^), 139.3, 138.5, 127.8, 125.9 (C^4ˈ^), 125.8, 123.7, 123.3 (C^2′^), 117.6 (C^5^), 116.5 (C^3^), 108.4 (C^e^), 107.9 (C^c^), 55.6 (C^OMe^), 31.4 (C^*t*-Bu^). MS ESI (ES^+^) *m*/*z* 387.3 ([M + H]^+^, 100%); HRMS (ES^+^) *m*/*z* 387.1676 [M + H]^+^, calcd for [C_22_H_22_N_2_OF_3_], 387.1684.

#### 4.2.7 PtL^6^*Cl

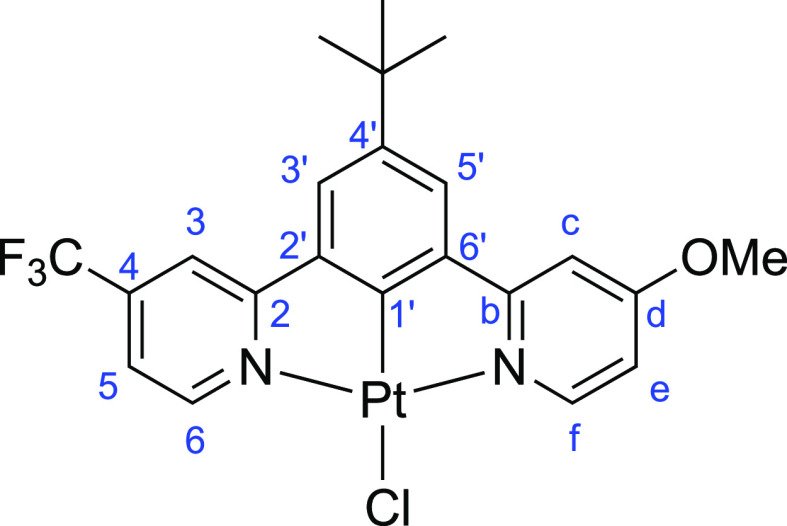



This complex was prepared as described for PtL^1^Cl but starting from HL^6^* (66 mg, 0.171 mmol) and K_2_PtCl_4_ (81 mg, 0.195 mmol) in acetic acid (6 mL) to yield the desired product as a yellow solid (62 mg, 59%). ^1^H NMR (700 MHz, CDCl_3_): δ_H_ 9.55 (1H, d, *J* 5.9, H^6^), 9.04 (1H, d, *J* 6.6, H^f^), 7.83 (1H, d, *J* 1.9, H^3^), 7.55 (1H, d, *J* 1.4, H^3ˈ^), 7.51 (1H, d, *J* 1.4, H^5ˈ^), 7.43 (1H, dd, *J* 6.1, 1.9, H^5^), 7.19 (1H, d, *J* 2.8, H^c^), 6.77 (1H, dd, *J* 6.6, 2.8, H^e^), 4.00 (3H, s, H^OMe^), 1.43 (9H, s, H^*t*Bu^). ^13^C NMR (176 MHz, CDCl_3_): δ_C_ 169.0 (C^q^), 168.7 (C^q^), 168.1 (C^q^), 159.1 (C^q^), 153.3 (C^f^), 153.0 (C^6^), 146.6 (C^q^), 140.9 (C^q^), 139.0 (C^q^), 122.2 (C^5ˈ^), 121.9 (C^3ˈ^), 118.9 (C^5^), 114.9 (C^3^), 108.0 (C^e^), 106.1 (C^c^), 56.1 (C^OMe^), 35.3 (C^*t*Bu^), 31.5 (C^*t*Bu^). HRMS (ASAP^+^) *m*/*z* 620.1404 [M-Cl + MeCN]^+^, calcd for [C_24_H_23_N_3_OF_3_^194^Pt], 620.1420. Anal. Calcd for C_22_H_20_ClF_3_N_2_OPt·0.4CH_2_Cl_2_: C, 41.40; H, 3.23; N, 4.31%. Found: C, 41.43; H, 3.16; N, 4.27%.

### 4.3 X-ray Crystallography

The X-ray single crystal data have been collected at a temperature of 120.0(2) K using Mo Kα radiation (λ = 0.71073 Å) on a Bruker D8 Venture (Photon III MM C7 CPAD detector, IμS-microsource, focusing mirrors, or Photon 100 CMOS detector, IμS-III-microsource, focusing mirrors) 3-circle diffractometer equipped with a Cryostream (Oxford Cryosystems) open-flow nitrogen cryostat. All structures were solved by various direct methods and refined by full-matrix least-squares on *F*^2^ for all data using Olex2^33^ and SHELXTL^34^ software. All nondisordered nonhydrogen atoms were refined in anisotropic approximation: hydrogen atoms were placed in the calculated positions and refined in the riding mode. Crystal data and parameters of refinement are listed in Supporting Information, Tables S2 to S4. Crystallographic data for all structures have been deposited with the Cambridge Crystallographic Data Centre as supplementary publications CCDC 2257828–2257835.

### 4.4 Solution-State Photophysics

UV–visible absorption spectra were recorded on a BioTek Instruments Uvikon XS spectrometer operated with LabPower software. Emission spectra were acquired on a Jobin Yvon Fluoromax-2 spectrometer equipped with a Hamamatsu R928 photomultiplier tube. All samples were contained within 1 cm path length quartz cuvettes modified for connection to a vacuum line. Degassing was achieved by at least three freeze–pump–thaw cycles while connected to the vacuum manifold: final vapor pressure at 77 K was <5 × 10^–2^ mbar. Emission was recorded at 90° to the excitation source, and spectra were corrected after acquisition for dark count and for the spectral response of the detector. The quantum yields were determined relative to an aqueous solution of [Ru(bpy)_3_]Cl_3_, for which Φ_lum_ = 0.04.^35^ Emission spectra at 77 K were recorded in 4 mm diameter tubes held within a liquid-nitrogen-cooled quartz Dewar, using the same spectrometer.

Luminescence lifetimes (τ) of <10 μs were measured by time-correlated single-photon counting using a pulsed-diode laser as an excitation source (405 nm excitation, pulse length of 60 ps, repetition rate 20 kHz or higher for shorter lifetimes). The emission was detected at 90° to the excitation source after passage through a monochromator, using an R928 PMT thermoelectrically cooled to −20 °C. Lifetimes ≥10 μs were recorded using the same detector operating in the multichannel scaling mode, following excitation with a microsecond pulsed xenon lamp. For all measurements, the decays were much longer than the instrument response, and the data were analyzed by least-squares tail fitting to eq 1
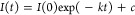
1where *I*(*t*) is the intensity of light detected at time *t*, *k* is the first-order rate constant for decay (*k* = 1/τ), and *c* is the constant reflecting the intrinsic “dark count” during the measurement. The quality of the fit was assessed by referring to the residuals (difference between fit and experimental data).

## Supporting Information Available

Synthetic and characterization data for proligands and complexes not presented in the main text; additional figures showing molecular structures and packing in the crystals; tables of crystal data and refinement parameters; frontier orbital plots obtained by DFT; and additional absorption and emission spectra (PDF)
