# Supplementary material for: Platinum(II) Complexes of Nonsymmetrical NCN-Coordinating Ligands: Unimolecular and Excimeric Luminescence Properties and Comparison with Symmetrical Analogues
Source: Inorg Chem. 2023 Jul 27;62(31):12356–71. doi: 10.1021/acs.inorgchem.3c01439 (PMC10410614; doi:10.1021/acs.inorgchem.3c01439)
Supplement: Supplementary file 1 — ic3c01439_si_001.pdf [file ic3c01439_si_001.pdf]

Supporting Information

Platinum(II) complexes of non-symmetrical *NCN*-coordinating ligands: unimolecular and excimeric luminescence properties and comparison with symmetrical analogues

*Rebecca J. Salthouse, Amit Sil, Louise F. Gildea, Dmitry S. Yufit, and J. A. Gareth Williams**

Department of Chemistry, Durham University, Durham, DH1 3LE, U.K.
** E-mail: j.a.g.williams@durham.ac.uk*

Section 1: Synthetic procedures and characterisation for proligands and complexes not presented in the main text.. **Page 2**

Section 2: X-ray crystallography details and additional figures of molecular and crystal structures .... **Page 11**

Section 3: Additional absorption / emission spectra and luminescence decay data...... **Page 17**

Section 4: Calculations using Density Functional Theory.................................. **Page 26**

Section 5: ^{1}H and ^{13}C NMR spectra .... **Page 29**

Section 1 Synthetic procedures and characterisation for proligands and complexes not presented in the main text

Generic experimental information, including details of the instrumentation employed and data for representative examples of the new families of complex, are given in the main text in the Experimental Section. For the compound characterisation details given in this Section, NMR coupling constants are given in Hz and chemical shifts are in ppm, referenced to residual protio solvent resonances for ^{1}H spectra, or to the ^{13}C of $CDCl_3$ in the case of ^{13}C spectra.

General procedures for cross-couplings

Suzuki cross-coupling reaction

The requisite boronic acid or ester derivative, halogenated pyridine/ isoquinoline (1 equiv.) and aqueous sodium carbonate (1 M, 8 equiv.) were added with ethylene glycol dimethyl ether (DME) to a Schlenk flask and the mixture was degassed using three freeze-pump-thaw cycles. Tetrakis(triphenylphosphine)-palladium(0) (5 mol %) was added under a flow of nitrogen and the reaction mixture heated at reflux under an atmosphere of nitrogen for 20 h. The crude mixture was washed with water and extracted into dichloromethane (DCM) before drying over anhydrous $MgSO_4$. The solution was filtered and the solvent removed under reduced pressure. The residue was purified by column chromatography using the conditions indicated in each case.

Miyaura cross-coupling reaction

The relevant bromobenzene, bis(pinacolato)diboron, B_2Pin_2, (1.2 equiv.) and potassium acetate (6 equiv.) were added with 1,4-dioxane (10 mL per mmol of substrate) to a dry Schlenk and the mixture was degassed by three freeze-pump-thaw cycles. [1,1′-Bis(diphenylphosphino)-ferrocene]dichloro-palladium(II) (10 mol %) was added under a flow of nitrogen and the reaction mixture heated at 80°C for 20 h. The solvent was removed under reduced pressure and the residue was extracted into DCM. After removal of the solvent under reduced pressure, the crude product was purified by column chromatography or used in further reactions without purification.

2-(3-Bromophenyl)pyridine – ppy-Br

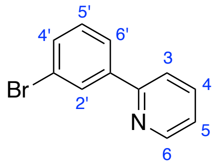

This key precursor was prepared by a Stille cross-coupling. 1,3-Dibromobenzene (250 mg, 1.06 mmol) and 2-(tributylstannyl)pyridine (488 mg, 1.33 mmol) were added with dry toluene (15 mL) and LiCl (360 mg, 8.48 mmol) to a dry Schlenk and desgassed by three freeze-pump-thaw cycles. $Pd(PPh_3)_2Cl_2$ (60 mg, 0.09 mmol) was added under a flow of nitrogen and the reaction mixture heated at reflux (110°C) for 48 h. Saturated $KF_{(aq)}$ was added to the solution and stirred at room temperature for 30 min. Subsequently the crude product was filtered, washed with toluene and the solvent removed under reduced pressure. The organic product was extracted into DCM, washed with 5% $NaHCO_3$ and dried over anhydrous $MgSO_4$ to give the crude product as a yellow/brown oily solid. The compound was purified by column chromatography on silica (hexane: ethyl acetate, gradient to 80:20, $R_f = 0.3$ in 90:10) to yield the title compound as a clear oil (111 mg, 45%); δ H (400 MHz, $CDCl_3$) 8.72 (1 H, ddd, J 4.8, 1.8, 1.0, H^6), 8.20 (1 H, t, J 1.9, $H^{2'}$), 7.93 (1 H, ddd, J 7.8, 1.8, 1.0, $H^{4'}$), 7.78 (1 H, ddd, J 8.0, 7.3, 1.1, H^4), 7.72 (1 H, dt, J 8.0, 1.1, H^3), 7.56 (1 H, ddd, J 7.9, 2.0, 1.0, $H^{6'}$), 7.36 (1 H, t, J 7.9, $H^{5'}$), 7.29-7.25 (1 H, m, H^5); δ C (100 MHz, $CDCl_3$) 155.9 (C^q), 149.8 (C^6), 141.4 (C^q), 136.9 (C^4), 131.9 ($C^{6'}$), 130.3 ($C^{5'}$), 130.1 ($C^{2'}$), 125.4 ($C^{4'}$), 123.1 (C^q), 122.7 (C^5), 120.6 (C^3); MS ESI (ES^+) *m/z* 233.3 (M^+, 100%).

This compound was also prepared *via* a Suzuki cross-coupling reaction, following the general procedure described above, with 2-bromopyridine (0.944 g, 5.98 mmol) and 3-bromophenylboronic acid (1.00 g, 4.98 mmol) with aqueous Na_2CO_3 (4.22 g, 39.8 mmol), $Pd(PPh_3)_4$ (0.288 g, 0.249 mmol) and DME (15 mL). The crude mixture was purified by column chromatography on silica (hexane: ethyl acetate gradient, $R_f = 0.3$ in 90:10) to yield the product as a clear oil (0.916 g, 79%).

2-(3-Bromophenyl)-4-(trifluoromethyl)pyridine – (CF_3)ppy-Br

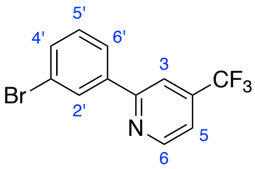

This compound was prepared *via* the Suzuki cross-coupling reaction using 3-bromophenylboronic acid (1.00 g, 4.98 mmol), 2-bromo-4-(trifluoromethyl)pyridine (1.35 g, 5.98 mmol), aqueous Na_2CO_3 (4.22 g, 39.83 mmol), $Pd(PPh_3)_4$ (288 mg, 0.25 mmol) and DME (15 mL). The crude mixture was purified by column chromatography (hexane: ethyl acetate slow gradient, $R_f = 0.5$ in 90:10) to yield

the title product as a white solid (707 mg, 47%); δ H (400 MHz, $CDCl_3$) 8.89 (1 H, dt, J 5.0, 0.8), 8.23 (1 H, t, J 1.8), 7.96 (1 H, ddd, J 7.8, 1.8, 1.0), 7.93 (1 H, s), 7.61 (1 H, ddd, J 7.9, 2.0, 1.0), 7.50 (1 H, ddd, J 5.1, 1.6, 0.8), 7.39 (1 H, t, J 7.9); δ F (376 MHz, $CDCl_3$) -64.79 (s); δ C (101 MHz, $CDCl_3$) 157.1, 150.8, 139.9, 139.5, 132.8, 130.5, 130.2, 125.5, 123.3, 121.5, 118.2, 116.1; MS ESI (ES^+) *m/z* 302.2 [M^+].

2-[3-(4,4,5,5-Tetramethyl-1,3,2-dioxaborolan-2-yl)phenyl]pyridine - (CF_3)ppy-Bpin

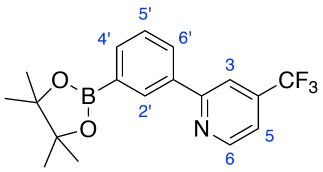

This compound was prepared *via* the Miyaura borylation reaction using **(CF_3)ppy-Br** (707 mg, 2.34 mmol), B_2Pin_2 (716 mg, 2.81 mmol), KOAc (1380 mg, 14.1 mmol), $PdCl_2$(dppf) (171 mg, 0.23 mmol) and 1,4-dioxane (20 mL). The crude product was used in further reactions without purification.

3-(4,4,5,5-Tetramethyl-1,3,2-dioxaborolan-2-yl)-5-*tert*-butyl-bromobenzene

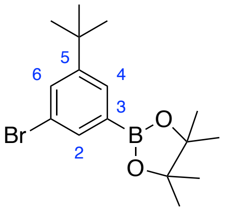

This compound was prepared *via* the Miyaura borylation reaction using 1,3-dibromo-5-*tert*-butylbenzene (1.00 g, 3.43 mmol), B_2pin_2 (0.873 g, 3.43 mmol), KOAc (2.02 g, 20.6 mmol) and $PdCl_2$(dppf) (0.251 g, 0.343 mmol) in 1,4-dioxane (35 mL). The crude product was used in further reactions without purification.

1,3-Di(3-isoquinolyl)benzene: HL^{1sym}

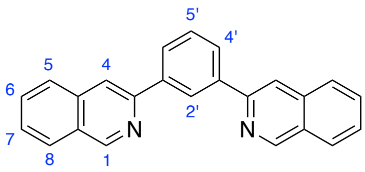

This compound was prepared *via* the Suzuki cross-coupling reaction using 1,3-phenylenediboronic acid (100 mg, 0.603 mmol), 3-bromoisoquoline (251 mg, 1.21 mmol), aqueous Na_2CO_3 (511 mg, 4.82 mmol), $Pd(PPh_3)_4$ (35 mg, 0.030 mmol) and DME (5 mL). The crude mixture was purified by column chromatography on silica (hexane: ethyl acetate, R_f = 0.4 in 70:30) to yield the product as a white solid (180 mg, 90%); δH (400 MHz, $CDCl_3$) 9.40 (2 H, d, J 1.0, H^1), 8.91 (1 H, td, J 1.9, 0.5, $H^{2'}$), 8.25 (2 H, s, H^4), 8.22 (2 H, dd, J 7.7, 1.8, $H^{4'}$), 8.03 (2 H, dq, J 8.2, 1.0, H^8), 7.93 (2 H, dq, J

8.6, 1.0, H^5), 7.72 (2 H, ddd, J 8.2, 6.8, 1.3, H^6), 7.67 (1 H, td, J 7.8, 0.5, $H^{5'}$), 7.61 (2 H, ddd, J 8.1, 6.9, 1.1, H^7); δC (100 MHz, $CDCl_3$) 152.4 (C^1), 151.2 (C^q), 140.1 (C^q), 136.7 (C^q), 130.6 (C^6), 129.3 ($C^{5'}$), 127.9 (C^q), 127.6 (C^8), 127.2 ($C^{4'}$ or C^7), 127.1 ($C^{4'}$ or C^7), 127.0 (C^5), 125.6 ($C^{2'}$), 116.8 (C^4); MS ESI (ES^+) *m/z* 333.3 ($[M+H]^+$, 100%).

1-(1-Isoquinolyl)-3-(2-pyridyl)benzene: HL^2

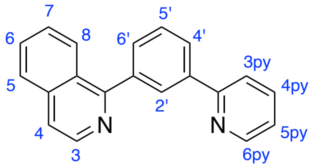

This proligand was prepared *via* the Suzuki cross-coupling reaction with **ppy–Bpin** (250 mg, 0.89 mmol), 1-chloroisoquinoline (175 mg, 1.07 mmol), aqueous Na_2CO_3 (754 mg, 7.11 mmol), $Pd(PPh_3)_4$ (51 mg, 0.04 mmol) and DME (7 mL). The crude mixture was purified by column chromatography (hexane: ethyl acetate, gradient to 70:30, R_f = 0.2 in 70:30) to yield the title compound as a yellow oil (106 mg, 42%); δ H (700 MHz, $CDCl_3$) 8.69 (1 H, ddd, J 4.8, 1.8, 0.9, H^{6py}), 8.63 (1 H, d, J 5.7, H^3), 8.32 – 8.28 (1 H, m, $H^{2'}$), 8.16 (2 H, ddq, J 16.4, 8.5, 1.1, H^8 and, $H^{4'}$ or $H^{6'}$), 7.90 (1 H, d, J 8.2, H^5), 7.81 (1 H, dt, J 8.0, 1.0, H^{3py}), 7.77 – 7.73 (2 H, m, H^{4py} and, $H^{4'}$ or $H^{6'}$), 7.70 (1 H, ddd, J 8.2, 6.8, 1.1, H^6), 7.69 – 7.67 (1 H, m, H^{6py}), 7.66 – 7.62 (1 H, m, H^3), 7.55 (1 H, ddd, J 8.3, 6.8, 1.2, H^7), 7.24 (1 H, ddd, J 7.5, 4.8, 1.2, H^{5py}); δ C (176 MHz, $CDCl_3$) 171.1 (C^q), 160.4 (C^q), 157.1 (C^q), 149.7 (C^{6py}), 141.9 (C^3), 139.6 (C^q), 136.9 (C^q), 136.8 ($C^{4'}$, $C^{6'}$ or C^{4py}), 130.4 ($C^{4'}$, $C^{6'}$, or C^{4py}), 130.2 (C^6), 128.8 ($C^{5'}$), 128.5 ($C^{2'}$), 127.6 ($C^{4'}$, $C^{6'}$ or C^8), 127.4 ($C^{4'}$, $C^{6'}$ or C^8), 127.3 (C^7), 127.0 (C^5), 126.8 (C^q), 122.3 (C^{5py}), 120.7 (C^{3py}), 120.1 (C^4); MS ESI (ES^+) *m/z* 283.4 ($[M+H]^+$, 100%); HRMS (ES^+) *m/z* 283.1238 $[M+H]^+$, calc. for $[C_{20}H_{15}N_2]$ 283.1235.

1,3-Di(1-isoquinolyl)benzene: HL^{2sym}

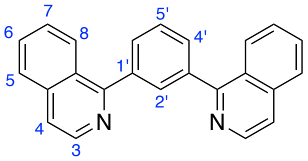

This ligand was prepared *via* the Suzuki cross-coupling reaction with benzene-1,3-diboronic acid (101 mg, 0.61 mmol), 1-chloroisoquinoline (200 mg, 1.22 mmol), aqueous Na_2CO_3 (518 mg, 4.89 mmol), $Pd(PPh_3)_4$ (35 mg, 0.03 mmol) and DME (7.5 mL) to yield the crude product as a brown oil. The product was purified by column chromatography on silica (hexane: ethyl acetate, gradient to 70:30, R_f = 0.3 in 50:50) to yield the title proligand as an off-white solid (149 mg, 73%); δ H (700 MHz; $CDCl_3$) 8.62 (2 H, d, J 5.7, H^3), 8.20 (2 H, d, J 8.5, H^8), 8.03 (1 H, t, J 1.7, $H^{2'}$), 7.88 (2 H, d, J 8.4, H^5), 7.86 (2 H, dd, J 7.6, 1.8, $H^{4'}$), 7.72 (1 H, t, J 7.6, $H^{5'}$), 7.70-7.64 (4 H, m, H^4 and H^6), 7.54 (2

H, t, J 7.7, H^7); δ C (400 MHz; $CDCl_3$) 160.4 (C^q), 142.3 (C^3), 139.8 (C^q), 131.5 ($C^{2'}$), 130.2 ($C^{4'}$), 130.1 ($C^{5'}$), 128.6 (C^4), 127.6 (C^8), 127.3 (C^7), 127.0 (C^5), 126.8 (C^q), 120.1 (C^6); MS ESI (ES^+) *m/z* 333.4 ($[M+H]^+$, 100%).

1-Pyrimidyl-3-(2-pyridyl)benzene: HL^3

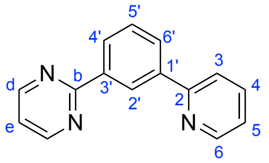

This proligand was prepared *via* the Suzuki cross-coupling reaction using **ppy-Bpin** (275 mg, 0.978 mmol), 2-bromopyrimidine (155 mg, 0.978 mmol), aqueous Na_2CO_3 (829 mg, 7.82 mmol), $Pd(PPh_3)_4$ (57 mg, 0.049 mmol) and DME (8 mL). The crude mixture was purified by column chromatography on silica (hexane: ethyl acetate gradient, R_f = 0.3 in 70:30) to yield the title proligand as a yellow oil (102 mg, 45%); δ H (400 MHz, $CDCl_3$) 9.08 (1 H, s, $H^{2'}$), 8.87 (2 H, d, J 4.8, H^d), 8.76 (1 H, dd, J 4.9, 1.5, H^6), 8.53 (1 H, dd, J 7.8, 1.5, $H^{4'}$ or $H^{6'}$), 8.22 (1 H, dd, J 7.8, 1.7, $H^{4'}$ or $H^{6'}$), 7.95 – 7.89 (1 H, m, H^3), 7.82 (1 H, tt, J 7.8, 1.4, H^4), 7.65 (1 H, t, J 7.8, $H^{5'}$), 7.32 – 7.29 (1 H, m, H^5), 7.25 (1 H, t, J 4.8, H^e); δ C (101 MHz, $CDCl_3$) 164.5 (C^b), 157.3 (C^d), 157.0 (C^2), 149.6 (C^6), 139.7 ($C^{1'}$ or $C^{3'}$), 138.1 ($C^{1'}$ or $C^{3'}$), 137.0 (C^4), 129.4 ($C^{4'}$ or $C^{6'}$), 129.2 ($C^{5'}$), 128.7 ($C^{4'}$ or $C^{6'}$), 126.7 ($C^{2'}$), 122.3 (C^5), 120.8 (C^3), 119.3 (C^e). MS ES (ES^+) *m/z* 234.2 ($[M+H]^+$, 100%); HRMS (ES^+) *m/z* 234.1038 $[M+H]^+$, calc. for $[C_{15}H_{12}N_3]$ 234.1031.

2-[3-(pyridin-2-yl)phenyl]-4-(trifluoromethyl)pyridine: HL^4

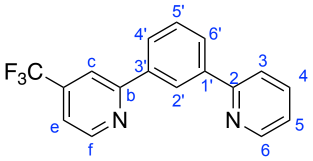

This compound was prepared by a Suzuki cross-coupling reaction with **ppy-Bpin** (295 mg, 1.05 mmol), 2-bromo-4-(trifluoromethyl)pyridine (237 mg, 1.05 mmol), aqueous Na_2CO_3 (889 mg, 8.39 mmol), $Pd(PPh_3)_4$ (60 mg, 0.052 mmol) and DME (8 mL). The crude mixture was purified by column chromatography on silica (hexane: ethyl acetate gradient, R_f = 0.3 in 70:30) to yield the title compound as a clear oil (19 mg, 6%); δ H (700 MHz, $CDCl_3$) 8.89 (1 H, d, J 5.0, H^f), 8.74 (1 H, ddd, J 4.8, 1.8, 1.0, H^6), 8.67 (1 H, t, J 1.9, $H^{2'}$), 8.12 – 8.08 (2 H, m, $H^{4'}$ and $H^{6'}$), 8.04 (1 H, s, H^c), 7.85 (1 H, dt, J 8.0, 1.1, H^3), 7.80 (1 H, td, J 7.6, 1.8, H^4), 7.64 – 7.59 (1 H, m, $H^{5'}$), 7.47 (1 H, ddd, J 5.1, 1.6, 0.8, H^e), 7.28 (1 H, ddd, J 7.4, 4.8, 1.2, H^5); δ C (176 MHz, $CDCl_3$) 158.5 (C^a), 156.8 (C^2), 150.6 (C^f), 149.6 (C^6), 140.0 ($C^{3'}$), 139.2 (q, C^d), 138.6 ($C^{1'}$), 137.0 (C^4), 129.4 ($C^{5'}$), 128.3 ($C^{4'}$ or $C^{6'}$), 127.6 ($C^{4'}$ or $C^{6'}$), 125.6 ($C^{2'}$), 123.7 (C^{CF_3}), 122.5 (C^5), 120.8 (C^3), 117.7 (C^e), 116.2 (C^c); δ F (376

MHz, $CDCl_3$) -64.7; MS ESI (ES^+) *m/z* 302.1 ($[M+H]^+$, 100%); HRMS (ES^+) *m/z* 301.0952 $[M+H]^+$, calc. for $[C_{17}H_{12}N_2F_3]$ 301.0953.

4-Methoxy-2-[3-(pyridin-2-yl)phenyl]pyridine: HL^5

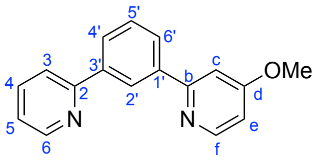

This compound was prepared *via* the Suzuki cross-coupling reaction using **ppy-Bpin** (295 mg, 1.05 mmol), 2-chloro-4-methoxypyridine (151 mg, 1.05 mmol), aqueous Na_2CO_3 (889 mg, 8.39 mmol), $Pd(PPh_3)_4$ (60 mg, 0.052 mmol) and DME (8 mL). The crude mixture was purified by column chromatography on silica (hexane: ethyl acetate gradient, $R_f = 0.2$ in 60:40) to yield the title product as a clear oil (110 mg, 48%); δ H (599 MHz, $CDCl_3$) 8.70 (1 H, ddd, J 4.7, 1.9, 1.0, H^6), 8.58 (1 H, t, J 1.8, $H^{2'}$), 8.52 (1 H, d, J 5.8, H^f), 8.03 (2 H, ddt, J 18.1, 8.0, 1.4, $H^{4'}$ and $H^{6'}$), 7.81 (1 H, dt, J 8.0, 1.1, H^3), 7.73 (1 H, td, J 7.7, 1.9, H^4), 7.56 (1 H, t, J 7.7, $H^{5'}$), 7.32 (1 H, d, J 2.4, H^c), 7.22 (1 H, ddd, J 7.4, 4.8, 1.2, H^5), 6.77 (1 H, dd, J 5.7, 2.4, H^e), 3.88 (3 H, d, J 1.9, H^{OMe}); δ C (151 MHz, $CDCl_3$) 166.4 (C^d), 158.9 (C^b), 157.2 (C^2), 150.8 (C^f), 149.6 (C^6), 139.9 ($C^{1'}$ and $C^{3'}$), 136.7 (C^4), 129.1 ($C^{5'}$), 127.5 ($C^{4'}$ and $C^{6'}$), 125.5 ($C^{2'}$), 122.2 (C^5), 120.7 (C^3), 108.3 (C^3), 107.0 (C^c), 55.2 (C^{OMe}); MS ESI (ES^+) *m/z* 264.1 ($[M+H]^+$, 100%); HRMS (ES^+) *m/z* 263.1173 $[M+H]^+$, calc. for $[C_{17}H_{15}N_2O]$ 263.1184.

2-[3-(4-Methoxypyridin-2-yl)phenyl]-4-(trifluoromethyl)pyridine: HL^6

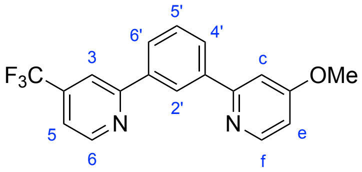

This proligand was prepared *via* the Suzuki cross-coupling reaction using 2-chloro-4-methoxypyridine (395 mg, 2.75 mmol), crude **(CF_3)ppy-Bpin** (800 mg, 2.29 mmol), aqueous Na_2CO_3 (1940 mg, 18.33 mmol), $Pd(PPh_3)_4$ (132 mg, 0.12 mmol) and DME (10 mL). The crude mixture was purified by column chromatography (hexane: ethyl acetate gradient, $R_f = 0.1$ in 80:20) to yield the product as a yellow oil (63 mg, 8%); δ H (600 MHz, $CDCl_3$) 8.88 (1 H, dt, J 5.1, 0.7, H^6), 8.63 (1 H, t, J 1.9, $H^{2'}$), 8.56 (1 H, d, J 5.7, H^c), 8.10 (1 H, ddd, J 7.7, 1.9, 1.0, $H^{4'}$ or $H^{6'}$), 8.06 (1 H, ddd, J 7.8, 1.8, 1.1, $H^{4'}$ or $H^{6'}$), 8.04 (1 H, dt, J 1.7, 0.9, H^3), 7.61 (1 H, td, J 7.8, 0.6, $H^{5'}$), 7.46 (1 H, ddd, J 5.0, 1.6, 0.8, H^5), 7.33 (1 H, d, J 2.4, H^f), 6.82 (1 H, dd, J 5.7, 2.4, H^e), 3.93 (3 H, s, H^{OMe}); δ C (151 MHz, $CDCl_3$) 166.6 (C^q), 158.5 (C^q), 150.8 (C^c), 150.6 (C^6), 139.3 (C^q), 139.0 (C^q), 138.5 (C^q), 129.4 ($C^{5'}$), 128.4 ($C^{4'}$ or $C^{6'}$), 127.7 ($C^{4'}$ or $C^{6'}$), 125.7 ($C^{2'}$), 117.7 (C^5), 116.2 (C^3), 108.3 (C^e),

107.2 (C^f), 55.3 (C^{OMe}); MS ESI (ES+) m/z 331.3 ([M+H]+, 100%); HRMS (ES^+) *m/z* 331.1069 $[M+H]^+$, calc. for [$C_{18}H_{14}N_2OF_3$] 331.1058.

General procedure for complexation

Potassium tetrachloroplatinate(II) was added to a solution of the appropriate ligand in acetic acid in a dry Schlenk flask and the solution was degassed using three freeze-pump-thaw cycles. The reaction mixture was then heated at reflux (118°C) for 60 h under nitrogen before cooling to room temperature. Water was added and the solid product separated on the centrifuge; the crude product was washed with water, methanol, and then finally diethyl ether. The solid product was extracted into DCM and the solvent removed under reduced pressure to give the desired complex.[4]

PtL²Cl

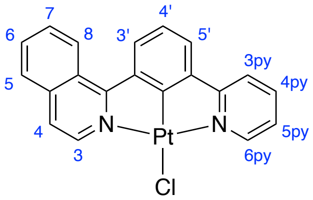

This complex was prepared by the general complexation procedure outlined above with **HL²** (54 mg, 0.19 mmol) and K_2PtCl_4 (90 mg, 0.22 mmol) in acetic acid (5 mL) to yield the title complex as an orange solid (39 mg, 40%); δ H (700 MHz, $CDCl_3$) 9.42 (2 H, dd, J 9.4, 6.1, H^3 and H^{6py}), 8.93 (1 H, d, J 8.7, H^5), 8.19 (1 H, d, J 7.9, $H^{3'}$ or $H^{5'}$), 7.96 (1 H, td, J 7.8, 1.6, H^{4py}), 7.91 (1 H, d, J 8.2, H^8), 7.85 – 7.78 (1 H, m, H^7), 7.77 – 7.70 (2 H, m, H^6 and H^{3py}), 7.62 (1 H, d, J 6.4, H^4), 7.52 (1 H, d, J 7.6, $H^{3'}$ or $H^{5'}$), 7.32 (2 H, td, J 7.5, 3.7, H^{5py} and $H^{5'}$); δ C (176 MHz, $CDCl_3$) 151.8 (C^{6py}), 144.1 (C^3), 139.0 (C^{4py}), 131.7 (C^7), 129.0 ($C^{3'}$ or $C^{5'}$), 128.7 (C^6), 127.9 (C^8), 125.4 (C^5), 124.1 ($C^{3'}$ or $C^{5'}$), 123.2 (C^{5py}), 122.8 ($C^{4'}$), 121.7 (C^4), 119.1 (C^{3py}), the quaternary carbon peaks were not observed due to the low solubility of the complex; MS ASAP (AP^+) *m/z* 517.1 ($[M\text{-}Cl+MeCN]^+$, 100%); HRMS (AP^+) 516.0969 $[M\text{-}Cl+MeCN]^+$, calc. for [$C_{22}H_{16}N_3{}^{194}Pt$] 516.0971; Anal. calc. for $C_{20}H_{13}ClN_2Pt{\cdot}0.1CH_2Cl_2$: C, 46.39; H, 2.56; N, 5.38 %; Found C, 46.29; H, 2.68; N, 5.22 %.

PtL³Cl

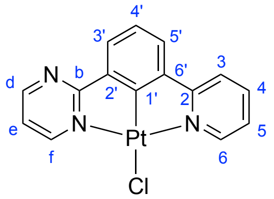

This complex was prepared using the complexation general procedure outlined above with **HL³** (62 mg, 0.266 mmol) and K_2PtCl_4 (126 mg, 0.303 mmol) in acetic acid (6 mL) to yield the product as an

orange solid (95 mg, 77%); δ H (700 MHz, $CDCl_3$) 9.48 (1 H, dd, J 5.7, 2.2, H^f), 9.29 (1 H, dt, J 5.7, 0.9, H^6), 8.91 (1 H, dd, J 4.8, 2.3, H^d), 7.96 (1 H, td, J 7.8, 1.6, H^4), 7.75 (1 H, dd, J 7.6, 0.9, $H^{3'}$), 7.72 (1 H, dd, J 7.9, 1.3, H^3), 7.54 (1 H, dd, J 7.7, 0.9, $H^{5'}$), 7.30 (1 H, ddd, J 7.3, 5.6, 1.4, H^5), 7.28 (1 H, t, J 7.6, $H^{4'}$), 7.26 (1 H, dd, J 4.3, 1.4, H^e); δ C (176 MHz, $CDCl_3$) 175.3 (C^q), 166.6 (C^q), 161.2 (C^q), 158.3 (C^f), 158.2 (C^d), 152.3 (C^6), 141.1 (C^q), 139.4 (C^4), 137.8 (C^q), 127.6 ($C^{3'}$), 126.0 ($C^{5'}$), 123.4 ($C^{4'}$), 123.3 (C^5), 119.3 (C^3), 118.6 (C^e); MS ASAP (AP^+) *m/z* 468.1 ($[M\text{-}Cl\text{+}MeCN]^+$, 100%); HRMS ($AP^+$) *m/z* 466.0807 $[M\text{-}Cl\text{+}MeCN]^+$, calc. for $[C_{17}H_{14}N_4{}^{192}Pt]$ 466.0829; Anal. calc. for $C_{15}H_{10}ClN_3Pt{\cdot}0.5CH_2Cl_2$: C, 36.85; H, 2.19; N, 8.32 %; Found C, 36.56; H, 2.05; N, 8.21 %.

PtL⁴Cl

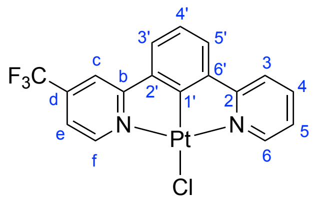

This complex was prepared using the general procedure for complexation outlined above with **HL⁴** (19 mg, 0.063 mmol) and K_2PtCl_4 (30 mg, 0.072 mmol) in acetic acid (4 mL) to yield the product as a yellow solid (19 mg, 57%); δ H (700 MHz, $CDCl_3$) 9.56 (1 H, d, J 5.9, H^f), 9.36 – 9.25 (1 H, m, H^6), 7.96 (1 H, td, J 7.7, 1.6, H^4), 7.83 (1 H, s, H^c), 7.71 (1 H, d, J 7.9, H^3), 7.54 – 7.48 (2 H, m, $H^{3'}$ and $H^{5'}$), 7.48 – 7.45 (1 H, m, H^e), 7.31 (1 H, ddd, J 7.3, 5.6, 1.5, H^5), 7.27 (1 H, d, J 7.7, $H^{4'}$); δ C (176 MHz, $CDCl_3$) 168.8 (C^q), 167.1 (C^q), 162.7 (C^q), 153.1 (C^f), 152.4 (C^6), 141.2 (C^q), 139.5 (C^q), 139.4 (C^4), 125.0 ($C^{3'/5'}$), 124.6 ($C^{3'/5'}$), 123.4 (C^5), 123.2 ($C^{4'}$), 119.4 (C^3), 119.1 (C^e), 115.1 (C^c); HRMS (AP^+) *m/z* 534.0677 $[M\text{-}Cl\text{+}MeCN]^+$, calc. for $[C_{19}H_{13}N_3F_3{}^{194}Pt]$ 534.0688; Anal. calc. for $C_{17}H_{10}ClF_3N_2Pt$: C, 38.54; H, 1.90; N, 5.29 %; Found C, 38.94; H, 2.04; N, 4.65 %.

PtL⁵Cl

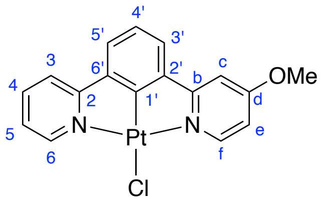

This complex was prepared using the general procedure for complexation outlined above with **HL⁵** (110 mg, 0.419 mmol) and K_2PtCl_4 (198 mg, 0.478 mmol) in acetic acid (11 mL) to yield the product as a yellow solid (68 mg, 34%); δ H (700 MHz, $CDCl_3$) 9.39 – 9.29 (1 H, m, H^6), 9.07 (1 H, d, J 6.6, H^f), 7.92 (1 H, td, J 7.7, 1.6, H^4), 7.68 – 7.63 (1 H, m, H^3), 7.42 (1 H, dd, J 7.6, 0.8, $H^{5'}$), 7.38 (1 H, dd, J 7.7, 0.8, $H^{3'}$), 7.28 – 7.23 (2 H, m, H^5), 7.19 (1 H, t, J 7.6, $H^{4'}$), 7.13 (1 H, d, J 2.8, H^c), 6.75 (1 H, dd, J 6.6, 2.8, H^e), 3.97 (3 H, s, H^{OMe}); δ C (176 MHz, $CDCl_3$) 168.6 (C^b), 167.9 (C^d), 167.2 (C^2),

161.4 ($C^{6'}$), 153.1 (C^{f}), 152.1 (C^{6}), 140.9 ($C^{2'}$), 138.8 (C^{4}), 124.1 ($C^{5'}$), 124.0 ($C^{3'}$), 123.1 (C^{5}), 122.7 ($C^{4'}$), 119.1 (C^{3}), 108.1 (C^{e}), 105.9 (C^{c}), 56.0 (C^{OMe}); MS ASAP (AP^{+}) *m/z* 456.1 ($[M\text{-}Cl]^{+}$, 100%); HRMS (AP^{+}) *m/z* 455.0669 $[M\text{-}Cl]^{+}$, calc. for $[C_{17}H_{13}N_{2}O^{194}Pt]$ 455.0655; Anal. calc. for $C_{17}H_{13}ClN_{2}OPt$: C, 41.52; H, 2.66; N, 5.70 %; Found C, 40.94; H, 2.63; N, 5.58 %.

PtL^{6}Cl

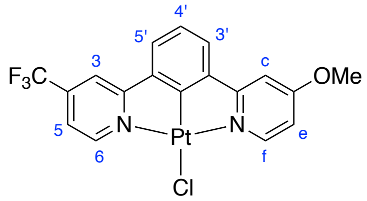

This complex was prepared by the complexation general procedure described above with **HL^{6}** (54 mg, 0.16 mmol) and K_2PtCl_4 (77 mg, 0.19 mmol) in acetic acid (5 mL) to yield the desired product as a yellow solid (69 mg, 76%); δ H (700 MHz, DMSO-d_6) 9.34 (1 H, d, J 6.0, H^{6}), 8.80 (1 H, d, J 6.6, H^{f}), 8.50 (1 H, d, J 2.0, H^{3}), 7.97 – 7.95 (1 H, m, $H^{3'}$ or $H^{5'}$), 7.88 (1 H, dd, J 6.0, 2.0 $H^{3'}$ or $H^{5'}$), 7.86 – 7.84 (1 H, m, H^{e}), 7.71 (1 H, d, J 2.9, H^{c}), 7.29 (1 H, t, J 7.7, $H^{4'}$), 7.14 (1 H, dd, J 6.7, 2.9, H^{5}), 4.00 (4 H, s, H^{OMe}); MS ASAP (AP^{+}) *m/z* 565.1 ($[M\text{-}Cl\text{+}MeCN]^{+}$, 100%); HRMS ($AP^{+}$) *m/z* 564.0810 $[M\text{-}Cl\text{+}MeCN]^{+}$, calc. for $[C_{20}H_{15}N_{3}OF_{3}{}^{194}Pt]$ 546.0794; Anal. calc. for $C_{18}H_{12}ClF_{3}N_{2}OPt$: C, 38.62; H, 2.16; N, 5.00 %; Found C, 38.11; H, 2.11; N, 4.86 %. This sample was too insoluble in standard NMR solvents to obtain a ^{13}C NMR spectrum.

Section 2 X-ray crystallography details and additional figures of molecular and crystal structures

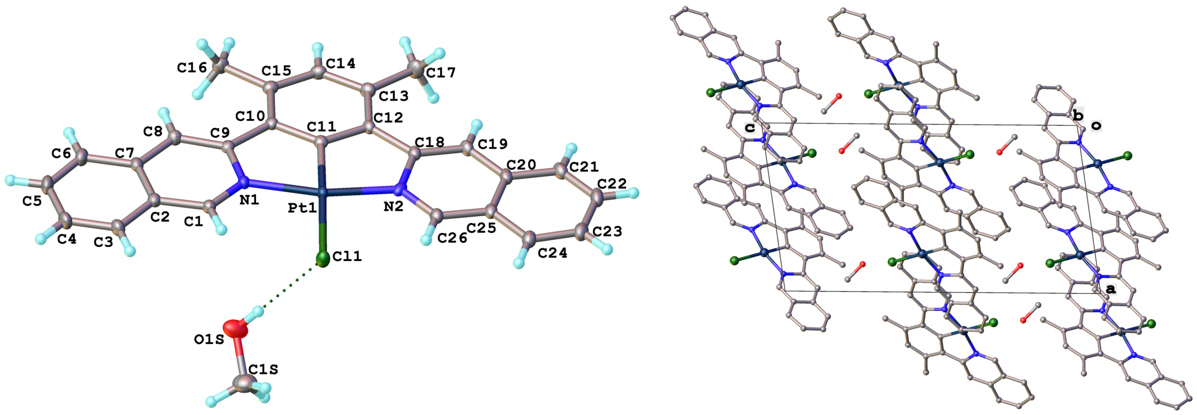

Figure S1 *Molecular structure and crystal packing of PtL^{1symMe2}Cl as a MeOH solvate.*

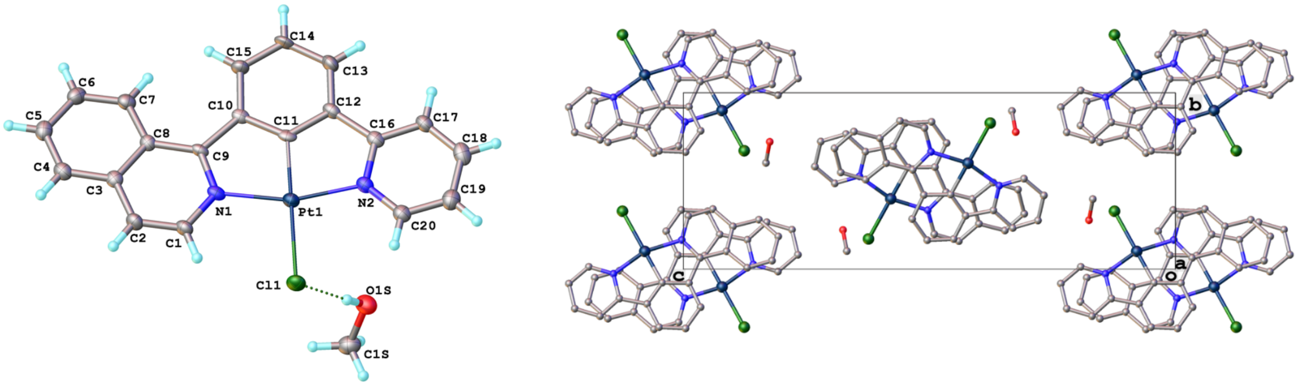

Figure S2 *Molecular structure and crystal packing of* ***PtL^{2}Cl*** *as a MeOH solvate.*

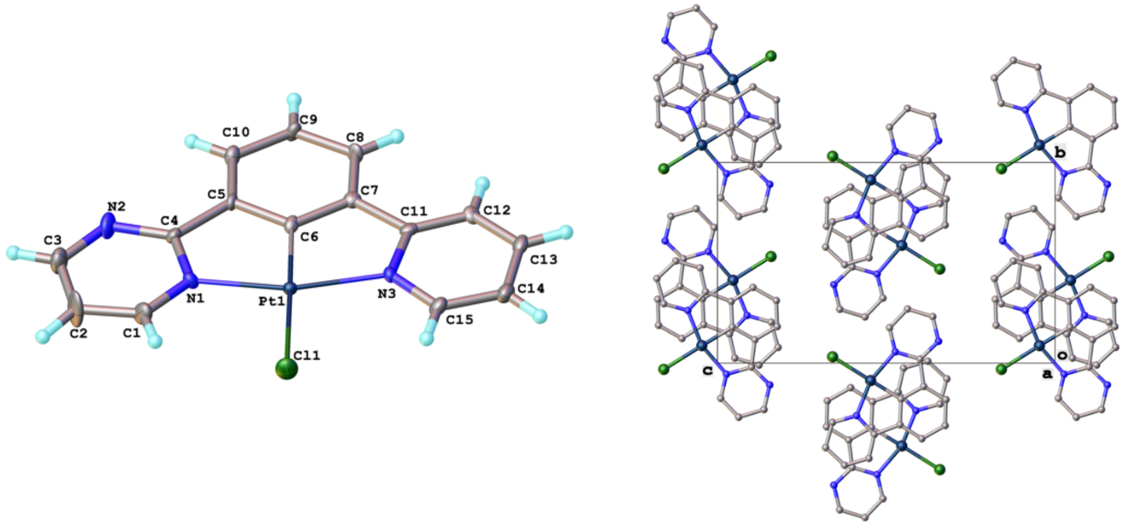

Figure S3 *Molecular structure and crystal packing of* ***PtL^{3}Cl****.*

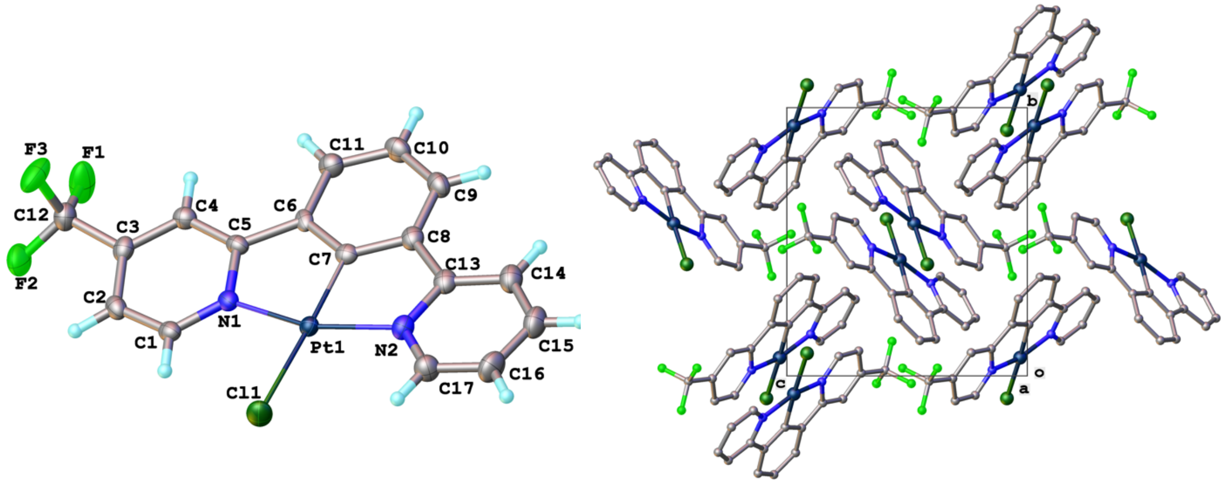

Figure S4 *Molecular structure and crystal packing of* ***PtL^{4}Cl***.

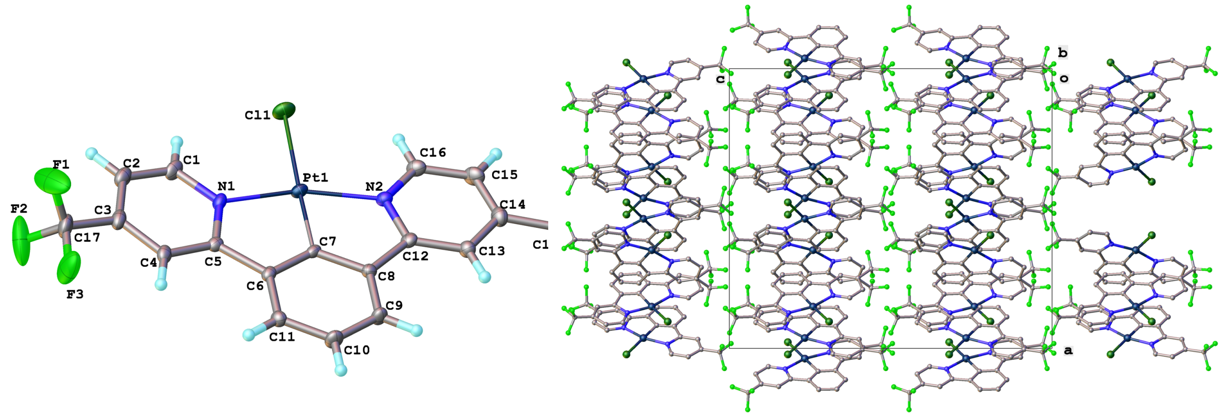

Figure S5 *Molecular structure and crystal packing of* ***PtL^{4sym}Cl***.

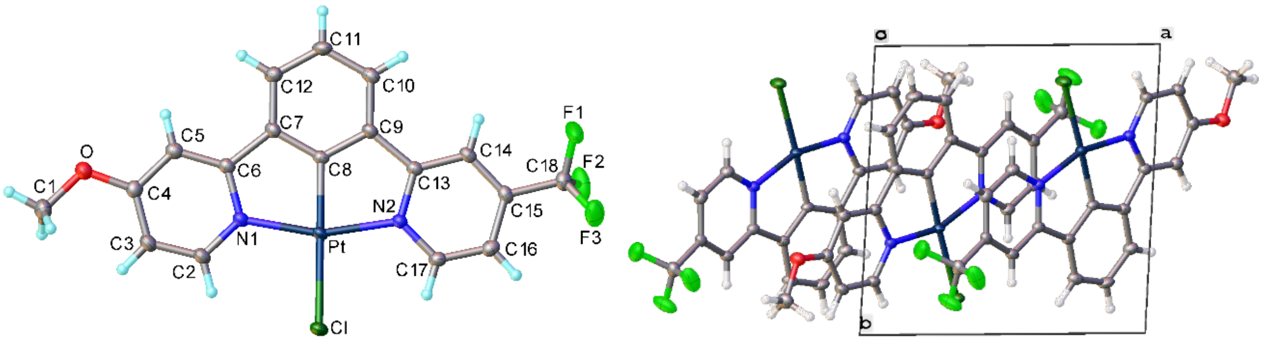

Figure S6 *Molecular structure and crystal packing of* ***PtL^{6}Cl***.

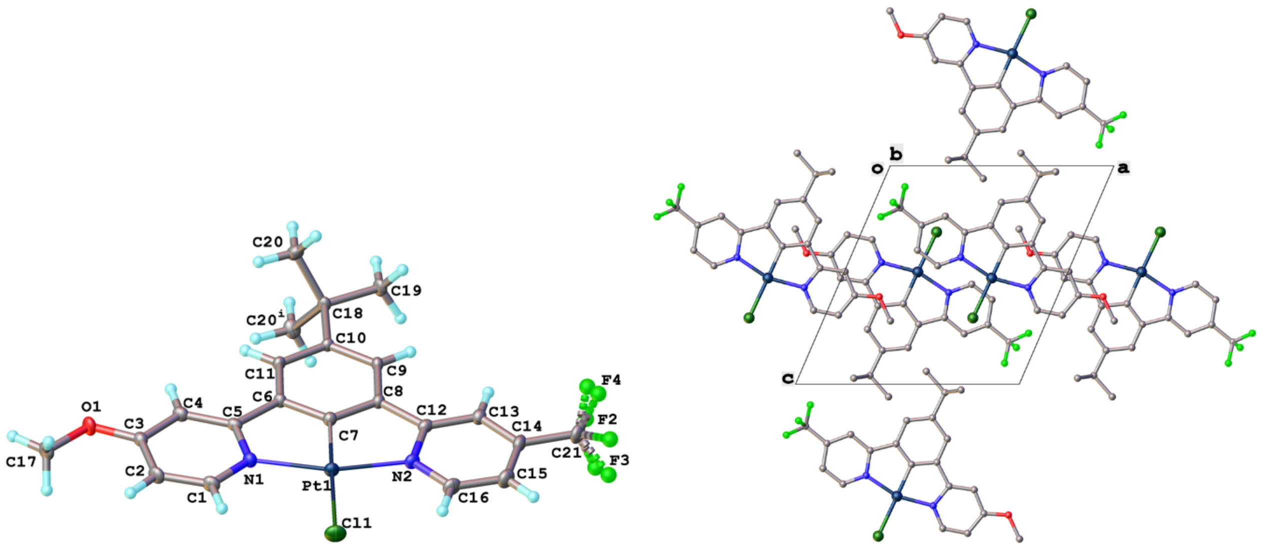

Figure S7 *Molecular structure and crystal packing of* ***PtL^{6}*Cl****.*

Table S1 *A summary of intermolecular distances and packing arrangements in the crystal structures presented.*

Complex	Pt···Pt distance / Å	Interplanar distance / Å	Packing arrangement
**PtL^{1}Cl**	5.3724(7)	3.494(2)	Head-to-tail
**PtL^{1symMe2}Cl**	5.8847(6)	3.7206(17)	Head-to-tail
**PtL^{2}Cl**	5.5064(8)	3.361(5)	Head-to-tail
**PtL^{3}Cl**	5.1151(8)	3.352(15)	Head-to-tail
**PtL^{4}Cl**	5.2323(6)	3.389(4)	Head-to-tail
**PtL^{4sym}Cl**	3.3884(5)	3.3883(4)	Staggered ~60°
**PtL^{6}Cl**	5.1394(5)	3.7559(18)	Head-to-tail
**PtL^{6}*Cl**	6.8899(3)	4.1472(13)	Head-to-tail

***Table S2** Crystal data and structure refinement for complexes **$PtL^{1-3}Cl$**, each featuring one unsubstituted pyridine, and 3-isoquinoline, 1-isoquinoline or pyrimidine respectively.*

	**PtL^1Cl**	**PtL^2Cl**	**PtL^3Cl**
Identification code	21srv073	20srv221	20srv237
Empirical formula	$C_{20}H_{13}ClN_2Pt$	$C_{21}H_{17}ClN_2OPt$	$C_{15}H_{10}ClN_3Pt$
Formula weight	511.86	543.91	462.80
Temperature/K	120.0	120.0	120.0
Crystal system	monoclinic	monoclinic	orthorhombic
Space group	$P2_1/c$	$P2_1/n$	$P2_12_12_1$
a/Å	8.8991(5)	7.2548(4)	6.6652(4)
b/Å	9.8598(5)	9.1840(5)	10.7512(6)
c/Å	18.7913(10)	25.6786(14)	18.0704(11)
α/°	90	90	90
β/°	91.629(2)	94.616(2)	90
γ/°	90	90	90
Volume/$Å^3$	1648.15(15)	1705.37(16)	1294.90(13)
Z	4	4	4
$\rho_{calc}g/cm^3$	2.063	2.118	2.374
μ/mm^{-1}	8.678	8.397	11.033
F(000)	968.0	1040.0	864.0
Crystal size/mm^3	0.12 × 0.05 × 0.04	0.13 × 0.12 × 0.01	0.08 × 0.03 × 0.01
Radiation	MoKα (λ = 0.71073)	Mo Kα (λ = 0.71073)	Mo Kα (λ = 0.71073)
2Θ range for data collection/°	4.336 to 59.996	4.712 to 59.998	4.408 to 57.996
Index ranges	$-12 \le h \le 12$, $-13 \le k \le 13$, $-26 \le l \le 26$	$-10 \le h \le 10$, $-12 \le k \le 12$, $-36 \le l \le 36$	$-9 \le h \le 9$, $-14 \le k \le 14$, $-24 \le l \le 24$
Reflections collected	38105	30303	19030
Independent reflections	4776 [R_{int} = 0.0344, R_{sigma} = 0.0197]	4974 [R_{int} = 0.0770, R_{sigma} = 0.0533]	3434 [R_{int} = 0.0708, R_{sigma} = 0.0524]
Data/restraints/parameters	4776/0/217	4974/0/237	3434/18/191
Goodness-of-fit on F^2	1.058	1.156	1.072
Final R indexes [I>=2σ (I)]	R_1 = 0.0153, wR_2 = 0.0345	R_1 = 0.0426, wR_2 = 0.0738	R_1 = 0.0420, wR_2 = 0.0941
Final R indexes [all data]	R_1 = 0.0179, wR_2 = 0.0354	R_1 = 0.0570, wR_2 = 0.0782	R_1 = 0.0475, wR_2 = 0.0964
Largest diff. peak/hole / e $Å^{-3}$	0.79/-0.76	1.17/-2.48	1.80/-3.43

***Table S3** Crystal data and structure refinement for complexes **PtL⁴Cl, PtL⁶Cl** and **PtL⁷Cl** featuring two pyridine rings.*

	PtL^4Cl	PtL^6Cl	PtL^7Cl
Identification code	21srv104	19srv240	21srv112
Empirical formula	$C_{17}H_{10}ClF_3N_2Pt$	$C_{18}H_{12}ClF_3N_2OPt$	$C_{22}H_{20}ClF_3N_2OPt$
Formula weight	529.81	559.84	615.94
Temperature/K	120.0	120	120.0
Crystal system	monoclinic	triclinic	monoclinic
Space group	$P2_1/c$	P-1	$P2_1/m$
a/Å	12.4233(5)	9.1299(6)	12.1952(5)
b/Å	11.6995(4)	9.2461(6)	6.8897(3)
c/Å	11.3735(4)	9.6998(7)	12.9731(5)
α/°	90	95.250(2)	90
β/°	112.9130(10)	96.120(2)	113.4220(10)
γ/°	90	92.824(2)	90
Volume/Å^3	1522.66(10)	809.29(9)	1000.20(7)
Z	4	2	2
ρ_{calc}g/cm^3	2.311	2.297	2.045
μ/mm^{-1}	9.424	8.877	7.192
F(000)	992.0	528.0	592.0
Crystal size/mm^3	0.16 × 0.15 × 0.04	0.255 × 0.118 × 0.068	0.18 × 0.11 × 0.01
Radiation	MoKα (λ = 0.71073)	MoKα (λ = 0.71073)	MoKα (λ = 0.71073)
2Θ range for data collection/°	7.122 to 59.996	4.432 to 66.424	5.906 to 59.994
Index ranges	$-17 \le h \le 17$, $-16 \le k \le 16$, $-16 \le l \le 16$	$-14 \le h \le 14$, $-14 \le k \le 13$, $-14 \le l \le 14$	$-17 \le h \le 17$, $-9 \le k \le 9$, $-18 \le l \le 18$
Reflections collected	26494	18547	17936
Independent reflections	4410 [R_{int} = 0.0300, R_{sigma} = 0.0205]	6175 [R_{int} = 0.0328, R_{sigma} = 0.0406]	3125 [R_{int} = 0.0335, R_{sigma} = 0.0231]
Data/restraints/parameters	4410/0/225	6175/0/236	3125/7/202
Goodness-of-fit on F^2	1.108	1.039	1.073
Final R indexes [I>=2σ (I)]	R_1 = 0.0225, wR_2 = 0.0491	R_1 = 0.0260, wR_2 = 0.0577	R_1 = 0.0155, wR_2 = 0.0363
Final R indexes [all data]	R_1 = 0.0269, wR_2 = 0.0510	R_1 = 0.0360, wR_2 = 0.0601	R_1 = 0.0166, wR_2 = 0.0368
Largest diff. peak/hole / e Å^{-3}	1.41/-1.32	2.86/-1.24	0.71/-0.88

***Table S4** Crystal data and structure refinement for the symmetric complexes **PtL^{1symMe2}Cl** and **PtL^{4sym}Cl**.*

	**PtL1^{symMe2}Cl**	**PtL^{4sym}Cl**
Identification code	21srv126	19srv346
Empirical formula	$C_{27}H_{23}ClN_2OPt$	$C_{18}H_9ClF_6N_2Pt$
Formula weight	622.01	597.81
Temperature/K	120.0	120.0
Crystal system	monoclinic	orthorhombic
Space group	$P2_1/c$	Aea2
a/Å	11.0717(5)	21.7303(10)
b/Å	9.4934(5)	12.4178(5)
c/Å	20.5787(10)	25.0638(11)
α/°	90	90
β/°	98.465(2)	90
γ/°	90	90
Volume/Å^3	2139.42(18)	6763.3(5)
Z	4	16
ρ_{calc}g/cm^3	1.931	2.348
μ/mm^{-1}	6.707	8.526
F(000)	1208.0	4480.0
Crystal size/mm^3	0.32 × 0.08 × 0.06	0.2 × 0.19 × 0.14
Radiation	MoKα (λ = 0.71073)	Mo Kα (λ = 0.71073)
2Θ range for data collection/°	5.048 to 60	4.112 to 60.984
Index ranges	$-15 \leq h \leq 15$, $-13 \leq k \leq 13$, $-28 \leq l \leq 28$	$-31 \leq h \leq 31$, $-17 \leq k \leq 17$, $-35 \leq l \leq 35$
Reflections collected	50001	55982
Independent reflections	6221 [R_{int} = 0.0353, R_{sigma} = 0.0201]	10232 [R_{int} = 0.0336, R_{sigma} = 0.0251]
Data/restraints/parameters	6221/6/293	10232/1/505
Goodness-of-fit on F^2	1.108	1.047
Final R indexes [I>=2σ (I)]	R_1 = 0.0219, wR_2 = 0.0479	R_1 = 0.0272, wR_2 = 0.0651
Final R indexes [all data]	R_1 = 0.0243, wR_2 = 0.0488	R_1 = 0.0303, wR_2 = 0.0665
Largest diff. peak/hole / e Å^{-3}	2.94/-0.78	0.70/-1.13

Section 3 Additional absorption and emission spectra

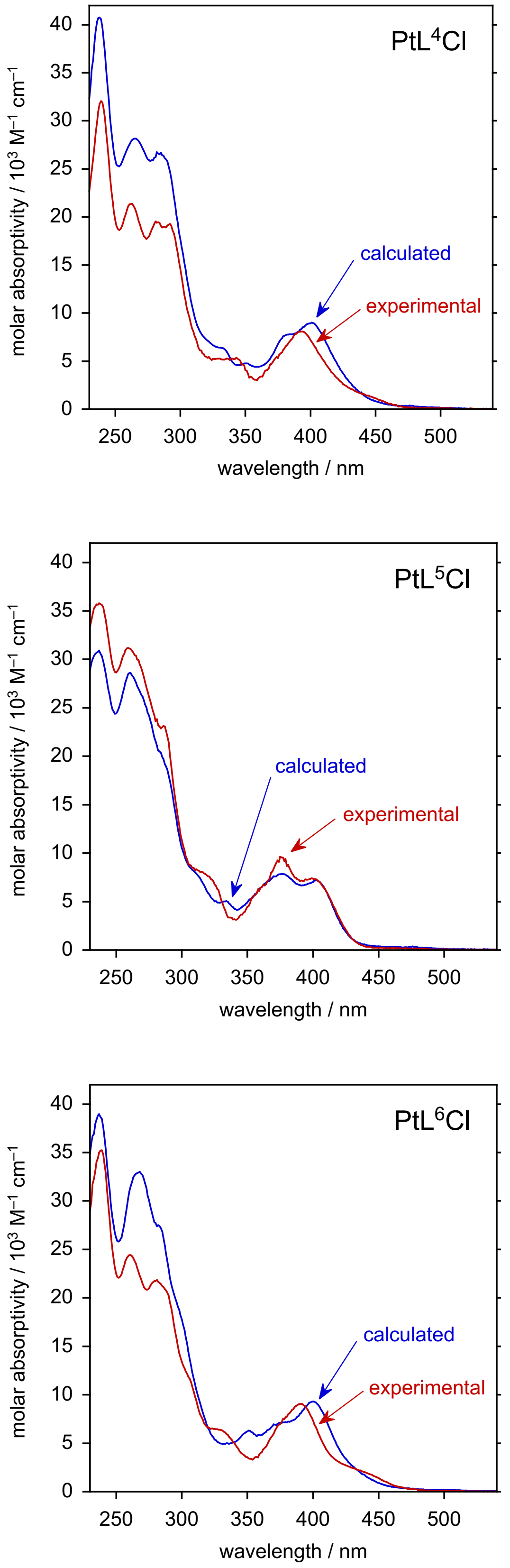

Figure S8 *Experimental absorption spectra of PtL^{n}Cl in CH_2Cl_2 (red) overlaid with spectra calculated as the weighted average of those of PtL^{nsym}Cl and Pt(dpyb)Cl {n = 4, top; n= 5, middle; n= 6, bottom}.*

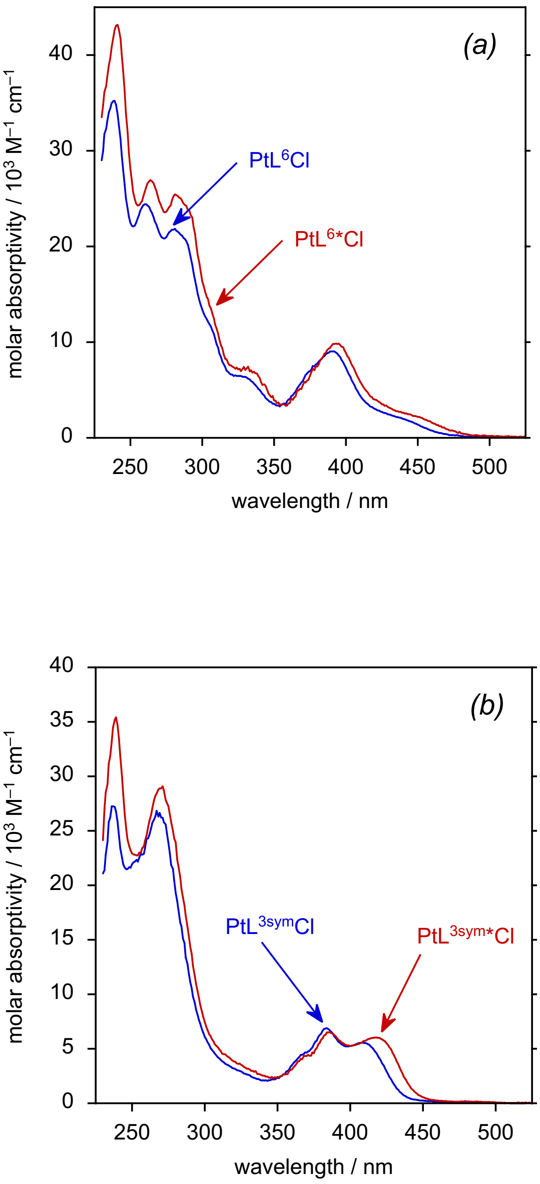

Figure S9 *Overlay of the absorption spectra in* CH_2Cl_2 *at 295 K of (a)* PtL^6Cl *and its t-butyl derivative* $PtL^6{*}Cl$ *(blue and red lines respectively); (b)* $PtL^{3sym}Cl$ *and its t-butyl derivative* $PtL^{3sym}{*}Cl$ *(blue and red lines respectively).*

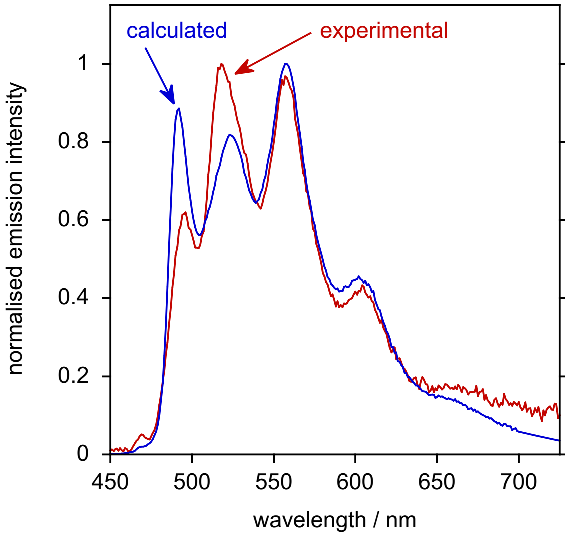

Figure S10 *A "simulated spectrum" of PtL*[1]*Cl (blue line), calculated as the average of the spectra of PtL*[1sym]*Cl and Pt(dpyb)Cl in* CH_2Cl_2 *at 295 K, together with the experimental spectrum of PtL*[1]*Cl under the same conditions (red line). Note that the match is no better with ≠ 50/50 ratio.*

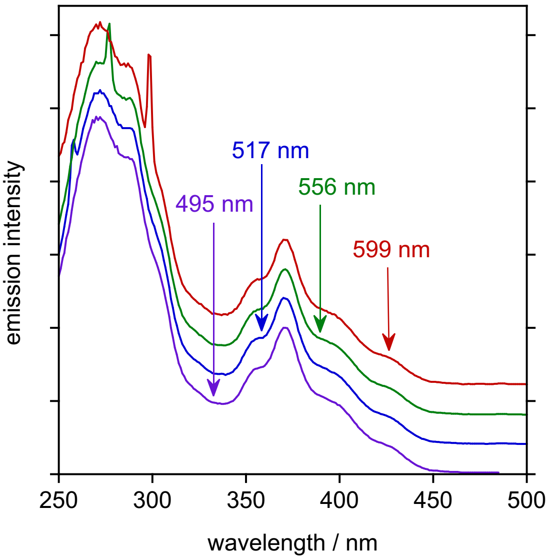

Figure S11 *Excitation spectra of PtL*[1]*Cl registered at the emission wavelengths indicated, highlighting that the excited state that emits at 495 nm has the same profile as that which gives rise to the longer wavelength bands. The spectra have been normalised at 372 nm and offset relative to one another for clarity. (The sharp peaks at 258, 278 and 299 nm in the spectra recorded at* λ_{em} = *517, 556 and 599 nm, respectively, are the second-order bands of the scattered excitation light).*

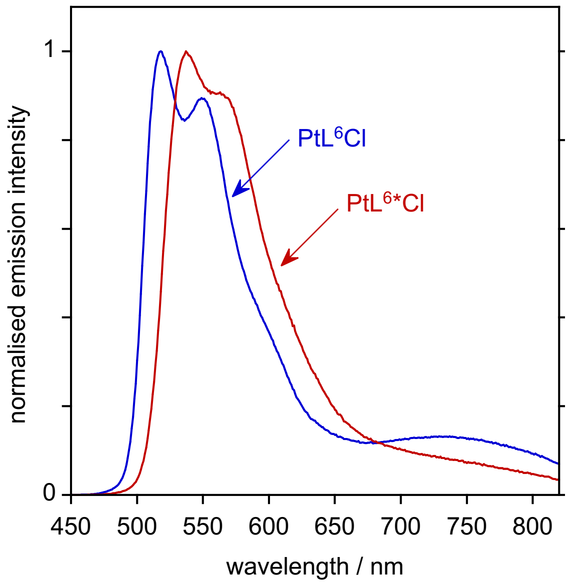

Figure S12 *Normalised emission spectra of PtL^{6}Cl and its t-butyl derivative PtL^6*Cl in concentrated solution in CH_2Cl_2 (2×10^{-4} M) at 295 K (blue and red lines respectively).*

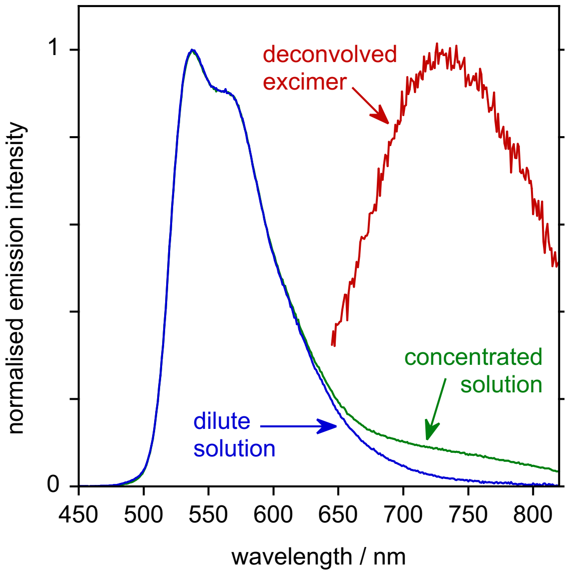

Figure S13 *Normalised emission spectra of PtL^6*Cl in concentrated and dilute solutions in CH_2Cl_2 at 295 K (blue and green lines respectively), and the spectrum of the excimer estimated by subtraction of the latter from the former (red line).*

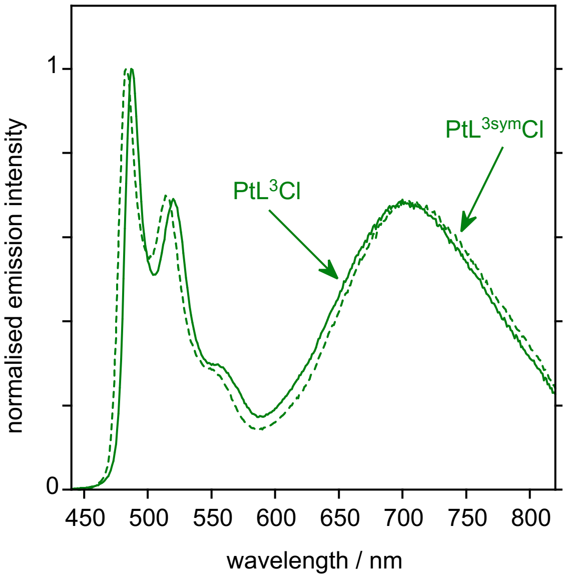

Figure S14 *Emission spectra of PtL^{3}Cl (solid line) and PtL^{3sym}Cl (dashed line) at elevated concentration in CH_2Cl_2 at 295 K.*

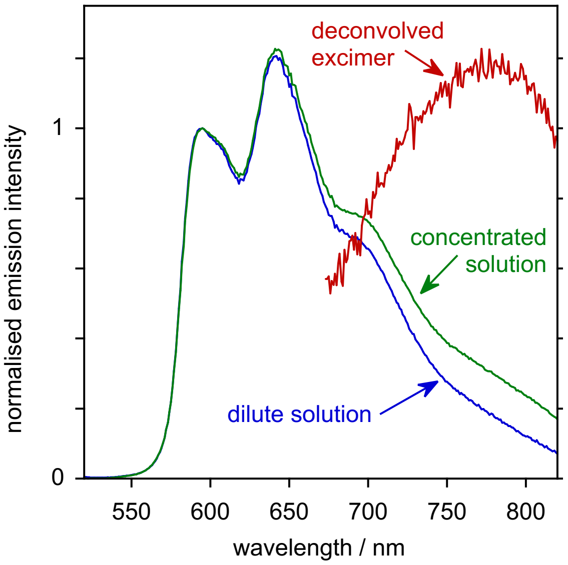

Figure S15 *Emission spectra of PtL^{2}Cl in concentrated and dilute solutions in CH_2Cl_2 at 295 K (blue and green lines respectively), normalised at 595 nm, and the spectrum of the excimer estimated by subtraction of the latter from the former (red line).*

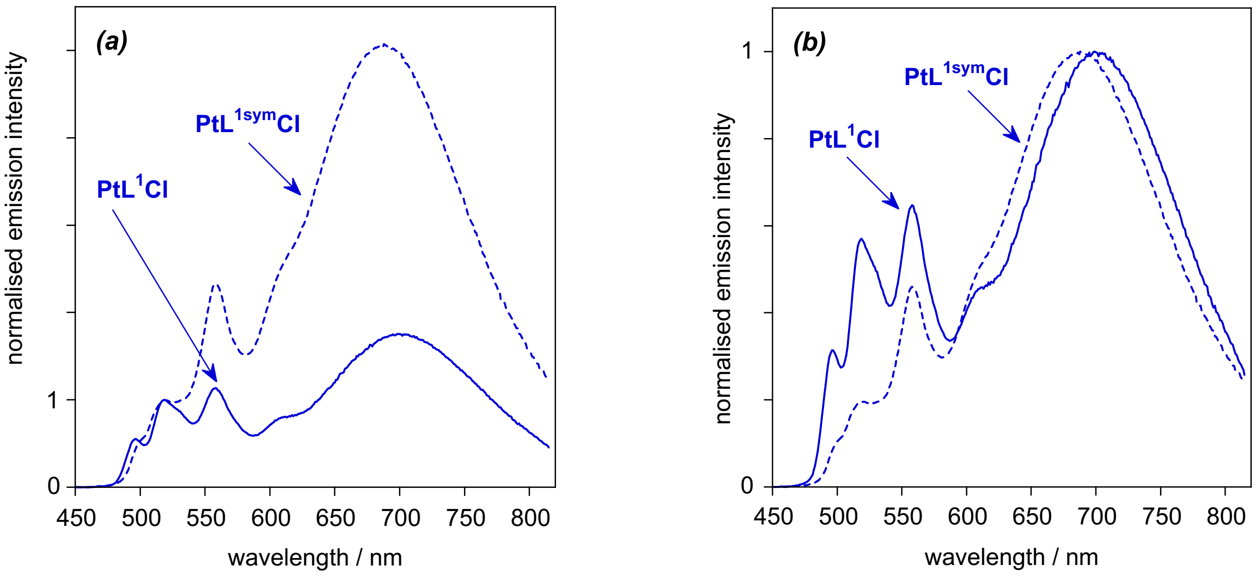

Figure S16 *Emission spectra of PtL*^{1}*Cl (solid line) and PtL*^{1sym}*Cl (dashed line) at a concentration of 2.4* $\times 10^{-5}$ *M in* CH_2Cl_2 *at 295 K: (a) normalised to the unimolecular emission band at 519 nm, and (b) normalised to the excimer emission band.*

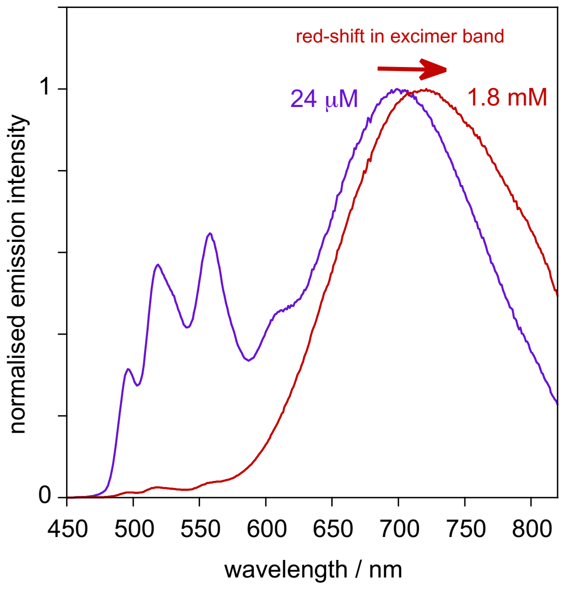

Figure S17 *Emission spectra of PtL*^{1}*Cl at concentrations of 24 μM and 1.8 mM in* CH_2Cl_2 *at 295 K – as in Fig. 9 of the main text – but normalised here to* λ_{max} *of the excimer band, to show the small yet distinctive red-shift at elevated concentrations, ostensibly from higher-order aggregates.*

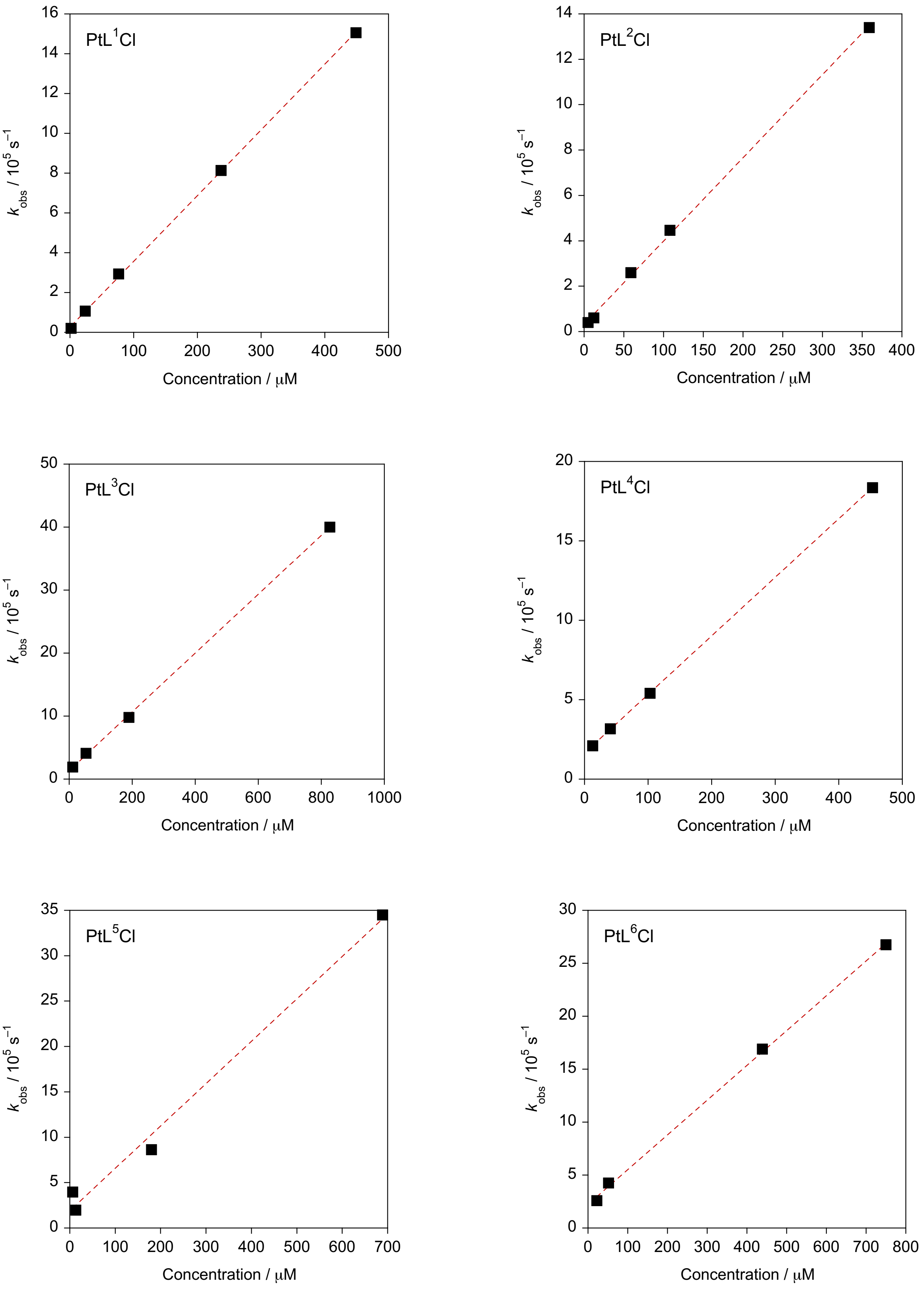

Figure S18 *Plots of the 1st order rate constant of emission decay k_{obs} as a function of concentration for the non-symmetric complexes PtL$^{1-6}$Cl in deoxygenated CH_2Cl_2 at 295 K. The red dashed line in each plot is the least-squares best linear fit to the data points.*

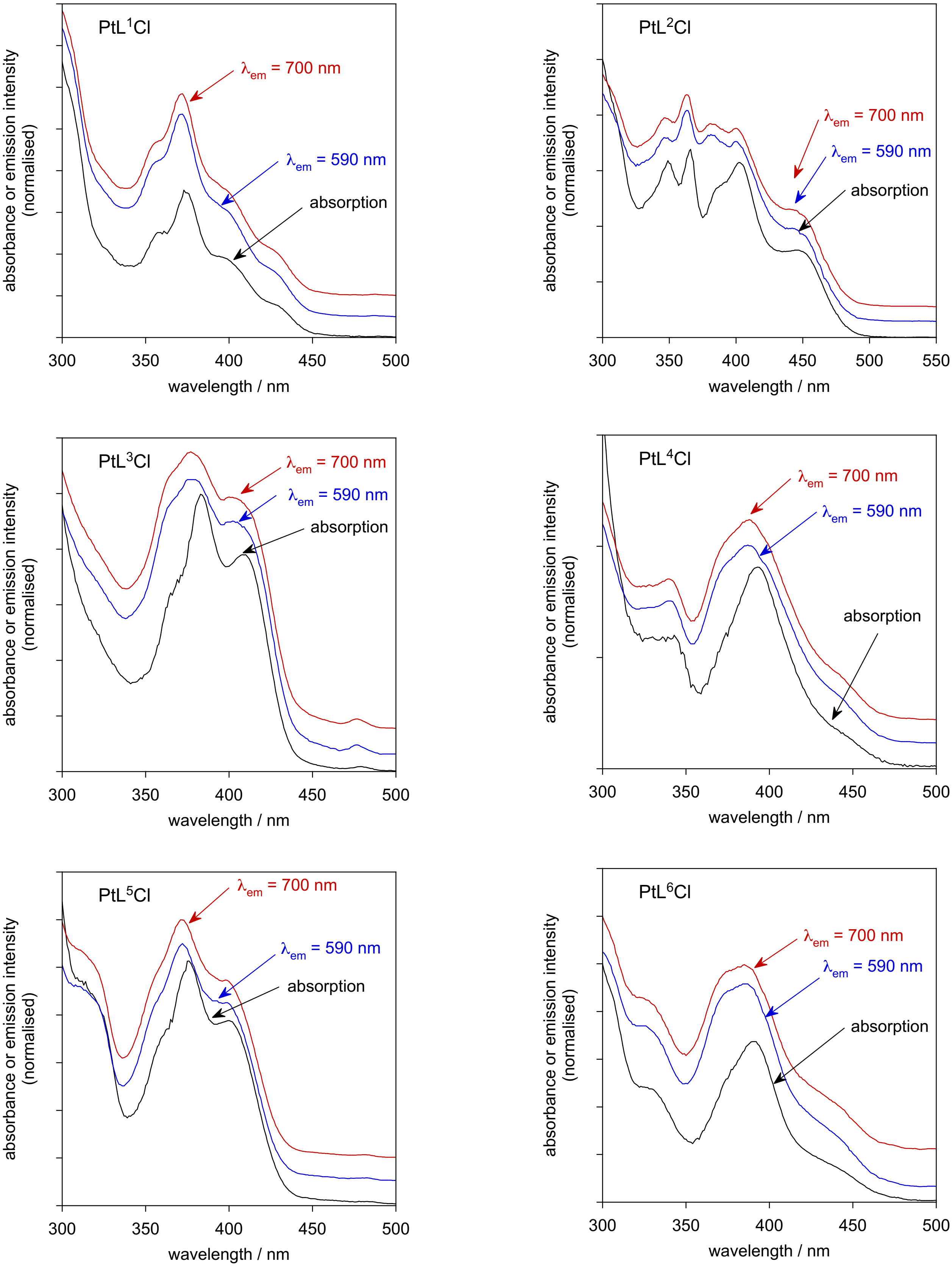

Figure S19 *Normalised excitation spectra of concentrated solutions of $PtL^{1-6}Cl$ in CH_2Cl_2 (approx. 10^{-4} M) registered for the unimolecular and excimer emission bands at the wavelengths indicated (blue and red lines respectively), showing them to be essential identical in each case. The absorption spectra are shown for reference though an exact match with the excitation spectra is not anticipated at these elevated concentrations, due to the inner filter effect at high absorbances.*

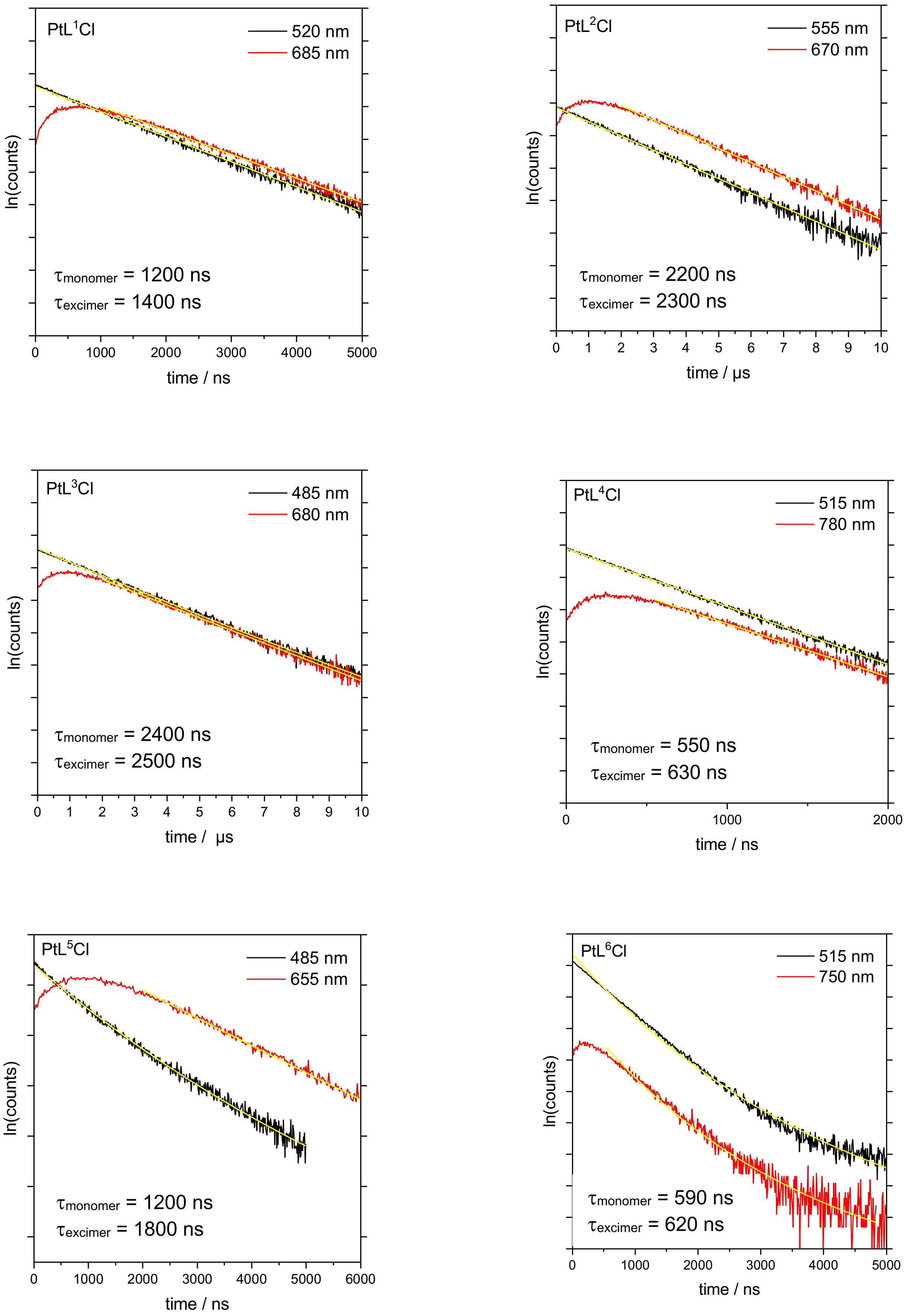

***Figure S20** Decays of the unimolecular and excimer emission (black and red lines respectively), registered at the wavelengths indicated, for $PtL^{1-6}Cl$ in deoxygenated CH_2Cl_2 (approx. 10^{-4} M), with mono-exponential tail fits shown in yellow and corresponding lifetimes indicated.*

Section 4 Calculations using Density Functional Theory

Density functional theory (DFT) and time-dependent density functional theory (TDDFT) simulations with Tamm-Dancoff approximation (TDA) were performed on the complexes using the ORCA 5.0.3 quantum chemistry software.[1–4] Molecular orbital iso surfaces were visualised using ChimeraX-1.4[5,6] or Avogadro 1.2.0.[7,8] Geometry optimisations of the complexes in the ground state were performed using B3LYP[9,10]/def2-SVP.[11] Triplet excited state (T_1) geometries were performed similarly, but also using the BP86[12]/def2-SVP[11] with the RI keyword, and def2-SVP/C[13] and def2/J[14] auxiliary basis sets. Single-point energy calculations were performed at the B3LYP/def2-SVP level of theory with the aid of the RIJCOSX[15,16] approximation and using CPCM for the solvent CH_2Cl_2 in all cases. All calculations were performed using very tight geometry and SCF convergence criteria, and using the atom-pairwise dispersion correction with the Becke-Johnson damping scheme (D3BJ).[17,18] Frequency calculations were used to confirm that the respective optimised geometries were energy minima.

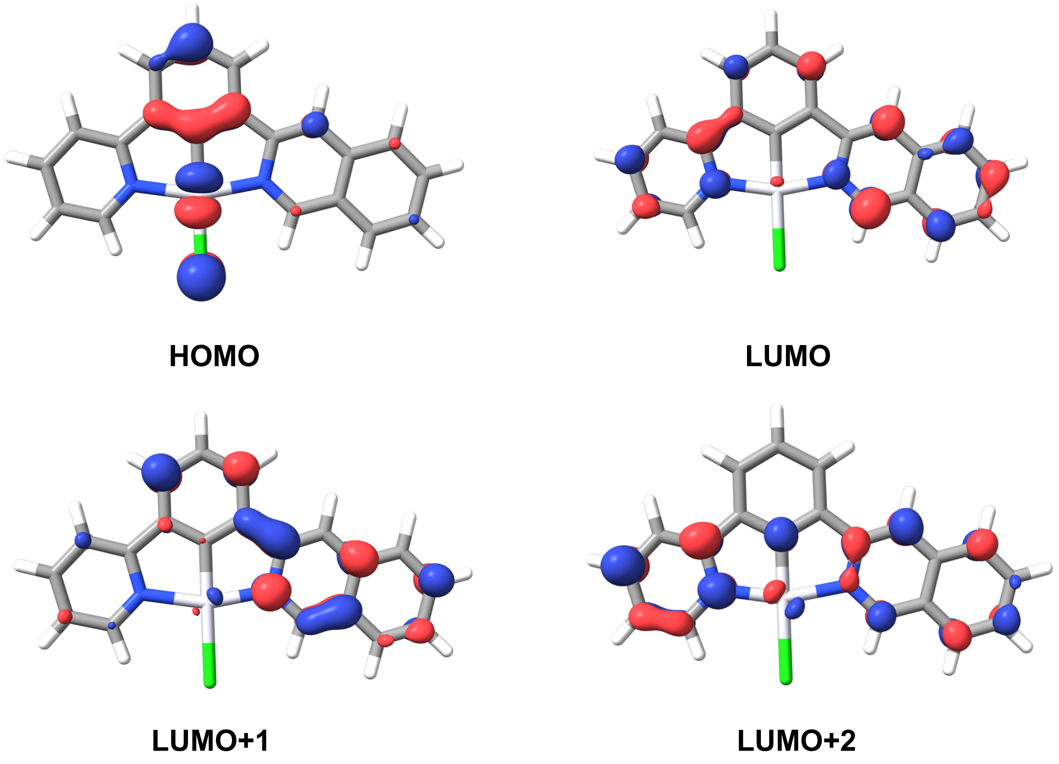

Figure S21 *Frontier orbital plots for PtL^{1}Cl at the S_0 geometry calculated using B3LYP/def2-SVP and CPCM for CH_2Cl_2. The lowest-energy singlet transition has almost exclusively HOMO $\rightarrow$ LUMO character; the second-lowest is predominantly HOMO $\rightarrow$ LUMO+1; and the third-lowest is predominantly HOMO $\rightarrow$ LUMO+2.*

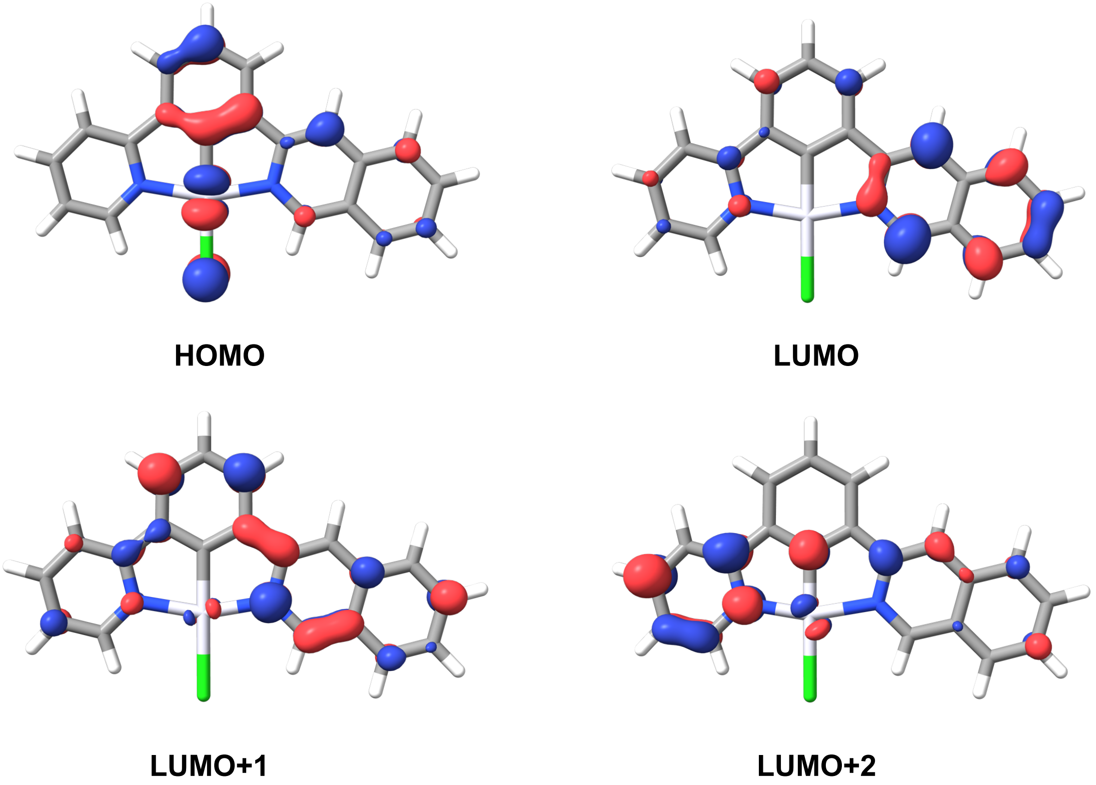

Figure S22 *Frontier orbital plots for PtL[1]Cl at the T_1 geometry calculated using B3LYP/def2-SVP and CPCM for CH_2Cl_2. The lowest-energy triplet transition has predominantly HOMO $\rightarrow$ LUMO character whilst the main component of the second-lowest triplet transition is HOMO $\rightarrow$ LUMO+2.*

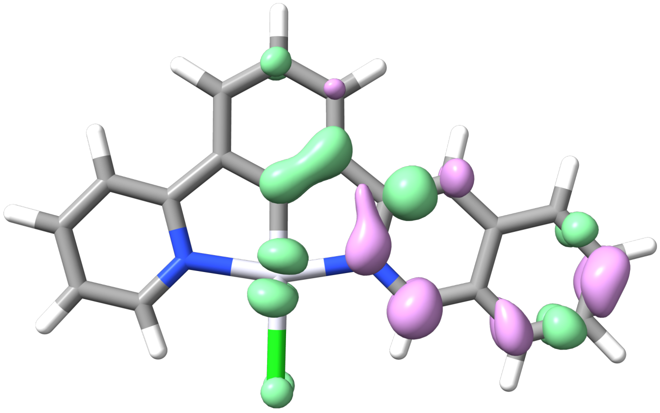

Figure S23 *Density difference plot for the T_1 state of PtL[1]Cl calculated at the T_1 geometry using B3LYP/def2-SVP and CPCM for CH_2Cl_2. Green and pink represent zones of depletion and augmentation of electron density, respectively.*

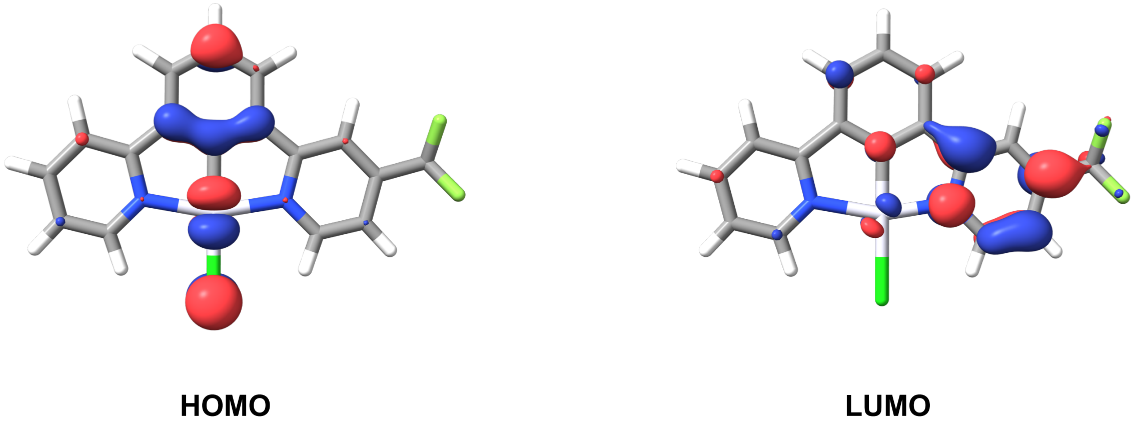

Figure S24 *Frontier orbital plots for PtL^4Cl (as a representative example of the bis-pyridyl complexes) at the S_0 geometry, calculated using B3LYP/def2-SVP and CPCM for CH_2Cl_2. The lowest-energy singlet and triplet transitions have predominant HOMO → LUMO character. See also reference 19 for further examples.*

Section 5 ^{1}H and ^{13}C NMR spectra

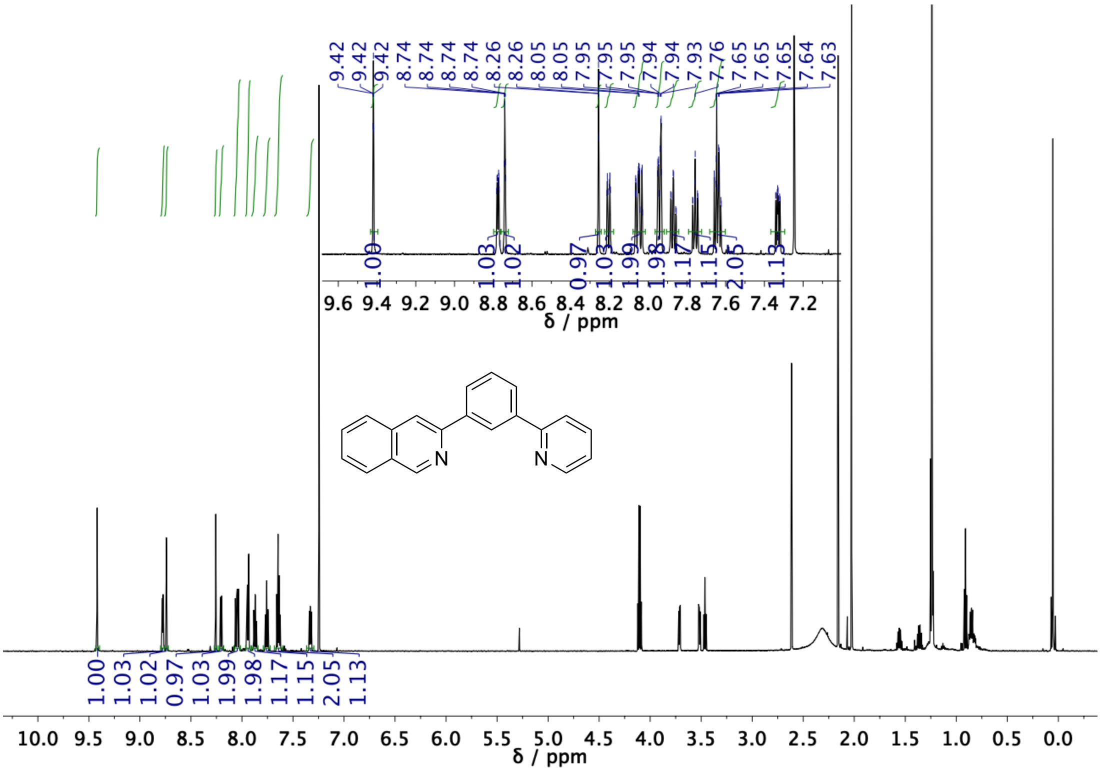

Figure S25 *^{1}H NMR spectrum of HL^{1} in $CDCl_3$.*

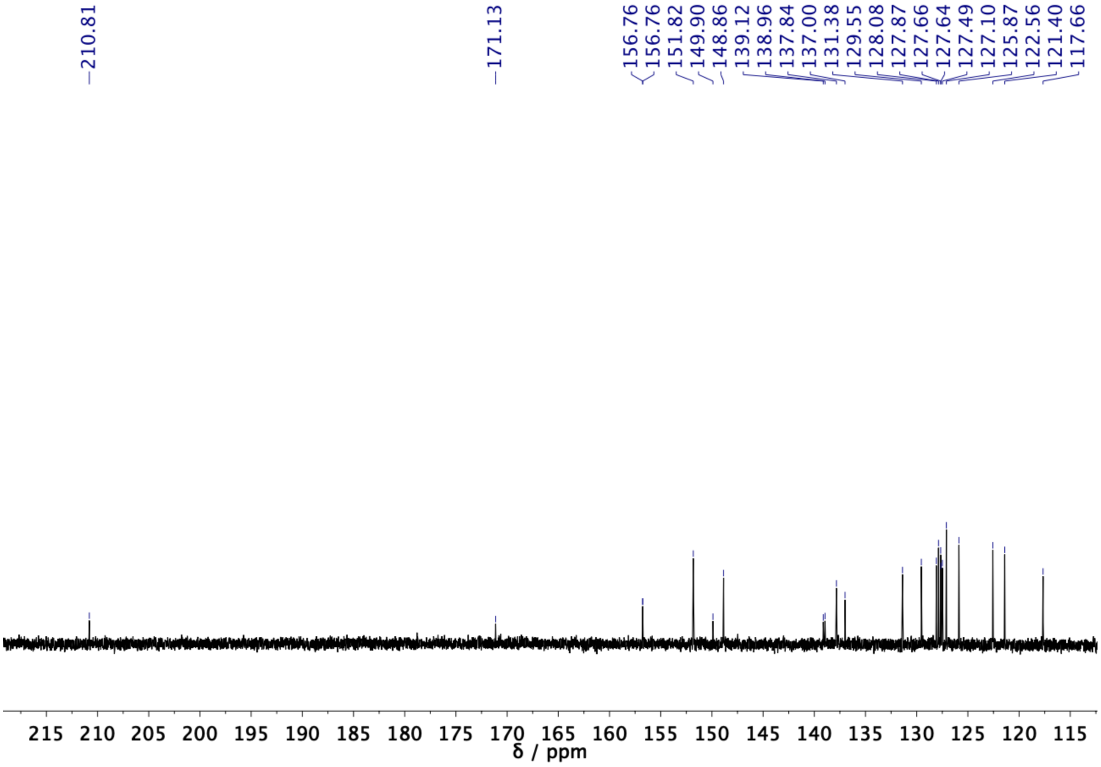

Figure S26 *^{13}C NMR spectrum of HL^{1} in $CDCl_3$.*

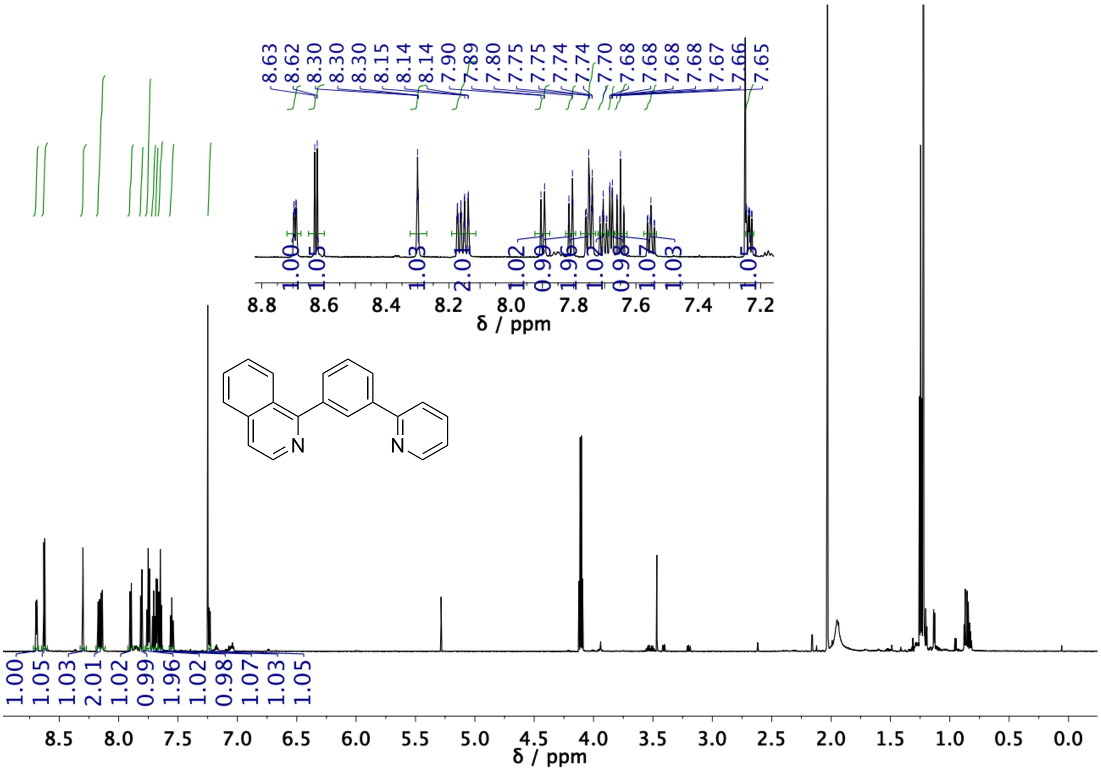

Figure S27 *¹H NMR spectrum of HL² in CDCl₃.*

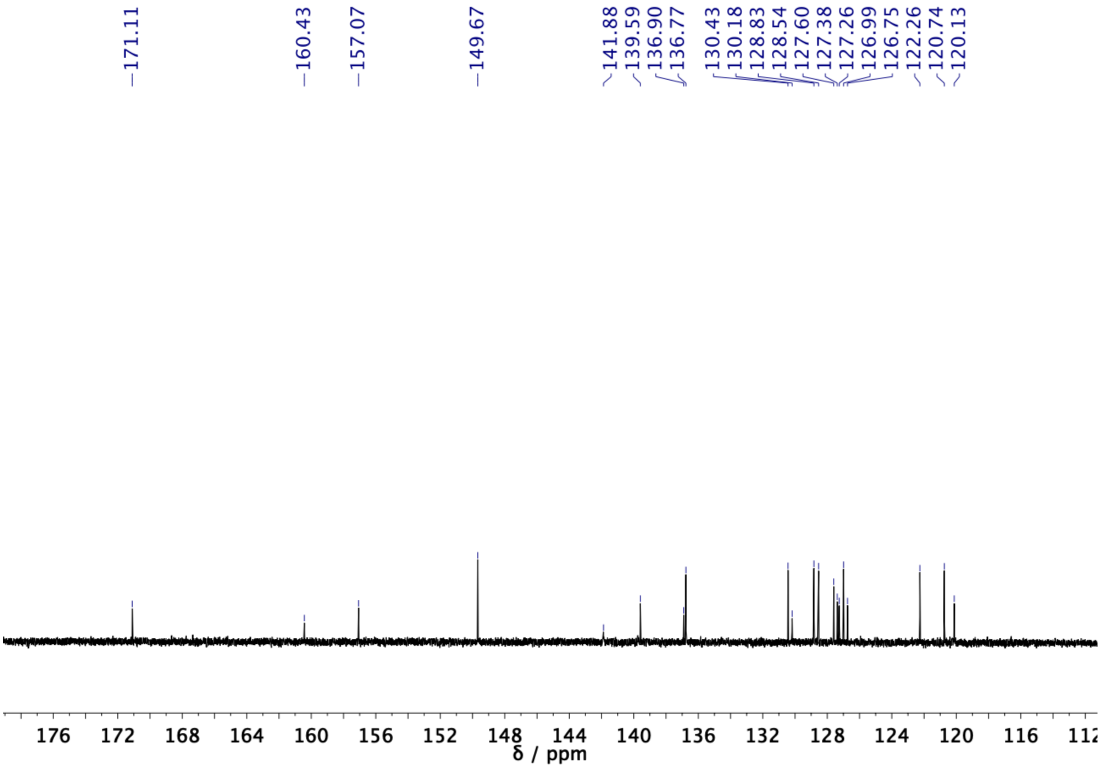

Figure S28 *¹³C NMR spectrum of HL² in CDCl₃.*

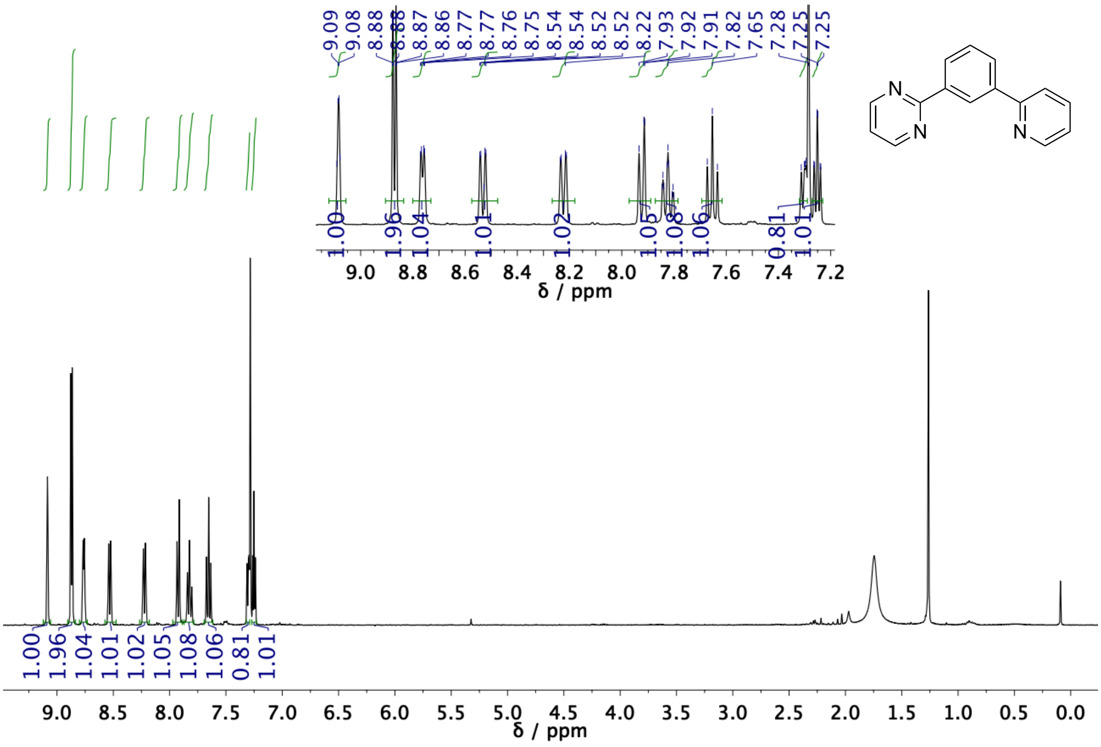

Figure S29 *^{1}H NMR spectrum of HL^{3} in $CDCl_3$.*

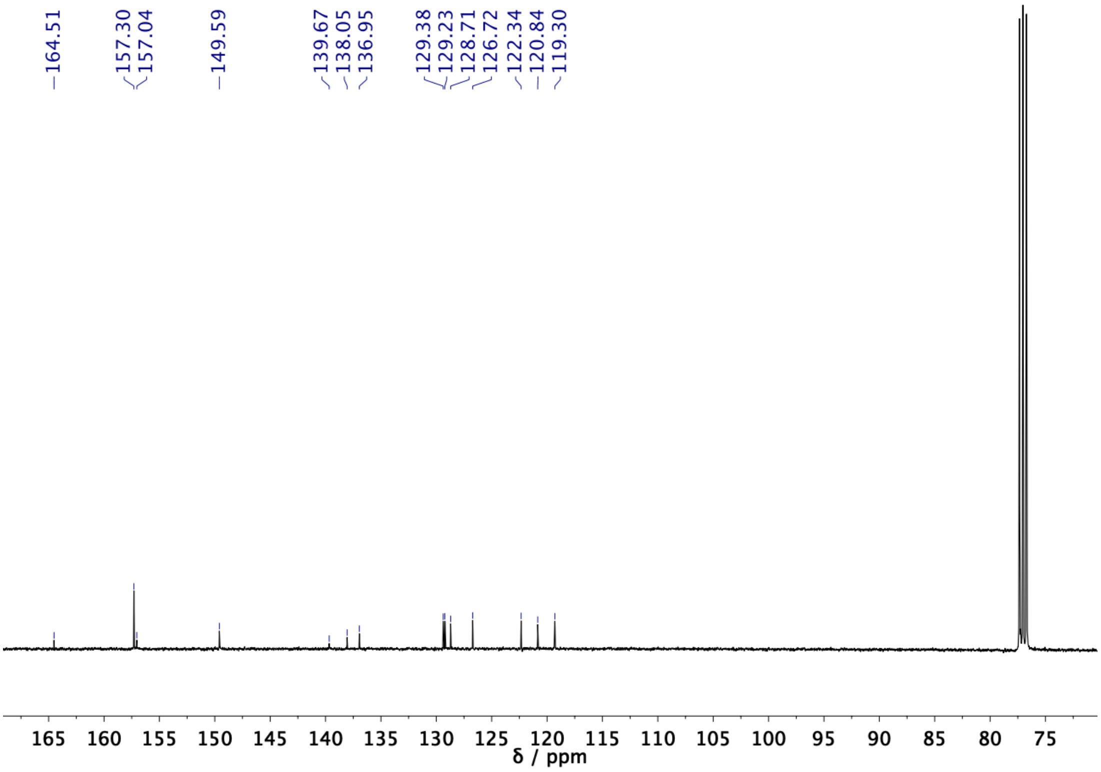

Figure S30 *^{13}C NMR spectrum of HL^{3} in $CDCl_3$.*

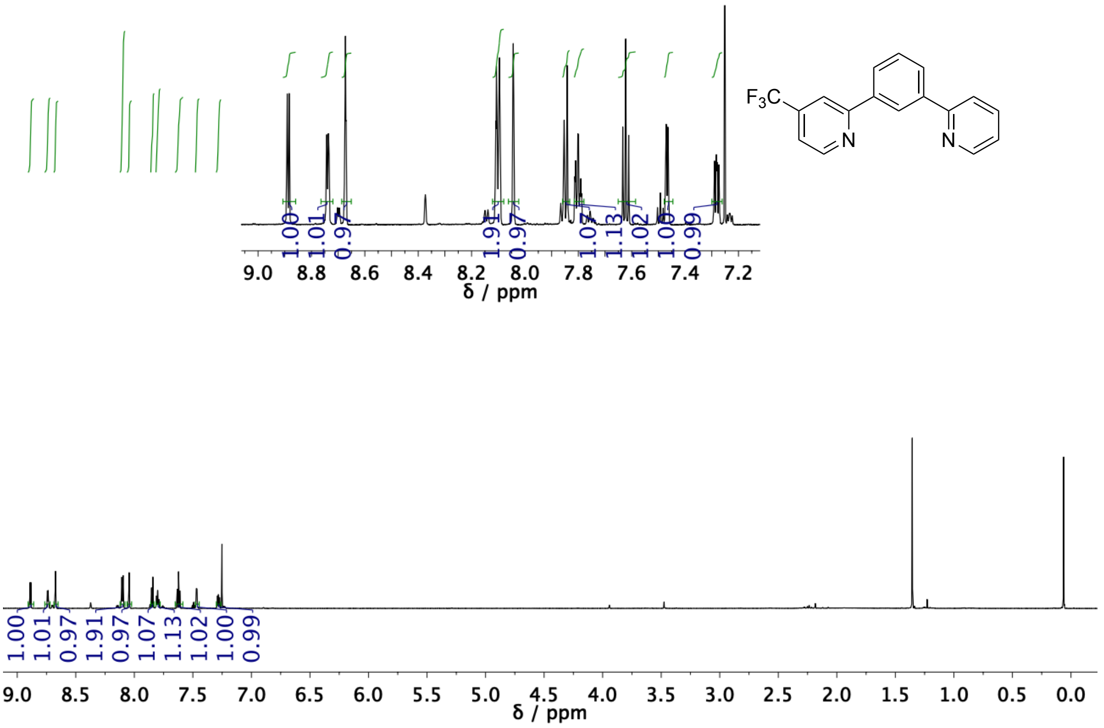

Figure S31 ^{1}H NMR spectrum of HL[4] in CDCl3.

i

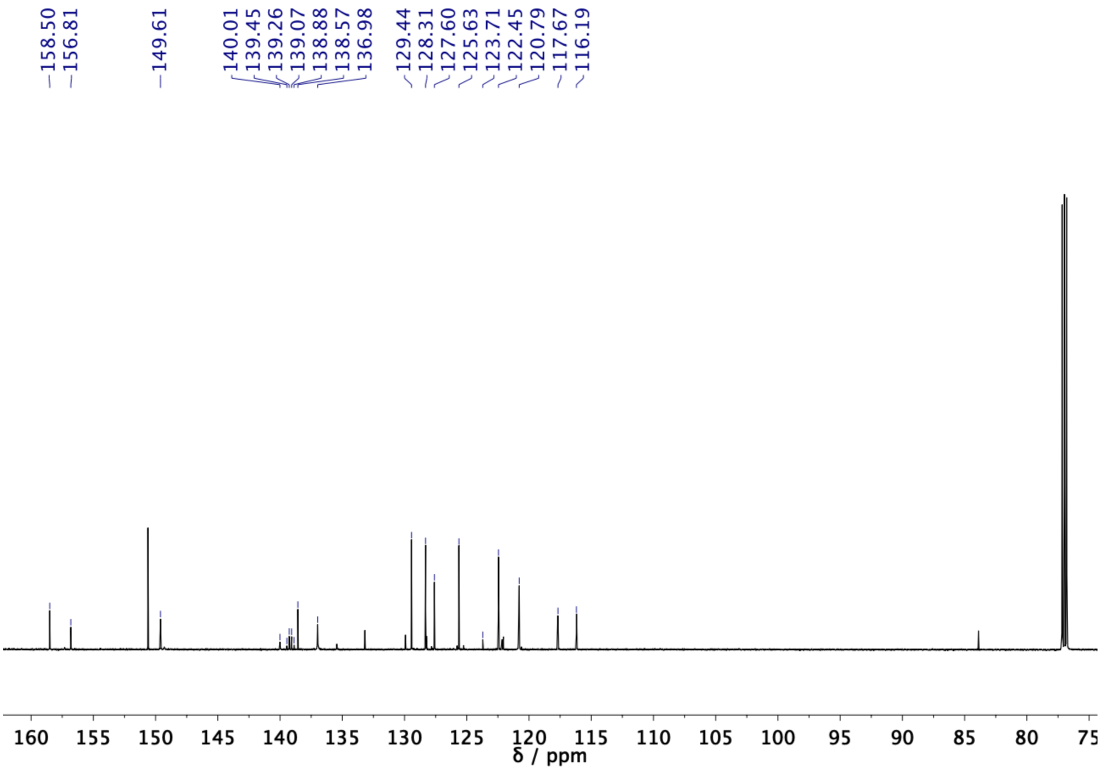

Figure S32 ^{13}C NMR spectrum of HL[4] in CDCl3.

[i] This proligand was sufficiently pure for complexation.

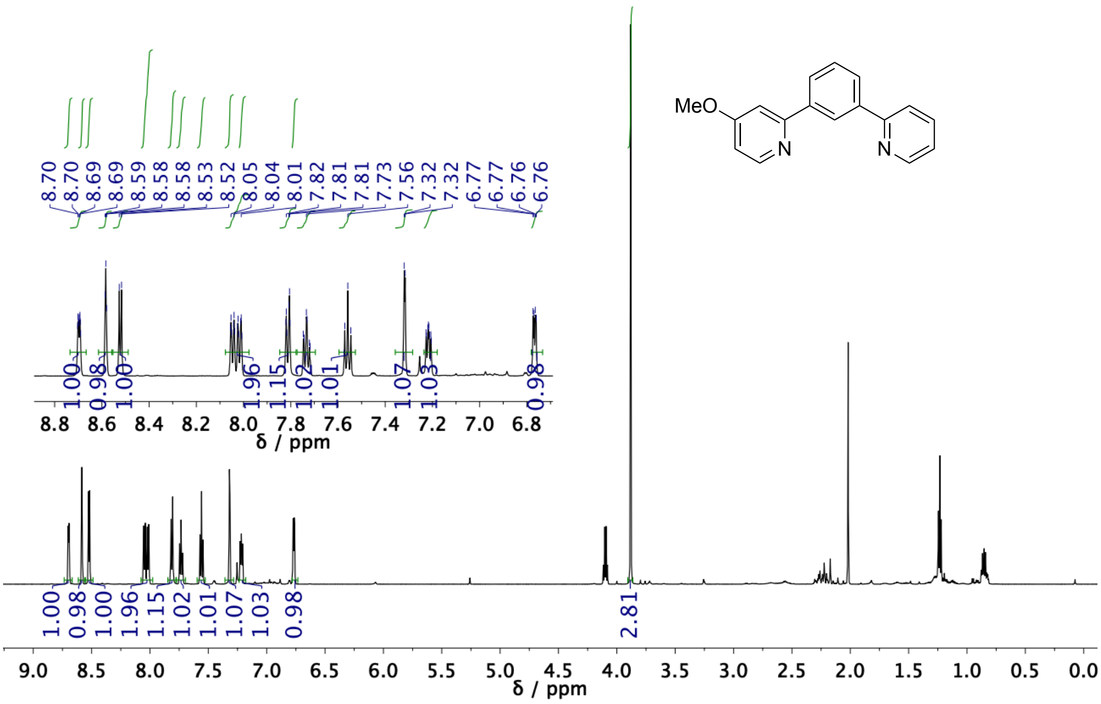

Figure S33 *^{1}H NMR spectrum of HL^{5} in $CDCl_3$.*

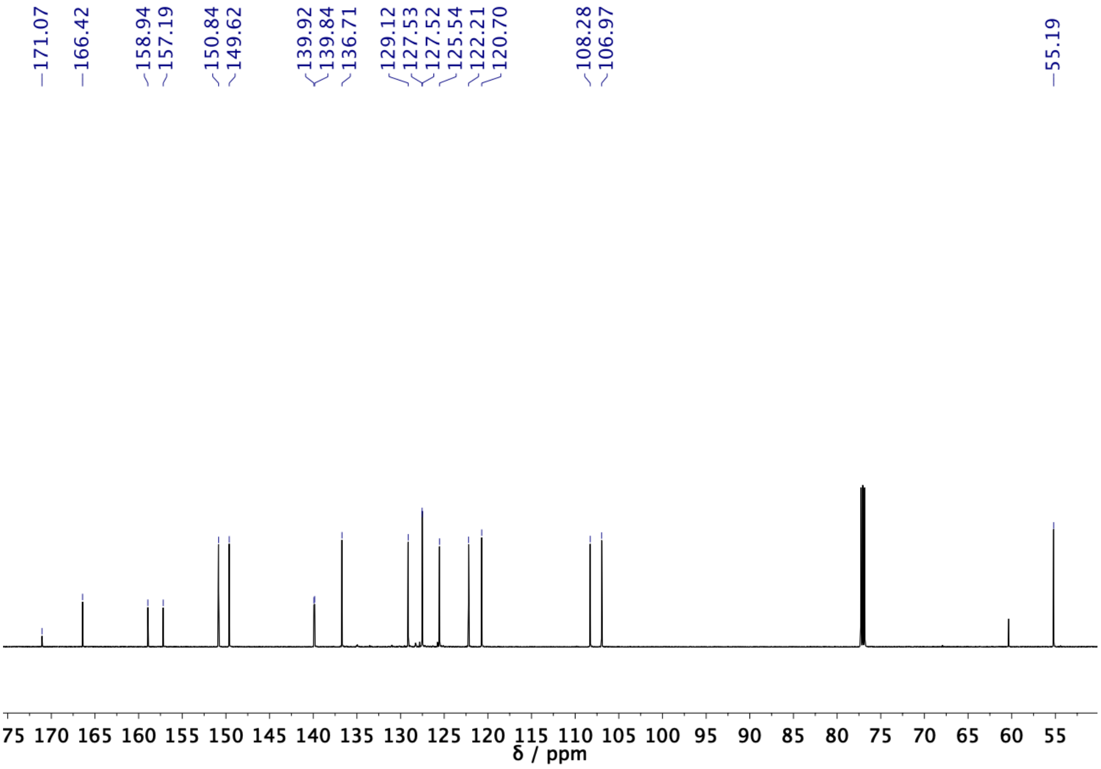

Figure S34 *^{13}C NMR spectrum of HL^{5} in $CDCl_3$.*

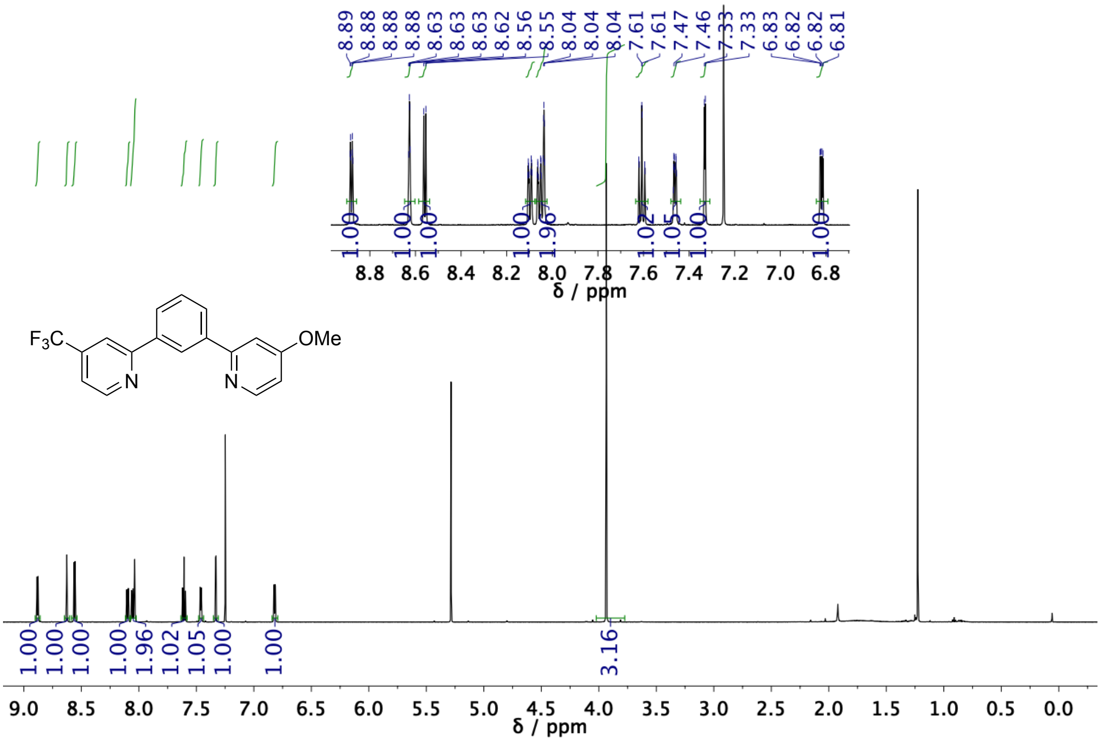

Figure S35 *^{1}H NMR spectrum of HL[6] in $CDCl_3$.*

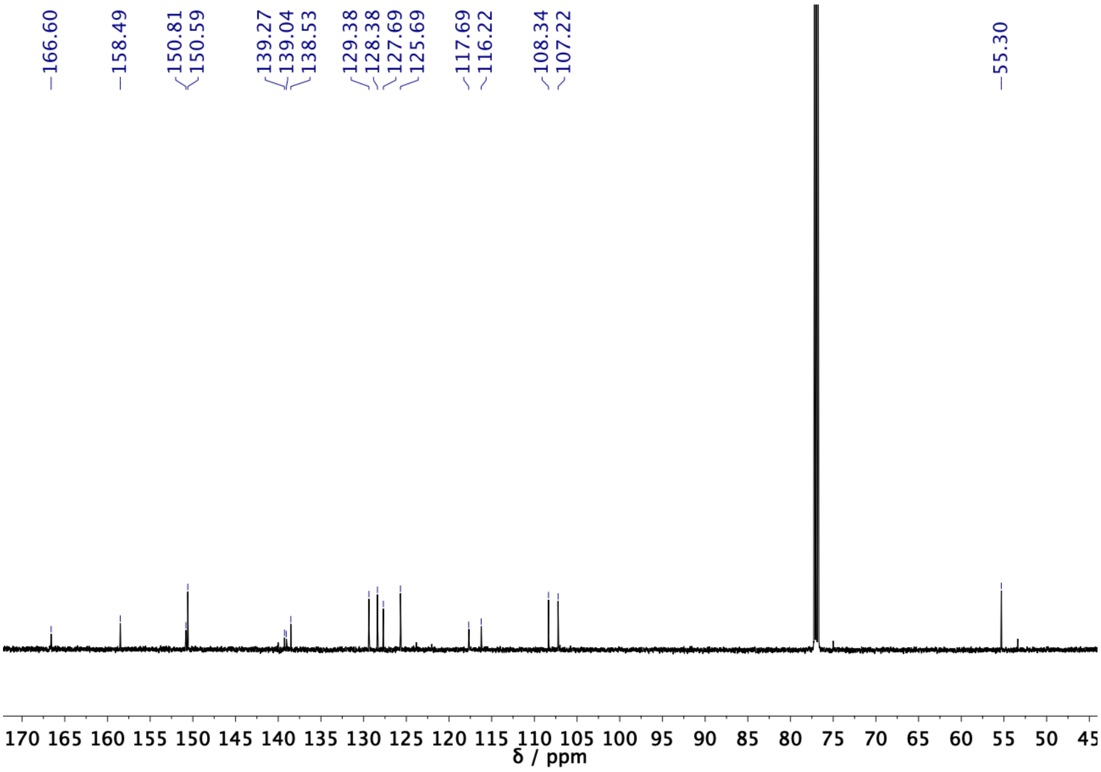

Figure S36 *^{13}C NMR spectrum of HL[6] in $CDCl_3$.*

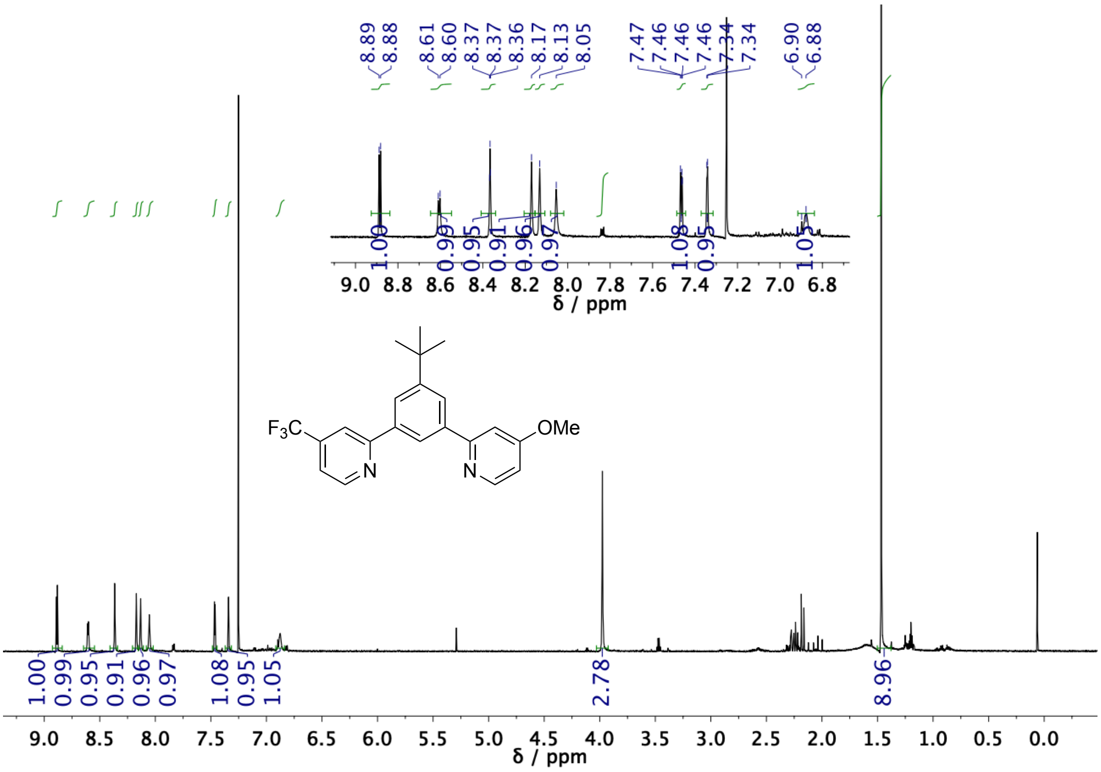

Figure S37 *^{1}H NMR spectrum of HL^{6}* in $CDCl_3$.*

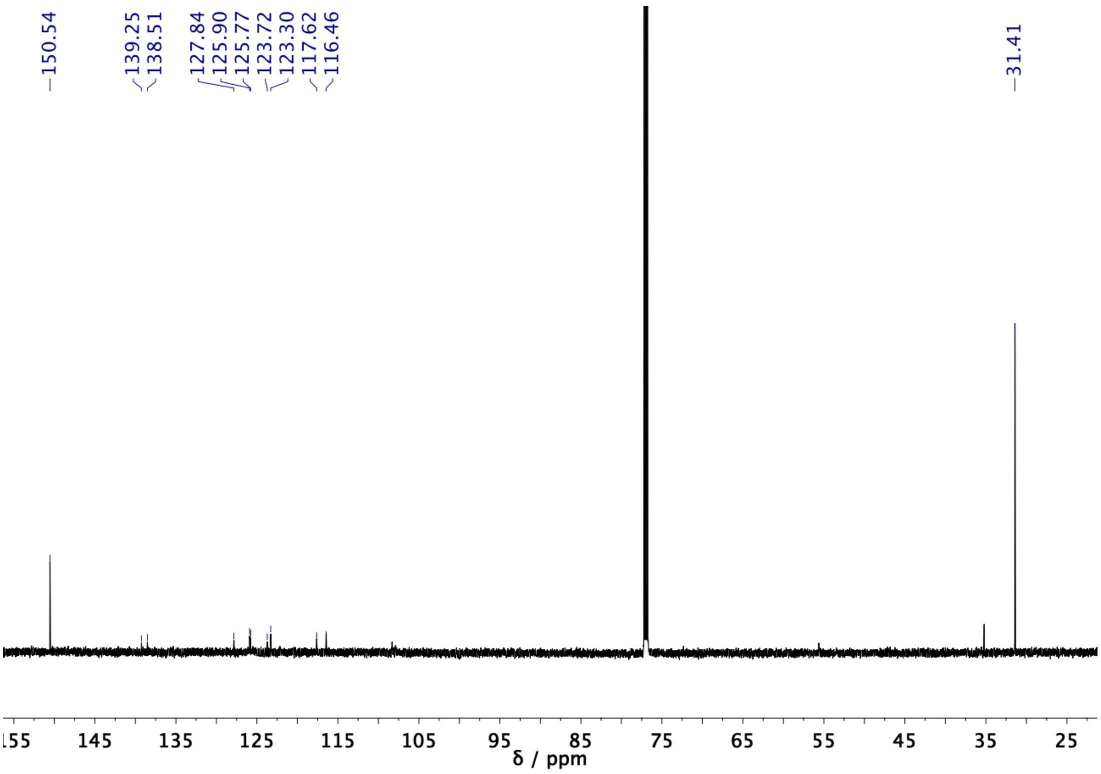

Figure S38 *^{13}C NMR spectrum of HL^{6}* in $CDCl_3$.*

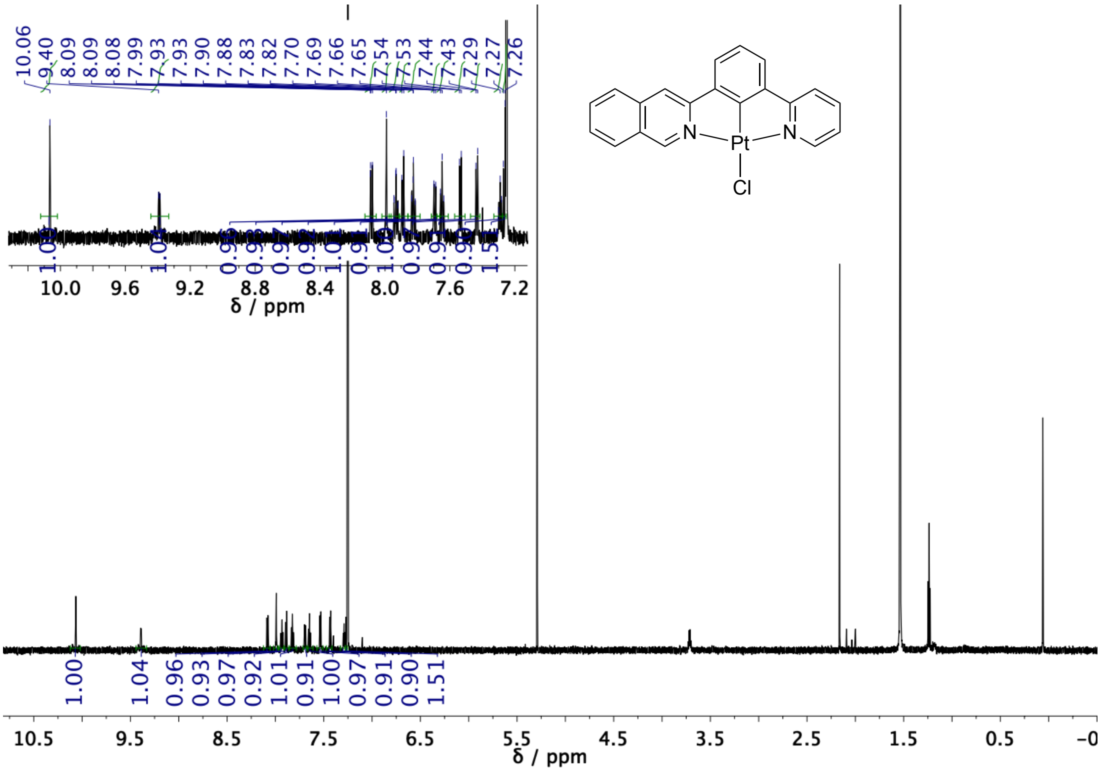

Figure S39 *¹H NMR spectrum of PtL¹Cl in CDCl₃.*

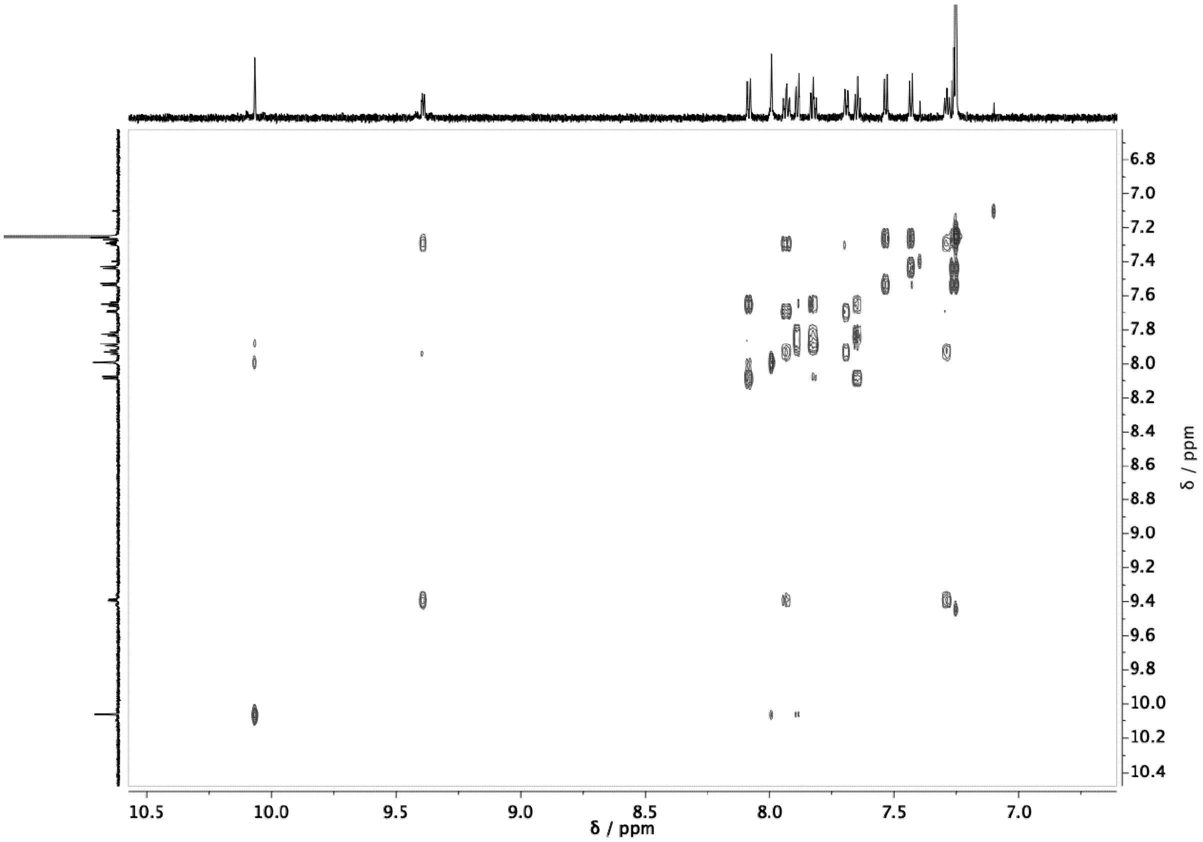

Figure S40 *COSY NMR spectrum of PtL¹Cl in CDCl₃.*

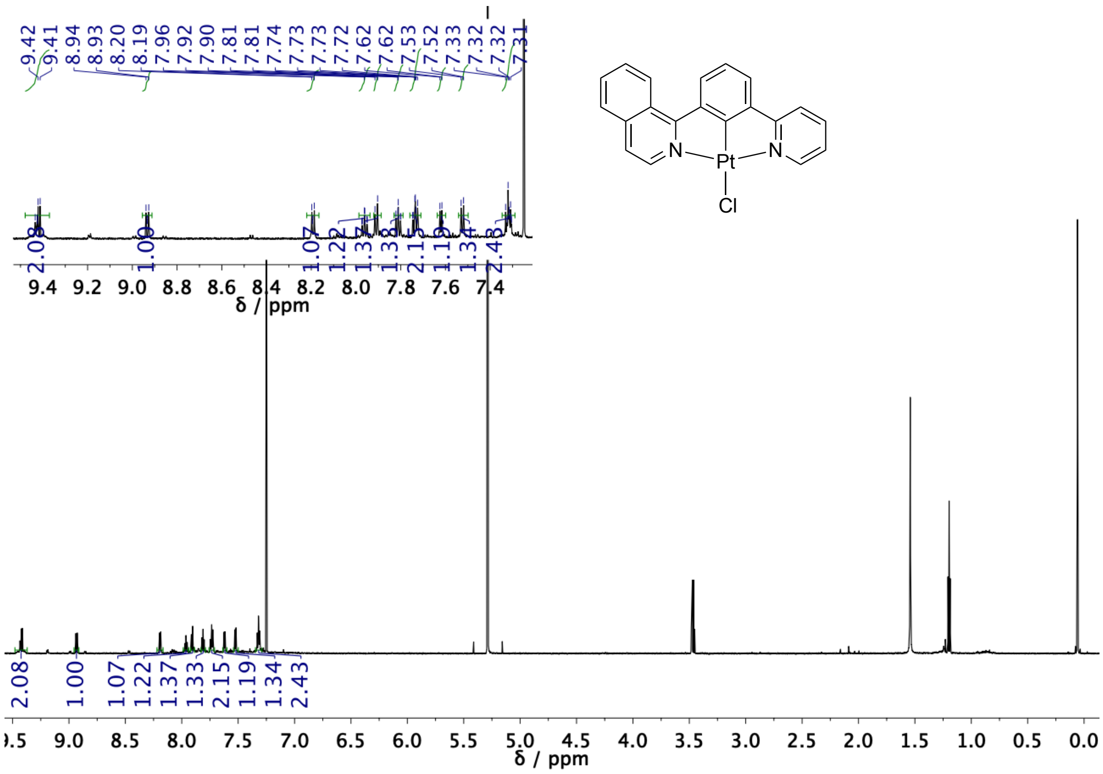

Figure S41 *^{1}H NMR spectrum of PtL[2]Cl in CDCl$_3$.*

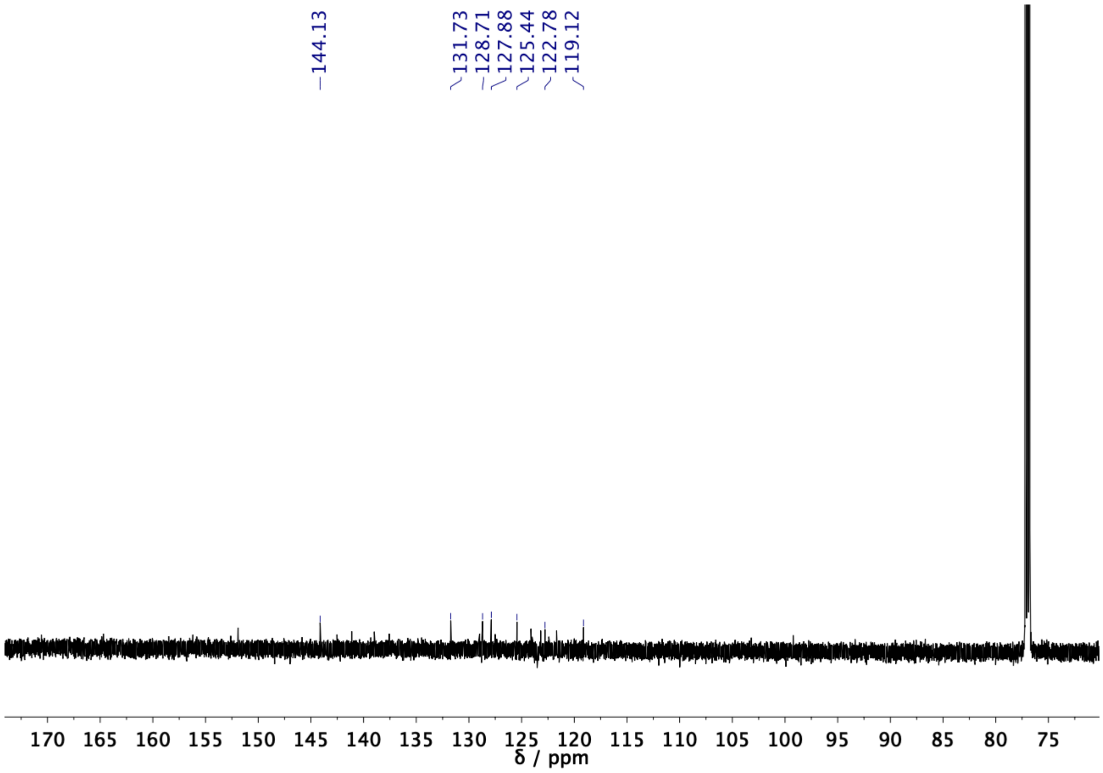

Figure S42 *^{13}C NMR spectrum of PtL[2]Cl in CDCl$_3$.*

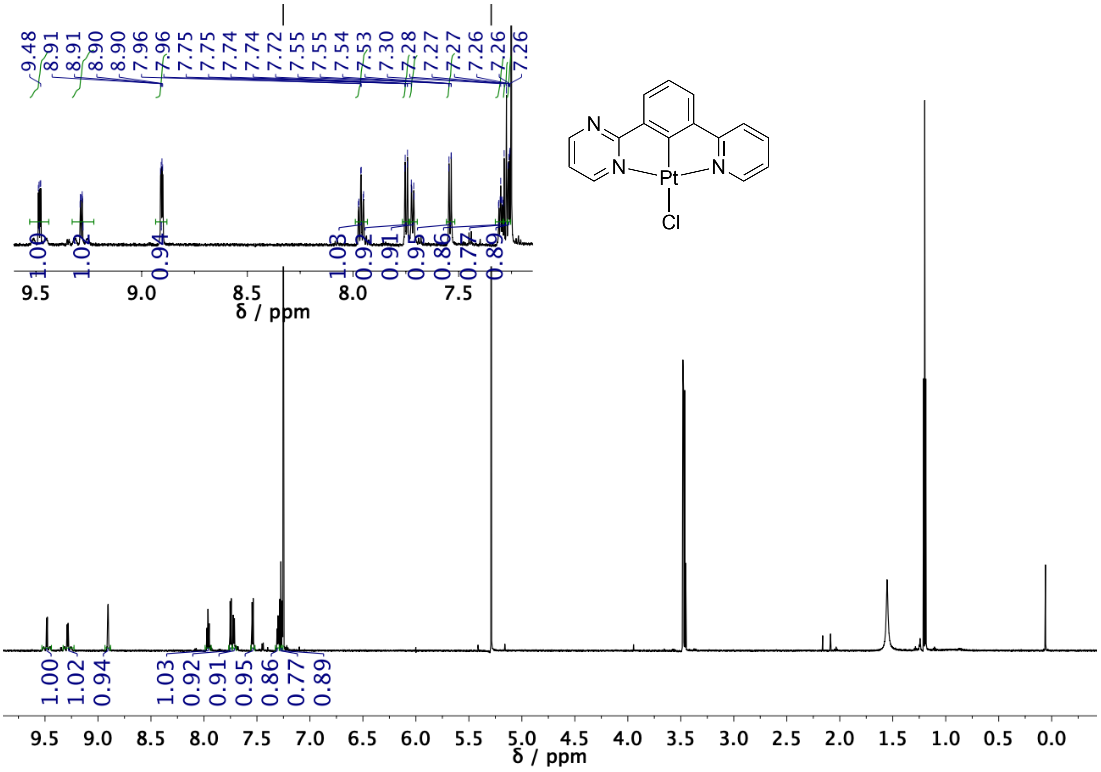

Figure S43 *^1H NMR spectrum of PtL^3Cl in $CDCl_3$.*

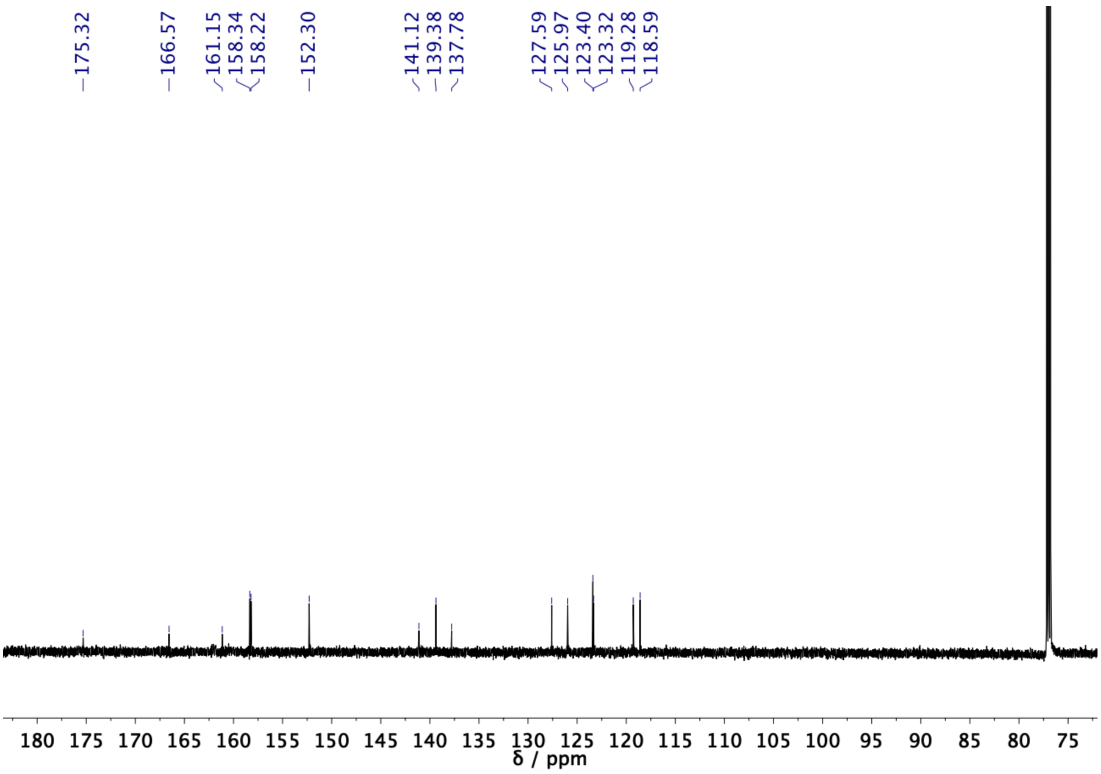

Figure S44 *^{13}C NMR spectrum of PtL^3Cl in $CDCl_3$.*

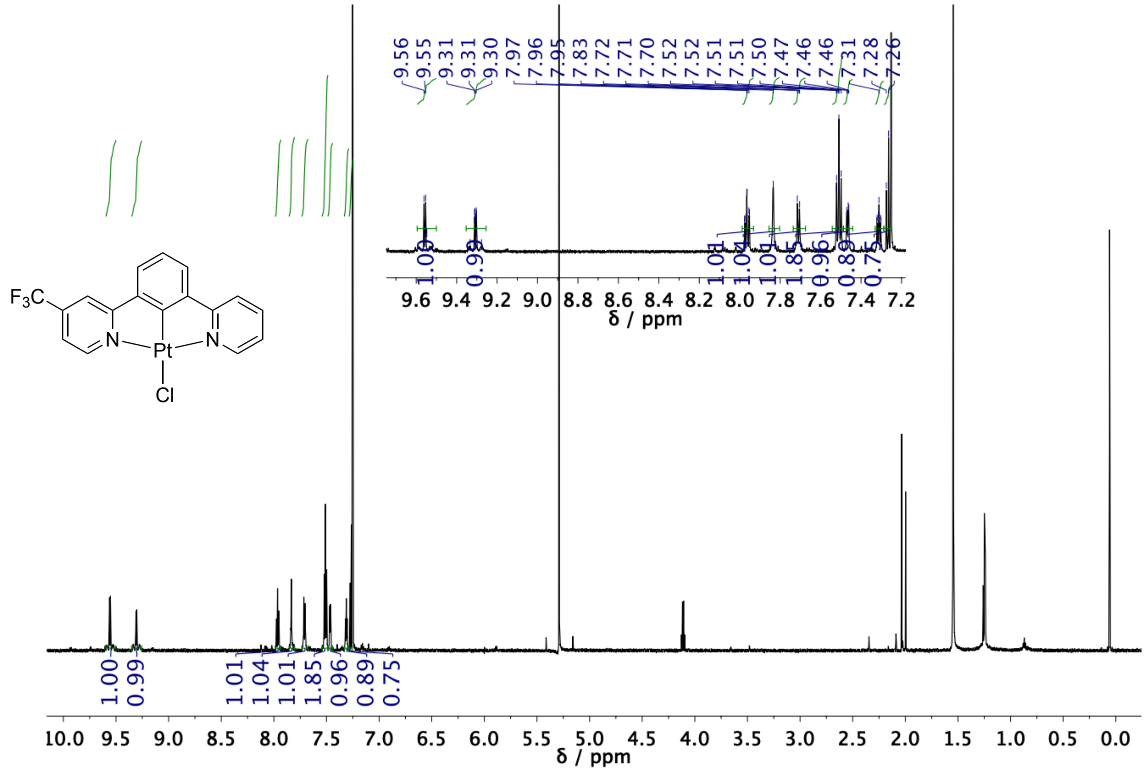

Figure S45 *^{1}H NMR spectrum of PtL[4]Cl in CDCl₃.*

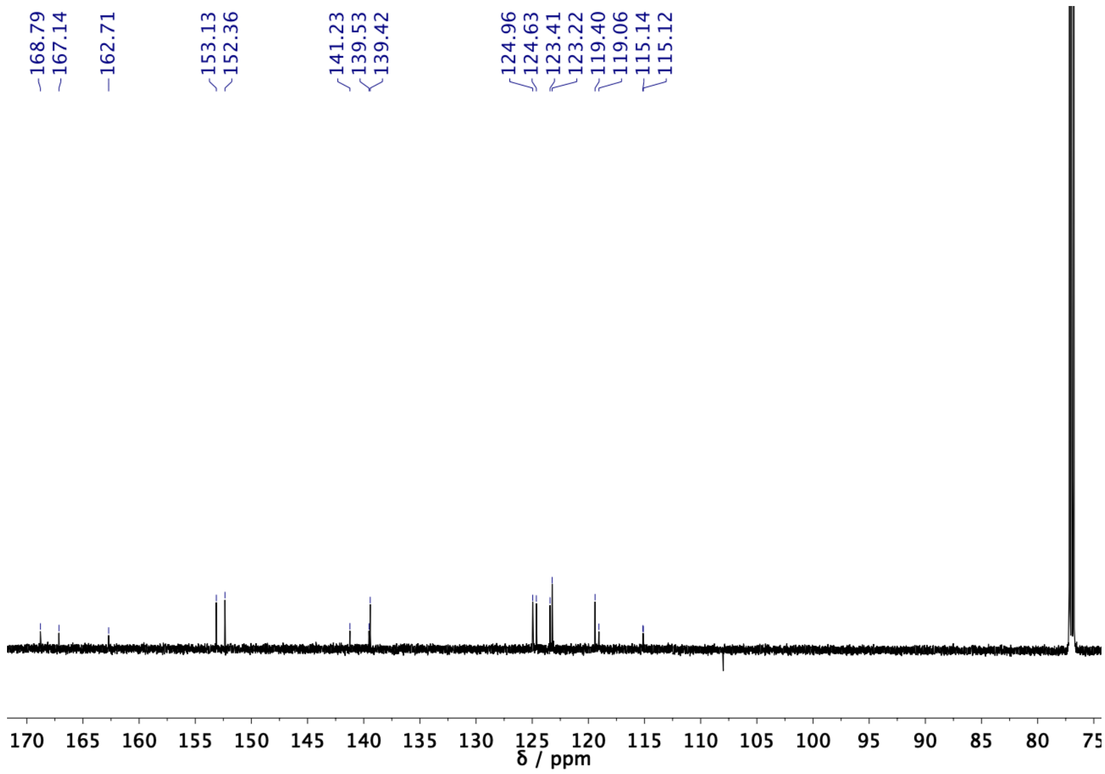

Figure S46 *^{13}C NMR spectrum of PtL[4]Cl in CDCl₃.*

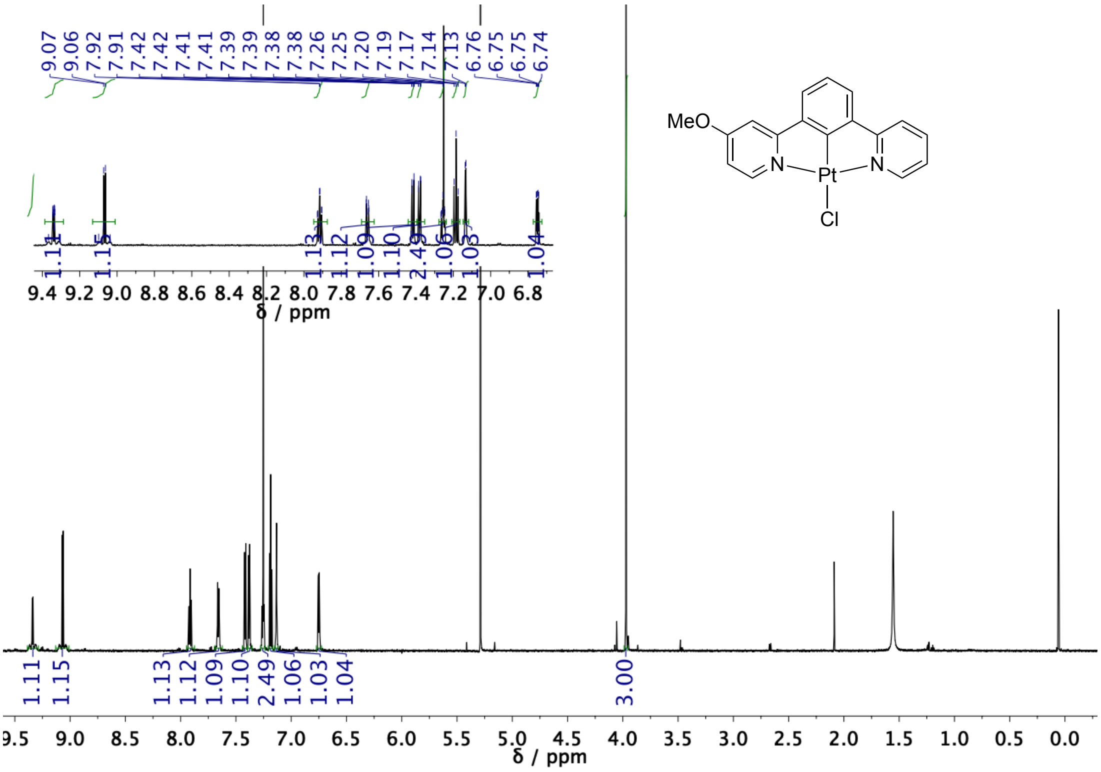

Figure S47 *^{1}H NMR spectrum of PtL[5]Cl in CDCl3.*

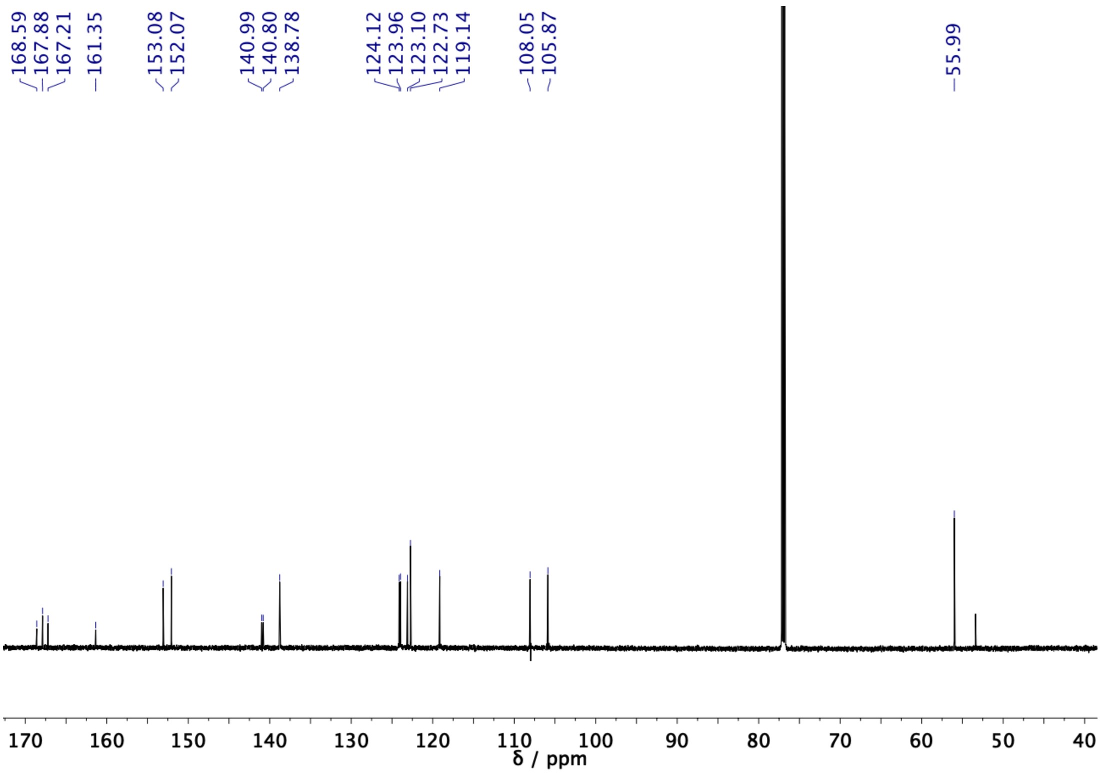

Figure S48 *^{13}C NMR spectrum of PtL[5]Cl in CDCl3.*

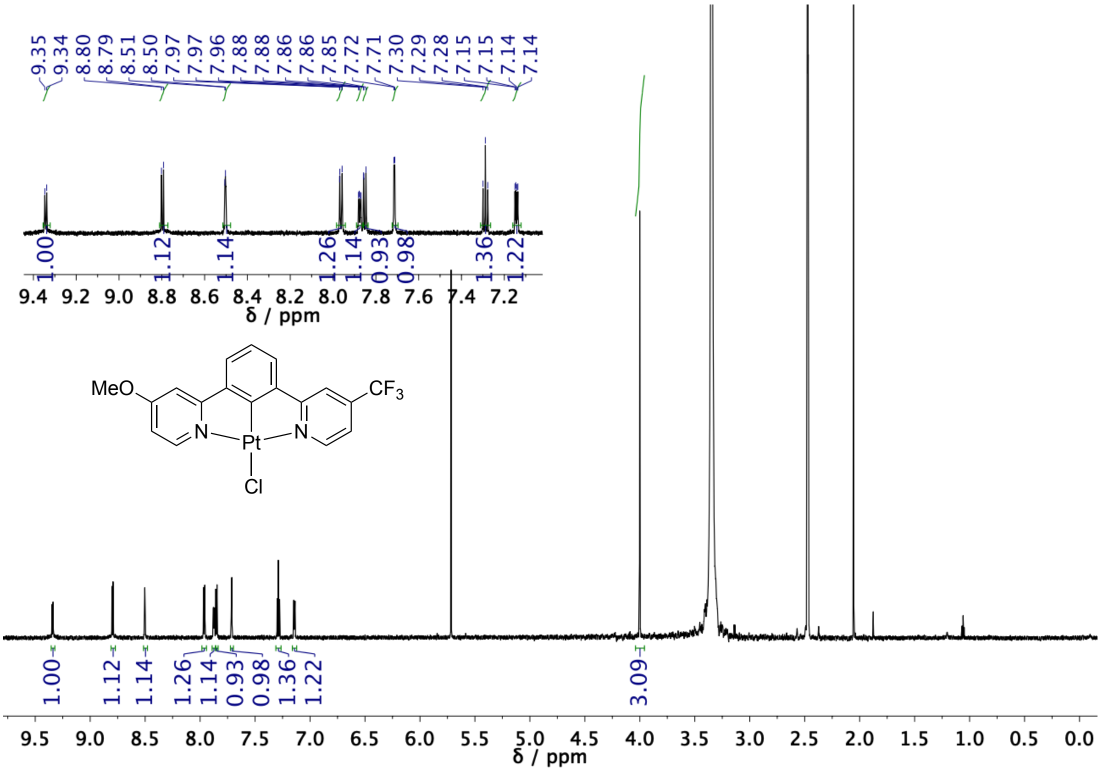

Figure S49 *^{1}H NMR spectrum of PtL[6]Cl in DMSO-d₆.*

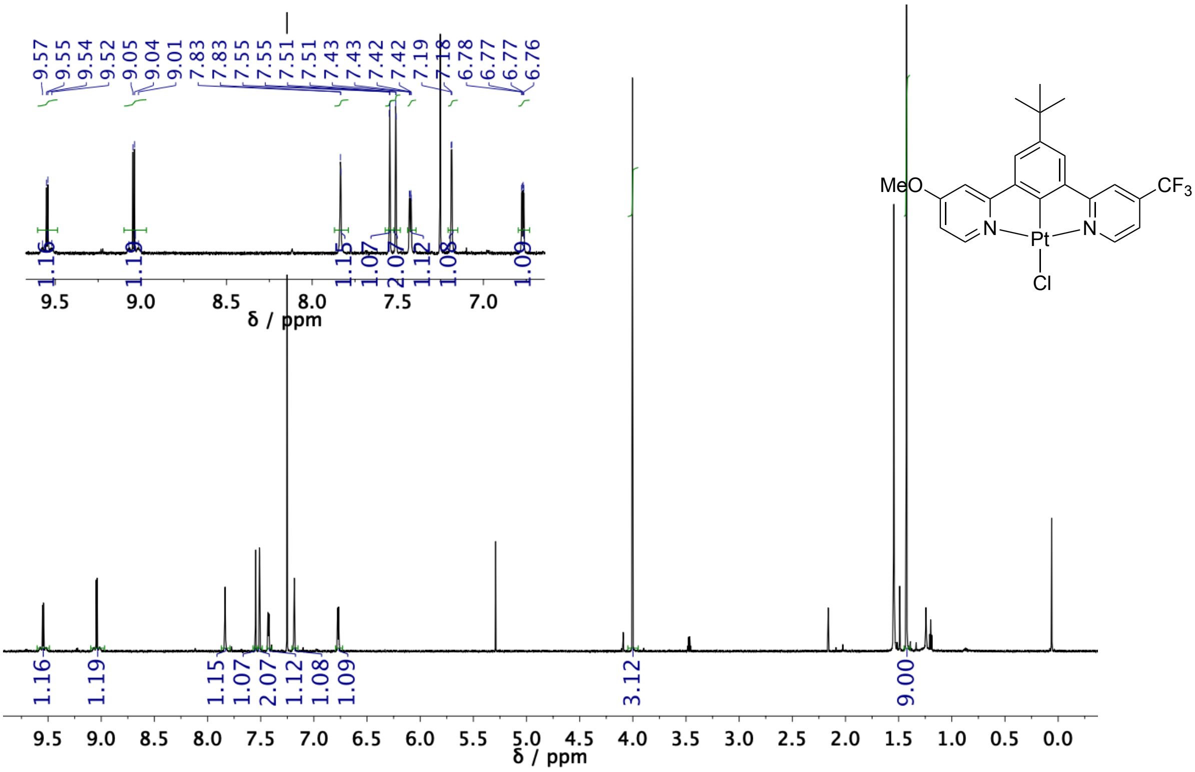

Figure S50 *^{1}H NMR spectrum of PtL[6]*Cl in CDCl₃.*

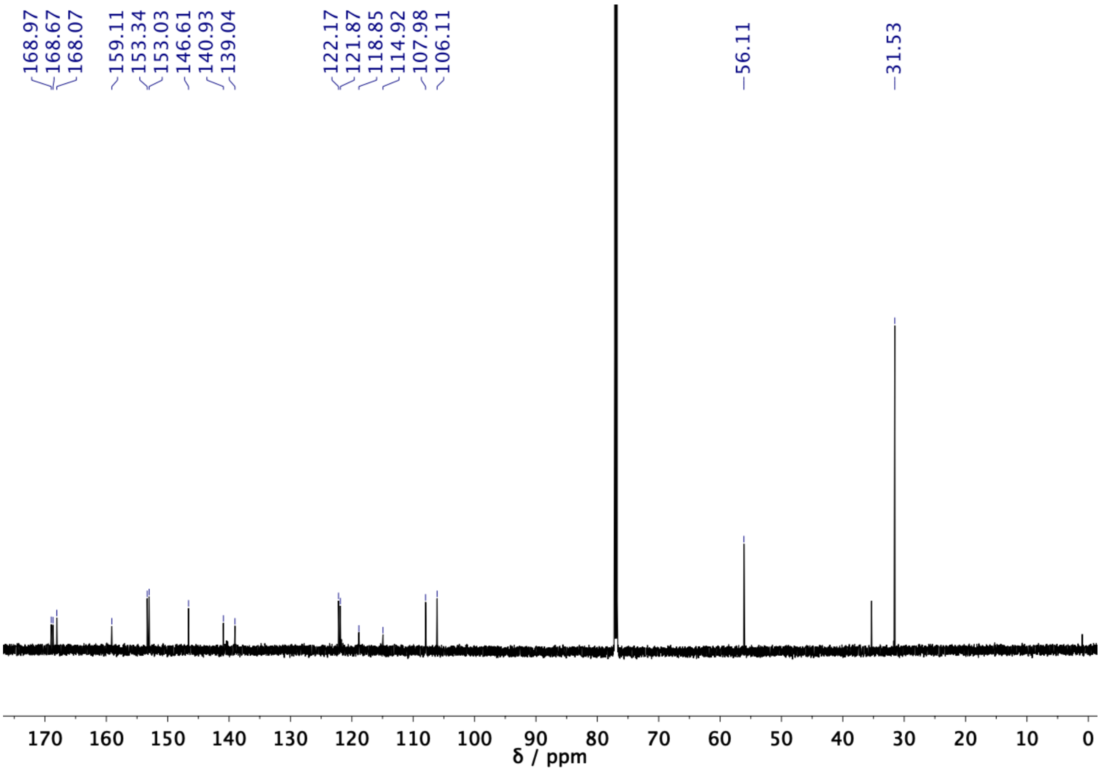

Figure S51 *^{13}C NMR spectrum of PtL^{6}*Cl in $CDCl_3$.*

References for Supporting Information

1. Neese, F. Software update: the ORCA program system, version 4.0. *WIREs Comput. Mol. Sci.* **2018**, *8:e1327*.
2. Neese, F. The ORCA program system. *WIREs Comput. Mol. Sci.* **2012**, *2*, 73–78.
3. Neese, F. Software update: the ORCA program system, version 4.0. *WIREs Comput Mol Sci*. **2022**; *12:e1606.*
4. Lehtola, S.; Steigemann, C.; Oliveira, M. J. T.; Marques, M. A. L. Recent developments in LIBXC – A comprehensive library of functionals for density functional theory. *SoftwareX* **2018**, *7*, 1–5.
5. Pettersen, E.F.; Goddard, T.D; Huang, C.C; Meng, E.C.; Couch, G.S.; Croll, T.I.; Morris, J.H.; Ferrin T.E. UCSF ChimeraX: Structure visualization for researchers, educators, and developers. *Protein Sci.* **2021**, *30*, 70–82.
6. Goddard, T.D.; Huang, C.C.; Meng, E.C.; Pettersen, E.F.; Couch, G.S.; Morris, J.H.; Ferrin, T.E. UCSF ChimeraX: Meeting modern challenges in visualization and analysis. *Protein Sci.* **2018**, *27*, 14–25.
7. Avogadro: an open-source molecular builder and visualization tool. Version 1.2.0, https://avogadro.cc
8. Hanwell, M. D.; Curtis, D. E.; Lonie, D. C.; Vandermeersch, T.; Zurek, E.; Hutchison, G. R. Avogadro: an advanced semantic chemical editor, visualization, and analysis platform. *J. Cheminform.* **2012**, *4*, 17.
9. Becke, A. D. Density-functional thermochemistry. III. The role of exact exchange. *J. Chem. Phys.* **1993**, *98*, 5648–5652.
10. Stephens, P. J.; Devlin, F. J.; Chabalowski, C. F.; Frisch, M. J. *Ab initio* calculation of vibrational absorption and circular dichroism spectra using density functional force fields. *J. Phys. Chem.* **1994**, *98*, 11623–11627.
11. Weigend, F.; Ahlrichs, R. Balanced basis sets of split valence, triple zeta valence and quadrupole zeta valence quality for H to Rn: Design and assessment of accuracy. *Phys. Chem. Chem. Phys.* **2005**, *7*, 3297–3305.
12. Becke, A. D. Density-functional exchange-energy approximation with correct asymptotic behavior. *Phys. Rev. A* **1988**, *38*, 3098–3100.
13. Hellweg, A.; Hättig, C.; Höfener, S.; Klopper, W. Optimized accurate auxiliary basis sets for RI-MP2 and RI-CC2 calculations for the atoms Rb to Rn. *Theor. Chem. Acc.* **2007**, *117*, 587–597.
14. Weigend, F. Accurate Coulomb-fitting basis sets for H to Rn. *Phys. Chem. Chem. Phys.* **2006**, *8*, 1057–1065.
15. Neese, F.; Wennmohs, F.; Hansen, A.; Becker, U. Efficient, approximate and parallel Hartree-Fock and hybrid DFT calculations. A 'chain-of-spheres' algorithm for the Hartree-Fock exchange. *Chem. Phys.* **2009**, *356*, 98–109.
16. Izsák, R.; Neese, F. An overlap fitted chain of spheres exchnage method. *J. Chem. Phys.* **2011**, *135*, 144105.
17. Grimme, S.; Ehrlich, S.; Goerigk, L. Effect of the damping function in dispersion corrected density functional theory. *J. Comput. Chem.* **2011**, *32*, 1456–1465.

18. Grimme, S.; Antony, J.; Ehrlich, S.; Krieg, H. A consistent and accurate ab initio parametrization of density functional dispersion correction (DFT-D) for the 94 elements H–Pu. *J. Chem. Phys.* **2010**, *132*, 154104.
19. Develay, S.; Blackburn, O.; Thompson, A. L.; Williams, J. A. G. Cyclometalated platinum(II) complexes of pyrazole-based, N^C^N-coordinating, terdentate ligands: the contrasting influence of pyrazolyl and pyridyl rings on luminescence. *Inorg. Chem.* **2008**, *47*, 11129–11142.
